# Search for Selection Signatures Related to Trypanosomosis Tolerance in African Goats

**DOI:** 10.3389/fgene.2021.715732

**Published:** 2021-08-03

**Authors:** Bruno Serranito, Dominique Taurisson-Mouret, Sahraoui Harkat, Abbas Laoun, Nadjet-Amina Ouchene-Khelifi, François Pompanon, Badr Benjelloun, Giuliano Cecchi, Sophie Thevenon, Johannes A. Lenstra, Anne Da Silva

**Affiliations:** ^1^Museum National d’Histoire Naturelle, CRESCO, Dinard, France; ^2^University of Limoges, PEREINE, E2LIM, Limoges, France; ^3^University of Limoges, GEOLAB, UMR 6042, Limoges, France; ^4^Science Veterinary Institute, University of Blida, Blida, Algeria; ^5^University of Djelfa, Djelfa, Algeria; ^6^Univ. Grenoble Alpes, Univ. Savoie Mont Blanc, CNRS, LECA, Grenoble, France; ^7^National Institute of Agronomic Research, Regional Centre of Agronomic Research, Beni-Mellal, Morocco; ^8^Food and Agriculture Organization of the United Nations, Animal Production and Health Division, Rome, Italy; ^9^CIRAD, UMR INTERTRYP, Montpellier, France; ^10^INTERTRYP, University of Montpellier, CIRAD, IRD, Montpellier, France; ^11^Faculty of Veterinary Medicine, Utrecht University, Utrecht, Netherlands

**Keywords:** molecular adaptations, local breeds, Sub-Saharan Africa, crossbreeding, tsetse flies

## Abstract

Livestock is heavily affected by trypanosomosis in Africa. Through strong selective pressure, several African indigenous breeds of cattle and small ruminants have acquired varying degrees of tolerance against this disease. In this study, we combined LFMM and PCAdapt for analyzing two datasets of goats from West-Central Africa and East Africa, respectively, both comprising breeds with different assumed levels of trypanotolerance. The objectives were (i) to identify molecular signatures of selection related to trypanotolerance; and (ii) to guide an optimal sampling for subsequent studies. From 33 identified signatures, 18 had been detected previously in the literature as being mainly associated with climatic adaptations. The most plausible signatures of trypanotolerance indicate the genes *DIS3L2*, *COPS7B*, *PD5A*, *UBE2K*, and *UBR1*. The last gene is of particular interest since previous literature has already identified E3-ubiquitin ligases as playing a decisive role in the immune response. For following-up on these findings, the West-Central African area appears particularly relevant because of (i) a clear parasitic load gradient related to a humidity gradient, and (ii) still restricted admixture levels between goat breeds. This study illustrates the importance of protecting local breeds, which have retained unique allelic combinations conferring their remarkable adaptations.

## Introduction

The productivity of the livestock sector in Africa is heavily affected by trypanosomosis, *via* morbidity, mortality, impact on growth and reproduction (Budd, 1999; Kristjanson et al., 1999; Swallow, 2000; Shaw et al., 2017). This vector-borne parasitic infection, also called “Nagana” in domestic animals, is caused by extracellular protozoa that are mainly cyclically transmitted by several species of tsetse flies (*Glossina* spp.). The tsetse distribution area, or tsetse fly “belt,” covers 39 African countries, whose economic development is thus significantly impacted (Alsan, 2015). While many studies have examined trypanosomosis in cattle, this disease has been poorly investigated in sheep and even less in goats (Geerts et al., 2009).

For African goats, the most pathogenic trypanosomes are *Trypanosoma vivax* and *Trypanosoma congolense* (Kusiluka and Kambarage, 1996; Gutierrez et al., 2006). An estimated 173 million of Africa’s 228 million goats live in areas of the continent infested by tsetse flies (Gutierrez et al., 2006). In addition to the economic consequences of trypanosomosis on caprine production (Kanyari et al., 1986), goats could act as a reservoir of trypanosomes for other species, including humans (Informal Expert Group on Gambiense HAT Reservoirs et al., 2018).

The high parasitic pressure in humid to sub-humid dry climates may have played a major role in the historical southward movement of domestic stocks. This movement started at 4,000 to 5,000 YBP on the occasion of major and more favorable environmental changes (Smith, 1992; Newman, 1995). It was probably slowed down by endemic parasitic diseases, so that it reached Southern Africa only around 2000 YBP (Pereira and Amorim, 2010). Under the strong pressure of natural selection that occurred throughout the millennia, indigenous breeds have acquired adaptations that enable them to tolerate various diseases and parasites. In the same way as in cattle and sheep, a few goat breeds are likely to show qualities of tolerance to trypanosomosis. Well defined in cattle, trypanotolerance is a multigenic traits that refers to the ability of some breeds to live and be productive in trypanosomosis endemic areas while susceptible animals usually die without treatments (Murray et al., 1984). Phenotypically, trypanotolerant breeds display lower parasitic loads, maintain higher packed cell volume and are less affected by weight loss during infection (Hanotte et al., 2003; Berthier et al., 2016). In West and Central Africa, about 47% of the goats, the Djallonke or West African Darf breeds that live in tsetse infested areas, are estimated to be in some way trypanotolerant (Mawuena, 1986; Bengaly et al., 1993; Osaer et al., 1994; Agyemang, 2005; Geerts et al., 2009). Trypanotolerance in East Africa populations has been less well studied (Geerts et al., 2009), but some Small East African goat breeds exhibit various trypanotolerance traits (Griffin and Allonby, 1979a,b; Monirei et al., 1982; Murray et al., 1982; Kanyari et al., 1986; Mutayoba et al., 1989; Katunguka-Rwakishaya et al., 1997).

In this study, we used published 50K SNP datasets for sub-Saharan goat populations, including West-Central as well as East African populations, with the objective of identifying molecular signatures of selection related to trypanosomosis tolerance. First, we selected native goats from the Adaptmap dataset, choosing breeds that had been living in tsetse-infested areas for a long time, and of neighborhood breeds living under low tsetse pressure on the other hand. Second, we mapped their distribution area and extracted the data allowing to infer the level of pressure linked to trypanosomes but also the corresponding climatic and topographic variables. Then we searched for selection signatures using PCAdapt (Luu et al., 2017) and LFMM (Frichot et al., 2013) methods. Briefly, PCAdapt uses principal component analysis to describe the population structure and identifies candidate markers as outliers in terms of inferred population structure. The LFMM approach searches for significant associations with environmental factors whilst controlling for the neutral population structure. As complementary methods, we included the Hapflk (Fariello et al., 2013) and the Bayescan (Foll et al., 2010) programs. Finally, (i) we identified plausible candidate genes for trypanosomosis tolerance by analyzing our results in relation to the literature. In addition, (ii) our data may guide an optimization of African goats sampling for follow-up studies.

## Materials and Methods

### Datasets Building

We used the AdaptMap goat dataset (Bertolini et al., 2018), which represents a worldwide coverage of original breeds. Out of the 75 African breeds initially present in the dataset, we selected the 40 indigenous local breeds^1^, to which we added four Algerian breeds (Ouchene-Khelifi et al., 2018; Supplementary Table 1).

On the basis of a NeighborNet visualization (Huson, 1998) of the Reynolds’ distances between these breeds (Supplementary Figure 1) we selected two groups of related breeds in the West-Central Africa and in East Africa where trypanotolerant breeds are located. We retained (i) 190 West-Central African goats from eight local breeds in Burkina Faso, Cameroon, Mali, and Nigeria, including the trypanotolerant West African Dwarf type breeds (Table 1); and (ii) 242 East African goats from 11 local breeds in Burundi, Kenya, Tanzania, and Uganda, including the trypanotolerant Small East African type breeds (Table 2).

**TABLE 1:** West-Central African goat dataset.

**Breed name and acronym (type)**	**Nb. of heads**	**Country**	**Area of the breed in the country**	**Climatic characteristics of the distribution area**	Sampling location* (longitude/latitude)	**Proportion of the distribution area infested by the tsetse flies and ranking for LFMM analysis**
Djallonke: DJA (WAD type)	12	Burkina Faso	Sudan area covering the southern part of Burkina-Faso (from latitude 13°5′N to 11°3′N approximately) Traoré et al., 2009	Mean altitude: 318.1 m Annual mean temperature: 27.6°C Annual precipitation: 959.5 mm	−3.53/10.56	90.01% Rank: 1
Cameroonian: CAM (WAD type)	40	Cameroon	North area of the country MINEPIA, 2000	Mean altitude: 392.2 m Annual mean temperature: 26.8°C Annual precipitation: 959.5 mm	14.39/10.11	27.32% Rank: 2
West African Dwarf: WAD (WAD type)	34	Cameroon	South coastal area MINEPIA, 2000	Mean altitude: 379.7 m Annual mean temperature: 24.9°C Annual precipitation: 2,704.7 mm	10.27/5.9	93.98% Rank: 1
Naine: NAI (WAD type)	17	Mali	Southern part of the country LUKNOW, 1950	Mean altitude: 326.6 m Annual mean temperature: 27.3°C Annual precipitation: 972.3 mm	−7.48/11.42	95.14% Rank: 1
Soudanaise: SDN (Sahelian type)	24	Mali	Central part of the country LUKNOW, 1950	Mean altitude: 287.5 m Annual mean temperature: 28.4°C Annual precipitation: 378.1 mm	−6.27/13.45	6.62% Rank: 3
Red Sokoto: RSK (Sahelian type)	21	Nigeria	Central and northern part of the country Blench, 1999; Meyer, 2020	Mean altitude: 441.7 m Annual mean temperature: 26.0°C Annual precipitation: 1,027.1 mm	8.17/11.89	45.75% Rank: 2
Sahelian: SHL (Sahelian type)	21	Nigeria	Extreme northern part of the country Blench, 1999	Mean altitude: 347.2 m Annual mean temperature: 26.7°C Annual precipitation: 538.9 mm	8.73/11.25	0.26% Rank: 3
West African Dwarf: WADn (WAD type)	21	Nigeria	Southern part of the country Blench, 1999	Mean altitude: 164.3 m Annual mean temperature: 26.7°C Annual precipitation: 1,531.4 mm	3.74/7.59	98.30% Rank: 1

*According to Bertolini et al. (2018): mean altitude in meters, annual mean temperature in degrees Celsius, and annual precipitation in millimeters.

**TABLE 2:** East African goat dataset.

**Breed name and acronym**	**Nb. of heads**	**Country**	**Area of the breed in the country**	**Climatic characteristics of the distribution area**	Sample location* (longitude/latitude)	**Proportion of the distribution area infested by the tsetse flies and ranking for LFMM analysis**
Burundi: BUR	40	Burundi	Highlands Ndayishimiye, 1986	Mean altitude: 1,633.9 m Annual mean temperature: 18.9°C Annual precipitation: 1,279.7 mm	29.83/−2.91	10.92% Rank: 2
Galla: GAL	23	Kenya	Northern arid and semi-arid areas Porter et al., 2016	Mean altitude: 590.6 m Annual mean temperature: 26.7°C Annual precipitation: 337.1 mm	37.66/2.01	18.54% Rank: 2
Small East African: SEAK	31	Kenya	Northern arid and semi-arid areas Porter et al., 2016	Mean altitude: 590.6 m Annual mean temperature: 26.7°C Annual precipitation: 337.1 mm	36.97/0.61	18.54% Rank: 2
Gogo (SEA type): GOG	13	Tanzania	Dodoma region of central Tanzania Nguluma et al., 2016	Mean altitude: 1,254.9 m Annual mean temperature: 21.0°C Annual precipitation: 655.7 mm	36.68/−6.28	18.87% Rank: 2
Maasai: MAA	20	Tanzania	Northern Tanzania along the Great Rift Valley on semi-arid and arid lands Nguluma et al., 2016	Mean altitude: 1,071.3 m Annual mean temperature: 21.4°C Annual precipitation: 663.2	37.23/−4.53	54.44% Rank: 1
Sonjo (SEA type): SNJ	22	Tanzania	Ngorongoro district Nguluma et al., 2016	Mean altitude: 1,520.8 m Annual mean temperature: 20.1°C Annual precipitation: 750.5 mm	36.32/−2.7	55.47% Rank: 1
Small East African: SEAU	15	Uganda	Northern savannah ecological areas and northern part of Buganda Nsubuga, 1994; Onzima et al., 2018	Mean altitude: 1,062.4 m Annual mean temperature: 23.4°C Annual precipitation: 1,283.6 mm	32.07/0.58	47.44% Rank: 1
Karamoja (SEA type): KAR	20	Uganda	Karamoja region Nsubuga, 1994; Onzima et al., 2018	Mean altitude: 1,190.6 m Annual mean temperature: 23.1°C Annual precipitation: 833.0 mm	34.67/2.53	9.47% Rank: 2
Sebei (SEA type): SEB	24	Uganda	Kapchorwa district on the slopes of Mount Elgon Nsubuga, 1994; Onzima et al., 2018	Mean altitude: 1,079.7 m Annual mean temperature: 23.1°C Annual precipitation: 1,163.9 mm	34.45/1.4	13.58% Rank: 2
Mubende: MUB	23	Uganda	Mubende district Nsubuga, 1994; Onzima et al., 2018	Mean altitude: 1,224.0 m Annual mean temperature: 21.5°C Annual precipitation: 1,003.2 mm	32.29/0.44	13.36% Rank: 2
Nganda: NGD	11	Uganda	East of the central province Nsubuga, 1994; Onzima et al., 2018	Mean altitude: 1,139.2 m Annual mean temperature: 21.9°C Annual precipitation: 1,272.4 mm	32.58/0.32	50.87% Rank: 1

*According to Bertolini et al. (2018): mean altitude in meters, annual mean temperature in degrees Celsius, and annual precipitation in millimeters.

### Environmental Characterization

**FIGURE 1:**
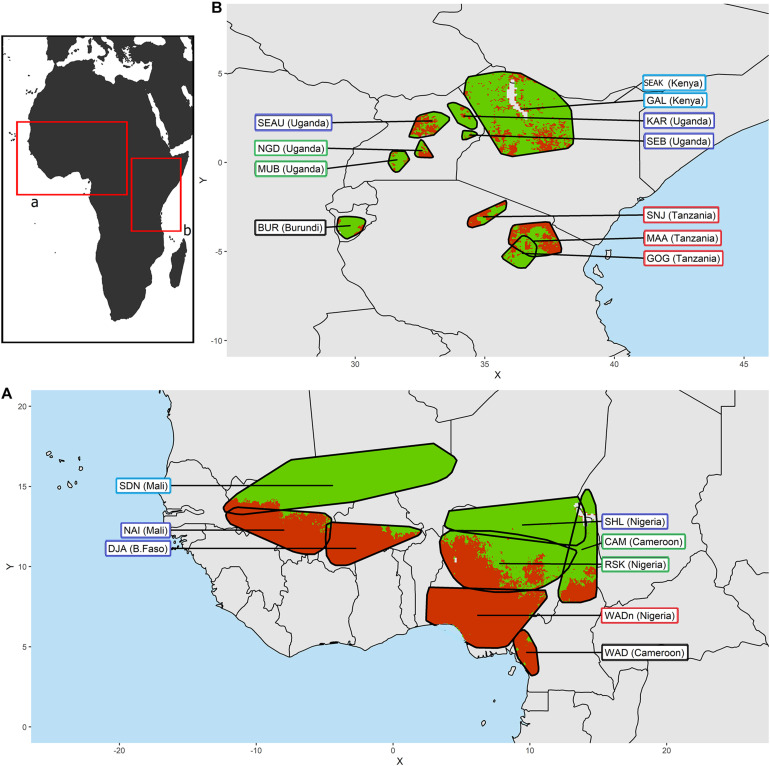
Distribution areas for **(A)** the West-Central African goat breeds and **(B)** the East African goat breeds. In each area, the green color reflects an absence of tsetse flies while the red color identifies their presence according to Wint and Rodgers (2000). The frame of the breed name labels is colored according to the climatic clustering (see details in Figure 2).

We defined the environmental characteristics at the level of the distribution area of the breeds. In West-Central Africa we find in the arid zone, the Sahelian type which is progressively replaced by the West African Dwarf type as it moves down toward the equatorial humid zone. In East Africa, most of the breeds are derived from the Small East African type. From their cradle each type has spread, defining populations or breeds that differ from the basic type by their specificities at the local level (see Tables 1, 2). The breed’s distribution was mapped (Figure 1) *via* ArcGIS (ESRI, 2011). We used the predicted distribution of tsetse flies (*Glossina* species) from the FAO Programme Against African Trypanosomosis (PAAT) to extract the proportion of infestation in each breed’s distribution area (Wint and Rodgers, 2000). The breeds were classified into groups according to the intensity of selection pressure due to trypanosomes transmitted by tsetse flies, detected by this method in their distribution area (Tables 1, 2).

**FIGURE 2:**
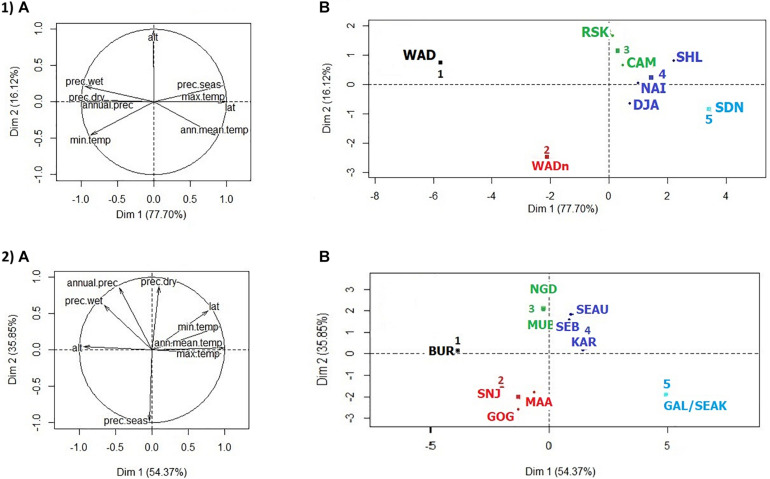
Goat distribution areas analyses as a function of environmental variables. **(1)** HCPC analysis for the West-Central African dataset. **(A)** PCA correlation circle. **(B)** PCA score plot by breed. **(2)** HCPC analysis for the East African dataset. **(A)** PCA correlation circle. **(B)** PCA score plot by breed. Colors represent the main clusters obtained by HCPC (*K* = 5). PC 1 and PC 2 represent respectively principal components 1 and 2. alt., altitude in meters; temp., temperature in °C × 10; prec., precipitation in millimeters; min., minimal; max., maximal; seas., seasonality; lat., latitude; ann. mean, annual mean.

We also used the WorldClim database (v.1.4; Hijmans et al., 2005) to collect bioclimatic data covering the period from 1960 to 1990, with a spatial resolution of 30 arc-seconds in the WGS84 datum. We considered the relevant bioclimatic variables (BIO) to highlight the major temperature and humidity contrasts between the breed distribution areas (see Supplementary Table 2). Altitude information was collected from the SRTM 90 m Digital Elevation Database (v.4.1) (Jarvis et al., 2008). Finally, the latitude was considered as a proxy providing information on luminosity and seasonality. This procedure was performed in R 3.5.2 (R Core Team, 2018) using the R package “RSAGA” (Brenning, 2008).

A PCA analysis was followed by a hierarchical clustering on principal components (HCPC) using Euclidian distances and Ward’s method. Multivariate analyses were performed with R software, using the FactoMineR package (Lê et al., 2008).

Different LFMM analyses were conducted considering (i) the classification of the breeds based on the level of tsetse infestation in their distribution range, and (ii) the classification of the distribution areas obtained through the PCA/HPCP procedure for the climatic variables.

### Genetic Characterization of the Breeds

We used the AdaptMap goat dataset, genotyped with the CaprineSNP50 BeadChip (Dryad^2^) for 53,547 SNPs. SNPs and animals were pruned with PLINK v1.07 (Purcell et al., 2007) using the following parameters: (i) SNP call rate ≤97%, (ii) SNP minor allele frequency (MAF) ≤1%, (iii) animals displaying ≥10% of missing genotypes and retaining 50,796 genotypes for 242 East African goats and 50,459 for 190 West-Central African goats.

### Admixture Analyses

For ADMIXTURE analysis (Alexander et al., 2009), LD-based SNP pruning was carried out using the –indep option of PLINK with the following parameters: 50 SNPs per window, a shift of five SNPs between windows, and a variation inflation factor’s threshold of two (corresponding to *r*^2^ > 0.5). ADMIXTURE was run with *K* = 2–8 for the West-Central goat data-set and K = 2–12, for the East goat dataset, which are the respective numbers of breeds in our datasets. For each value of *K*, 10 independent runs were performed. The entropy criterion was calculated *via* the sNMF function implemented in the R package LEA to assess the number of ancestral populations that best explains the genotypic data (Alexander and Lange, 2011; Frichot et al., 2014). The program CLUMPAK (Kopelman et al., 2015^3^) was used to analyze the multiple independent runs at a single *K* and visualize the results.

### Search for Molecular Selection Signature

We used different methods to identify loci possibly involved in local adaptation:

The PCAdapt approach, through the R pcadapt package, was used to scan the genome and identify outliers considering population structure. Plotting the number of principal components *K* from 1 to 15 using the PCAdapt plot function (Luu et al., 2017), revealed *K* = 5 as optimal number of genetic groups for both data-sets (see Supplementary Figure 2). Candidate SNPs were identified by calculating the false discovery rate (FDR; α = 0.05) of the *p*-values associated with Mahalanobis distance estimated by PCAdapt, using the *q*-value function of the R package *q*-value (Storey and Tibshirani, 2003).

The LFMM latent factor mixed models, developed in the LEA R package (Frichot and François, 2015) was used to test the association genotype-environment, using a linear mixed model. We chose *K* = 1, for the number of latent factors, which take into account for neutral genetic structure, in accordance with the entropy criterion (Lee and Seung, 1999) assessed in the admixture analyses. We performed LFMM using 1,000 sweeps for burn-in and 9,000 additional sweeps, and 10 runs with different seeds. We then chose significant associations based on FDR (α = 0.05) using the R package *q*-value.

BayeScan software V 2.1 (Foll and Gaggiotti, 2008) estimates the posterior probability that a given SNP is the target of selection on the basis on the allele frequencies in each population and using a Bayesian approach *via* Markov chain Monte Carlo (MCMC). The software was set up with 5,000 burn-in interactions, followed by 10,000 interactions with thinning interval of 10. The top candidate SNPs potentially under selection was identified with a false discovery rate of 0.05.

The hapFLK 1.3.0 program^4^ was used to identify signatures of selection accounting for both the hierarchical structure and haplotype information, as described by Fariello et al. (2013). The software was run on a per chromosome basis, using a kinship matrix based on the matrix of Reynolds’ genetic distances between breeds (Bonhomme et al., 2010). No outgroups were defined. The number of haplotype clusters per chromosome was determined in fastPHASE using cross-validation (Scheet and Stephens, 2006) based estimation and was set at 25 clusters (-K 25). The hapFLK statistic was computed for 20 EM runs to fit the LD model (–nfit = 20). *p*-values were generated for each SNP using a chi-square distribution with a python script provided on the hapFLK webpage. A *q*-value threshold of 0.05 was applied to limit the number of false positives. We used the CAVIAR program (Hormozdiari et al., 2014), to identify the best SNPs signals.

For the different analyses, we considered the identified outliers and applied stringent screen to determine selection signatures: we required candidate-selected regions to have at least three outliers SNPs ≤500 kb apart. For LFMM and PCAdapt we added a constraint, imposing that the three outliers (at least) showed *p*-value ≤ 10^–9^. The window chosen was informed by previous evidence that the LD in small ruminant do not exceed 500 kb (see Serranito et al., 2021).

For each analysis, genes within a region spanning 100 kb upstream and downstream of the candidate selection regions were annotated. The chromosomal regions under selection pressure were inspected using NCBI Genome Data Viewer ARS1^5^.

## Results

### Description of the Datasets

#### West-Central African Goats

In the West-Central area we observe three zones with clearly different tsetse infestation patterns (see Figure 1A and Table 1): (i) in the north, the arid zones of the Sahel are not suitable for tsetse flies, so that the Sudanese (SDN) and Sahelian (SHL) distribution areas are almost free of tsetse flies. (ii) The Red Sokoto (RSK) and the Cameroonian (CAM) inhabit areas with intermediate levels of infestations (27 and 45%, respectively). (iii) Naine (NAI), Djallonke (DJA), and WAD breeds from Nigeria and Cameroon inhabit areas with high levels of infestation (i.e., over 90%).

The PCA pattern of climate variables (Figure 2.1) was dominated by the first component (PC1), which accounts for 77.7% of the total variance. The distribution areas appeared thus mainly distributed along a latitudinal South-North axis, following increasing temperature and aridity gradients. PC2 (16.1% of variance) differentiated the zones according to the average altitude. HCPC clustering at *K* = 5 yielded the groups of (1, 2) breeds from southern Nigeria (WADn) and Cameroon (WAD) regions with high rainfall, even during the dry season; (3) breeds at an intermediate latitudinal position and also with an intermediate level of aridity; and (4, 5) breeds subject to low rainfall levels decreasing further close to the Sahelian zone. The levels of tsetse infestation did not match exactly the climatic clustering obtained on the basis of the aridity gradient, but they were strongly related to it.

**FIGURE 3:**
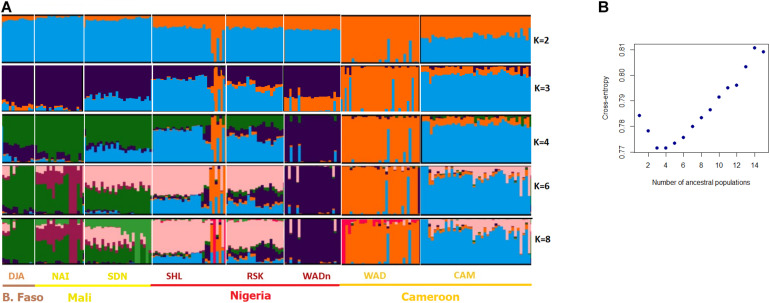
Bayesian clustering performed with ADMIXTURE software on the West/Central African goats. **(A)** Display for different values of *K*, with *K* = number of clusters; **(B)** cross-entropy plot for the number of cluster *K* = 1–15.

For the Admixture analysis of genetic structure (Figure 3), the cross-entropy criterion minimum occurred at *K* = 3 and *K* = 4. The Admixture plot distinguished at *K* = 3 between (i) the Cameroon breed (CAM), (ii) its more southerly compatriot (WAD), (iii) and other breeds from Nigeria, Mali and Burkina-Faso. At *K* = 4, the Nigerian West African Dwarf (WADn) formed a separate cluster. For *K* = 8, levels of admixture were observed between the DJA, NAI, and SDN group on the one hand, and the SHL and RSK group on the other; in such a way that breeds could not be individualized in each group. Finally, several Nigerian SHL individuals clustered with the West African Dwarf of Cameroon (WAD), strongly suggesting introgression.

#### East African Goats

The levels of tsetse infestation showed little contrast between the breed distribution areas of East Africa (see Figure 1B and Table 2). We distinguished two groups: (i) breeds in areas with low to moderate infestation rates (below 20%), and (ii) breeds for which infestation rates of living areas were around 50% (i.e., Massai MAA, Sonjo SNJ, Small East Africa from Uganda SEAU, and Nganda NGD).

In the climatic PCA analysis (Figure 2.2), PC1 accounted for 54.4% of the variation and PC2 for 35.9%. PC1 differentiated the areas according to altitudinal and temperature gradient. On the second axis, the areas were distinguished according to the seasonality of rainfall. HCPC clustering, considering *K* = 5 as the number of clusters, was as follows: group 1 concerned Burundi (BUR) with a distribution area characterized by a high average altitude and consequently low temperatures. Average temperatures and altitudes and a pronounced seasonality of rainfall characterized living areas of group 2, which included Tanzanian breeds; while group 3, including two Ugandan breeds, differed from the previous one mainly by the occurrence of rain even during the dry season. Group 4, also in Uganda, concerned areas with low seasonality as in the previous group but with higher average temperatures and correlatively lower average altitudes. Finally, group 5 included the Kenyan breeds and was characterized by high average temperatures and seasonal rainfall.

**FIGURE 4:**
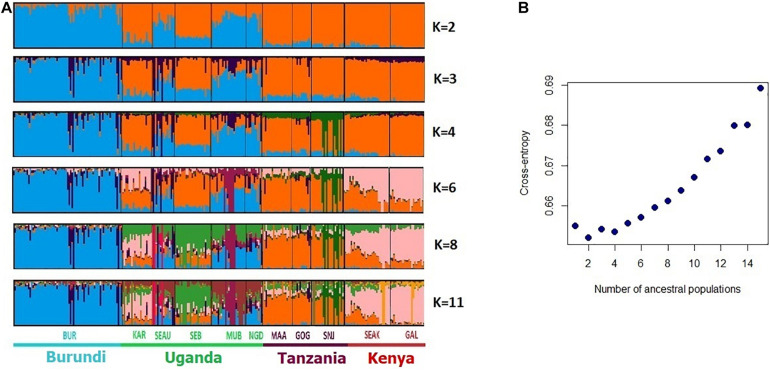
Bayesian clustering performed with ADMIXTURE software on the East African goats. **(A)** Display for different values of *K*, with *K* = number of clusters; **(B)** cross-entropy plot for the number of cluster *K* = 1–15.

Concerning the analysis of the genetic structure (Figure 4), the cross-entropy criterion minimum occurred at *K* = 2, indicating a major divergence of the Burundi breed (BUR) and the other breeds. For *K* = 3 and *K* = 4 sub-structuring was shared by (i) breeds from Tanzania and Kenya KAR and SEB from Uganda; (ii) the three other breeds from Uganda (SEAU, MUB, and NGD). At *K* = 11, the breeds of Uganda, Tanzania, and Kenya still did not form separate clusters. High levels of admixture were observed between (i) SEAK, GAL, and KAR breeds, combining breeds from Kenya and Uganda, (ii) between the Ugandan SEAU, MUB, and NGD breeds, and (iii) between the Tanzanian, MAA, GOG, and SNJ breeds.

### Selection Signatures in West and Central African Goats

The LFMM analysis (Table 3, see details in Supplementary Figure 3 and Supplementary Table 3) based on the correlation with tsetse flies’ infestations ranking (Table 1) allowed the detection of five selection signatures. Among them, the signature near the *Neurobeachin* (*NBEA*) gene was also identified by Bayescan, Hapflk, and the LFMM analysis based on the correlation with the PCA/HCPC climatic ranking (Figure 2.1, see details in Supplementary Table 4 and Supplementary Figure 4). Moreover, it was found to be associated with temperature adaptation in sheep and goats around the world (see details in section “Discussion”). From the other four signatures, the signature near the *SLC34A2* gene was previously associated with high altitude conditions in cattle (Verma et al., 2018), and identified by Serranito et al. (2021) as associated with climatic adaptation in Mediterranean sheep and goat. *SOCS2* was found associated with hypoxia condition in goat and sheep (Wang et al., 2016; Yang et al., 2016). This leaves two signatures, potentially related to trypanosomosis tolerance and targeting the genes: *DIS3L2, COPS7B, RAPGEF6*, and *MEIKIN*, respectively. The latter signature was also detected by LFMM analysis correlating genomes and PCA/HCPC climatic ranking (see details in Supplementary Table 4 and Supplementary Figure 4). This analysis detected a total of 10 selection signatures potentially correlated to climatic conditions, including four signatures on chromosome 7.

**TABLE 3:**
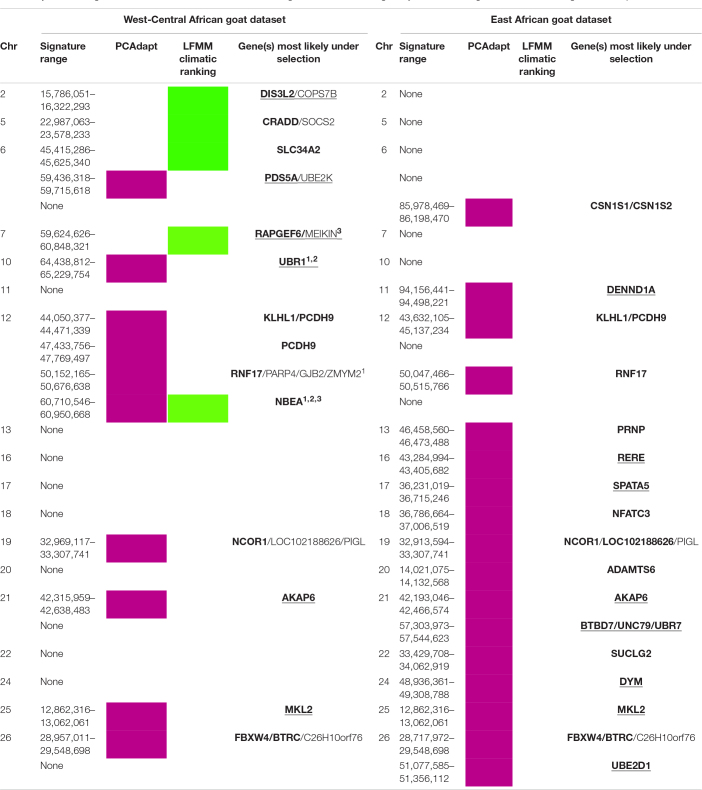
Selection signatures identified in the West-Central African goat and East-African goat by LFMM following the infestation ranking and PCAdapt.

*Each signature was named according to the gene(s) most likely targeted by the selection process, taking into account the distance of the SNPs from the genes, the associated p-values, and the number of SNPs near or in the gene(s); in bold, the most likely targeted genes when there are several. All annotated genes associated with each selection signature are highlighted in Supplementary Table 3.*

*^1^Identified by Bayescan.*

*^2^Identified by HAPFLK.*

*^3^Identified by LFMM considering the climatic gradient.*

*Signatures underlined were not identified in the literature as linked to climatic adaptation. Chr.: chromosome number.*

PCAdapt (Table 3, see details in Supplementary Table 3) identified 10 selection signatures, which include *NBEA* already found by LFMM. From the other nine signatures, those including genes *PCDH9, KLH1, RNF17, NCOR1*, and *BTRC* have been found in the literature as associated with climatic conditions (Wang et al., 2015; Kim et al., 2016; Mdladla, 2016; Yang et al., 2016; Mwacharo et al., 2018; Serranito et al., 2021). This leaves four signatures, targeting *PDS5A* and *UBE2K, AKAP6, MKL2*, and *UBR1*, that are potentially related to trypanotolerance. This last signature was also identified by Bayescan and Hapflk/Caviar. The Hapflk population tree constructed in the *UBR1* region under selection (Supplementary Figure 4) indicated that Cameroonian breeds (WAD and CAM) were particularly targeted by the selected haplotypes.

### Selection Signatures in East African Goats

The analyses for this dataset did not detect any selection signatures *via* the LFMM analyses either, by the correlation with the infestation ranking (Table 2) or by the correlation with the PCA/HCPC climatic ranking (Figure 2.2). PCAdapt (Table 3, see details in Supplementary Table 3 and Supplementary Figure 5) allowed the detection of 17 selection signatures, nine of which were previously detected in the literature as associated with agronomic traits, *CSN1S1* and *CNS1S2* (see Martin et al., 2002 for a review), disease/immunity, *PRNP* (see Greenlee, 2019 for a review), and *NFATC3* (Minematsu et al., 2011) or climate variations. In detail, signatures targeting *KLH1, PCDH9, RNF17, NCOR1*, and *BTRC* and potentially implicated in climate adaptation were also found in the West-Central dataset, while *ADAMT6* (Wu et al., 2019; Serranito et al., 2021), and *SUCLG2* (see details in section “Discussion”) were only found in the East dataset. This leaves the signatures near *DENND1A, SPATA5, RERE, BTBD7* with *UNC79* and *UBR7, DYM, UBE2D1, AKAP6*, and *MKL2*, respectively, as candidates for trypanotolerance; the last two were also identified in the West-Central African dataset.

## Discussion

We studied two datasets including goat breeds with different degrees of trypanotolerance from West-Central and East Africa, respectively, in order to identify selection signatures potentially related to trypanosome tolerance.

### Relevance of Datasets

The datasets appeared to differ in several aspects in terms of environment and molecular-genetic patterns. West-Central African breeds showed contrasting habitats in terms of tsetse flies infestation correlating with the PCA/HCPC climatic ranking, and following mainly the humidity and temperature gradients. Indeed, this is the main determinant of land cover that provides suitable habitat for tsetse flies (Cecchi et al., 2008), even if modified by for instance riverine vegetation and local habitat destruction or fragmentation (Bouyer et al., 2015).

In contrast, the East African breeds inhabit areas with more moderate or patchier levels of tsetse flies’ infestation. The PCA climatic analysis highlighted areas of goat breeds distribution tangled between a temperature gradient on the one hand, largely influenced by altitude, and the seasonality of rainfall on the other hand. Areas of high altitudes that are found in East Africa (i.e., above roughly 2,000 m) are unsuitable for tsetse flies’ survival (Slingenbergh, 1992; Cecchi et al., 2015). In addition, climatic zones are globally drier than in West and Central Africa and harbor different tsetse species with also different ecological requirements (Cecchi et al., 2008).

Our molecular analysis showed for the West-Central African dataset breeds that in spite of varying degrees of admixtures have kept their genetic identity. In East Africa only goats in Burundi were clearly distinct from other goats, while goats from Uganda, Tanzania, and Kenya were largely admixed.

The history of pastoralism has been documented in Kenya and northern Tanzania from ∼3300 BP (Marshall et al., 2011; Gifford-Gonzalez, 2017; Grillo et al., 2018). For several millennia the Bantu, Nilotic, and Khoisan-speaking tribes have evolved in these environmentally contrasting regions of East Africa, allowing the emergence of a wide diversity of goat breeds adapted to the environmental conditions (Mason, 1969; Ahuya, 1997). The colonial period induced profound shifts in traditional pastoral patterns (Masefield, 1962; Lwanga-Lunyiigo, 1987; Chacha, 1999). In particular, it has been tried to improve the productivity of the goats, essentially made up of small breeds of the Small East African type. In the 1980s, a turning point was observed in East Africa with a strong encouragement to crossbreed with imported exotic breeds, including Boer, Kamorai, Toggenburg, Saanen, Norwegian, Alpine, and Anglo-Nubian (Das and Sendalo, 1991). Major programs were set up at various levels and involved: religious organizations, government institutions, non-governmental organizations, such as Heifer International, Bill and Melinda Gates Foundation, British-Farm Africa, Livestock Production Programme, German-GTZ, etc., and academic research institutes (Wilson et al., 1990; Bill and Melinda Gates Foundation (BMFG), 2013; Mruttu et al., 2016). For instance, the “Small Ruminant – Collaborative Research Support Programme” was established in 1980 by the government of Kenya and the United States of America International Development Agency. The objectives were to establish a dual purpose goat (DPG) by a four-way cross of Toggenburg, Galla, Anglo-Nubian, and Small East Africa (Ruvuna, 1986), and associated production systems, in order to increase milk and meat production in western Kenya (Rewe et al., 2002; Bett et al., 2007). In Tanzania, in 1996, another DPG project used “Blended goats” (Kamorai 55%, Boer 30%, and indigenous Tanzania Goats 15%) × Anglo-Nubian which were transferred to smallholder farmers (Shirima, 2005).

Upgrading local goats by cross-breeding is still the prevailing strategy in East Africa, in spite of the limited results obtained in terms of productivity and the genetic erosion caused to the native gene pool (Ahuya, 1997; Gichohi, 1998; Ayalew et al., 2003; Onzima, 2014; FAO, 2015; Mruttu et al., 2016; Wilson, 2018). These considerations provide an understanding of the levels of admixture observed, which are corroborated by additional admixture analyses (see Supplementary Text 1).

In the East African dataset, the non-identification of selection signatures related to the climatic environment by the LFMM method was unexpected, especially in view of the strongly contrasting environments, in particular in terms of altitude and the finding of 10 environmental selection signatures in the West-Central African dataset. The East-African dataset differs from the West-Central one, in terms of admixture levels. It can be hypothesized that cross-breeding with exotic breeds populations has obscured the link between genome and environment, but this remains to be tested with larger datasets.

Moreover, the East African dataset did not reveal, *via* LFMM, any selection signatures related to the infestation rate by tsetse flies. Given the low contrasting levels of infestation across the breeds, this may be explained by a lack of statistical power. However, the high level of admixture may also have played key role. It has been reported that trypanosome tolerance levels were reduced in the descendants of crosses between trypanotolerant East-African goat and exotic breeds (Griffin and Allonby, 1979a,b), although this was not observed with Kenyan breeds (Whitelaw et al., 1985).

For the West-Central Africa region, the oldest goat remains date from about 2300 BC to 1900 BC (Blench et al., 2006). The West African Dwarf type has diversified into heterogeneous populations in tropical and equatorial areas and has developed trypanotolerance, in contrast with the Sahelian type, which has evolved in dry to arid environments (Adah et al., 1993; Bengaly et al., 1993; Osaer et al., 1994; Geerts et al., 2009). Several authors reported that crosses between West African Dwarf type and Sahelian goats are common over the last few decades (Trail et al., 1980; Blench, 1999; Hoeven et al., 2006). Our admixture analysis indicated that this has particularly affected the dwarf breeds of Burkina-Faso and Mali. Goossens et al. (1999) showed a decrease in trypanotolerance for crosses between West African Dwarf type and Sahelian type. Introgression of non-trypanotolerant genes in West African Dwarf types may explain the limited power of our analysis, which identified *via* the LFMM method only five selection signatures related to the level of infestation.

Finally, we note that the Bayescan and Hapflk softwares, which are sensitive to admixture, only showed a weak number of signatures and were not particularly suitable to these datasets, with local heterogeneous breeds. Moreover, it is interesting to note that for the East African dataset with the higher admixture levels and less outspoken trypanotolerance contrast, only PCAdapt retained enough power to identify selection signatures.

### Selection Signatures

In all, considering the two datasets, with the PCAdapt and LFMM methods according to the climatic ranking and the level of infestation ranking, 41 selection signatures were identified, 33 of which were common to both datasets or were highlighted by different methods. Of these signatures, 18 have been detected previously in the literature, as mostly linked to climatic adaptation; and in particular, they were all identified in Serranito et al. (2021), except the signature targeting *SOCS2*.

#### Selection Signatures Unlikely to Be Trypanosome-Related

Considering the 18 signatures in detail, we identified *via* all four method, one selection signature near the *NBEA* gene in West and Central African goats. *NBEA* was reported to be associated with high altitude in Ethiopian sheep (Edea et al., 2019), Chinese sheep (Yang et al., 2016), cattle at high altitude (Zheng and Shabek, 2017), and yaks (Qi et al., 2019). Furthermore, it was found under selection in cattle (Howard et al., 2013), in Ugandan and Moroccan goats (Onzima et al., 2018; Benjelloun, 2015) and in Mediterranean sheep and goats (Serranito et al., 2021). This latest study postulates that this gene may play a predominant role in climate adaptation.

Four of the other 17 selection signature were found in both datasets: (i) *FBXW4* and *BTRC* involved in lipid metabolism (Ishimoto et al., 2017); (ii) *NCOR1* belonging to clock circadian gene network in cattle (Wang et al., 2015); (iii) *RNF17* associated with fatty acid composition (Lemos et al., 2016), growth traits (Puig-Oliveras et al., 2014; Edea et al., 2018) and adaptation to climate variables in South African goats (Mdladla, 2016); and (iv) *KLH1* and *PCDH9*, a major signature related to adaptation to aridity (Kim et al., 2016; Serranito et al., 2021).

Most of the remaining signatures were found only in the East African dataset: *PRNP* linked to susceptibility to prion disease in small ruminants (see Greenlee, 2019 for a review), *NFATC3* involved in immunity (Minematsu et al., 2011), the milk genes *CSN1S1* and *CSN1S2* (see Martin et al., 2002), and two genes implicated in altitude adaptation, *ADAMTS6* and *SUCLG2*. This is not surprising as the East African breeds under consideration live at mean altitudes varying from 590 meters in Kenya to 1,633 m in Burundi (Supplementary Table 5). *ADAMTS6* is involved in porcine growth traits (Wu et al., 2019) and is associated with high altitude adaptation in Chinese sheep; whereas *SUCLG2* was found to be associated with climatic conditions in Egyptian, Chinese, and Mediterranean sheep (Kim et al., 2016; Yang et al., 2016; Serranito et al., 2021), but also in yaks (Qi et al., 2019). Finally, *SLC34A2* implicated in production and recycling of breath surfactant, in humans (Ma et al., 2018), was identified in the West-Central goat dataset, together with *CRADD* and *SOCS2*. The *SOCS2* protein was found associated with hypoxia condition, in Chinese goat and Tibetan sheep (Wang et al., 2016; Yang et al., 2016), but has also been shown to modulate the innate and adaptive immune response in various experimental models of infection, including *Toxoplasma gondii*, *Trypanosoma cruzi*, and *Plasmodium berghei ANKA* (Machado and Aliberti, 2006; Esper et al., 2012; Machado et al., 2012; Brant et al., 2016).

#### Selection Signatures Potentially Linked to Trypanosomosis Tolerance

The most promising signatures have all been identified in the West and Central African dataset and included the following genes: *DIS3L2, COPS7B, PD5A, UBE2K*, and *UBR1*. There is no literature on *AKAP6* and *MKL2*, but these signatures found in both datasets could indicate a significant role for these genes.

The selection signature potentially targeting *UBR1* (*E3 ubiquitin-protein ligase component n-recognition*) was highlighted by PCAdapt, Bayescan, and Hapflk/Caviar. E3 ubiquitin-protein ligases are involved in many cellular processes in eukaryotes (Zheng and Shabek, 2017). Moreover, the E3-Ubiquitin ligase MARCH1 was shown to regulate type I interferon signaling, T cell activation, and IFN-γ production during malaria infections and was proposed to be a key molecule in immune responses and a potential target for immunotherapies (Wu et al., 2020). Furthermore, Kim et al. (2017) identified a selection signature targeting *EPB42*, which is close to *UBR1* and is involved in pathways controlling anemia, in the trypanotolerant N’Dama cattle.

Interestingly, in addition to *UBE2K* and *UBR1*, also the ubiquitins *UBE2D1* and *UBR7* have been indicated by our study, as being subject to selection. Moreover, the degradation of *NFATC3*, also identified by this study, which plays key role in immunity, is ubiquitin-dependent. Indeed, ubiquitination plays a central role in the regulation of various biological functions including immune responses (Hu and Sun, 2016).

The human *COPS7B* protein level has been linked to the invasion efficiency of *T. gondii* (Ready, 2013). *DIS3L2*, located near *COPS7B*, is an RNA-binding protein with 3′-5′ exoribonuclease activity and plays an important role in cytoplasmic RNA surveillance and decay. Becker et al. (2020) suggested a potential link between the gene *DIS3L2* and the gastrointestinal nematode resistance in sheep and postulated that *DIS3L2* polymorphisms influence its degradation functions at the oligoU tail and hence modulate immune response to parasite infection. *DIS3L2* has also been identified, among genes involved in cancer, cellular function and maintenance, and neurological disease, in West African cattle (Gautier et al., 2009). In Brazilian sheep, De Simoni Gouveia et al. (2017) found it associated with height variation.

A selection signature near the genes *PD5A* and *UBE2K* has previously been associated with the bovine resistance to endoparasites and more specifically with the antibody response to *Fasciola hepatica* (Kim et al., 2015; Twomey et al., 2019). Capalbo et al. (2012) suggested a role of *PDS5A* in HIV-1-induced cellular pathogenesis. The gene *UBE2K* was found associated with the immune response to a malaria-like parasite in a wild primate (Trujillo and Bergey, 2020).

We were able to identify selection signatures associated with climatic variables and the presence of tsetse flies, the cyclical vector of trypanosomes. Like tsetse flies whose geographic distribution depends on land cover and climate, other selective pressures are also associated with environmental factors and could thus overlap at least partly with nagana. Especially, numerous parasites and pathogenic agents are differentially distributed according to geographic and climatic patterns in Sub-Saharan Africa, like gastro-intestinal parasites (Bolajoko and Morgan, 2012), ticks (Walker et al., 2003; Silatsa et al., 2019) and *Cowdria ruminantium* (Esemu et al., 2011) transmitted by *Amblyomma* ticks. Genetic tolerance of goats to haemonchosis (Chiejina et al., 2015; Silva et al., 2018) and heartwater (Bambou et al., 2010; Donkin et al., 2015) has been reported. Therefore, the highlighted signatures cannot yet be associated exclusively with any of these diseases. Whereas co-infections between different pathogens are highly prevalent in Sub-Saharan Africa (Kusiluka and Kambarage, 1996), infection by a pathogen can modulate, in a positive or negative way, the host’s response to subsequent infections by other pathogens, suggesting complex interacting processes (Faye et al., 2002; Gonçalves et al., 2014). Moreover, some trade-offs could exist, since contrary to response to trypanosomosis, WAD goats could be more susceptible to Peste des Petits Ruminants, caused by a morbillivirus, than Sahelian populations (Diop et al., 2005). In any case, several genes targeted by selection signatures have been reported throughout the literature to be involved in immune mechanisms concerning different pathogens, mainly nematodes, platyhelminthes, or protozoan parasites.

## Conclusion

We observed a number of selection signatures that could be potentially related to trypanosomosis tolerance in goats. The most consistent signature appears to involve the ubiquitination by the *UBR1* gene, highlighted by all the methods used. Furthermore, the analysis of these two populations highlights that (i) the environmental conditions in West and Central Africa were characterized by a clear gradient related to the climate and land cover; and (ii) that West and Central African goats have remained relatively protected from inter-breeding crosses. These elements make this area of Africa a region of choice to study selection signatures linked to trypanosomosis. It will therefore be of particular interest to deepen these analyses by genotyping other breeds living in the different areas of contrasting parasitic pressures and to document epidemiological and environmental factors and breeding systems as well. All this emphasizes the importance of preserving indigenous stocks and their adaptation as legacy of thousands of years of evolution, instead of pursuing programs with short-term productive objectives. Finally, extending these analyses to sheep breeds will be most enlightening.

## Data Availability Statement

Publicly available datasets were analyzed in this study. This data can be found here: genotyping data collected by AdaptMap (http://www.goatadaptmap.org/) and shared on Dryad (https://doi.org/10.5061/dryad.v8g21pt).

## Author Contributions

BS, SH, AL, N-AO-K, and BB contributed to the data analyses. DT-M performed the historical analyses. GC, ST, FP, and JAL contributed to the review and editing. AD designed the research, analyzed the data, and wrote the manuscript. All authors contributed to the final manuscript.

## Conflict of Interest

The authors declare that the research was conducted in the absence of any commercial or financial relationships that could be construed as a potential conflict of interest.

## Publisher’s Note

All claims expressed in this article are solely those of the authors and do not necessarily represent those of their affiliated organizations, or those of the publisher, the editors and the reviewers. Any product that may be evaluated in this article, or claim that may be made by its manufacturer, is not guaranteed or endorsed by the publisher.

## Footnotes

1
www.fao.org/dad-is

2
https://doi.org/10.5061/dryad.v8g21pt

3
http://clumpak.tau.ac.il

4
https://forge-dga.jouy.inra.fr/projects/hapflk

5
https://www.ncbi.nlm.nih.gov/genome/gdv/

## Supplementary Material

Supplementary Figure 1 NeighborNet graph considering African local goat breeds, from a matrix of Reynolds’ distances.

Supplementary Figure 2 PCAdapt screen plots, **(a)** considering the West-Central African goat dataset, **(b)** considering the East African goat dataset.

Supplementary Figure 3 Display of the selection signatures identified in the West-Central African goat dataset. The figures have been modified from outputs obtained *via* NCBI Genome Data Viewer (https://www.ncbi.nlm.nih.gov/genome/gdv/): ARS1. SNPs under selection (see their name and position in Supplementary Table 3) are symbolized by stars; red stars: SNPs identified by PCAdapt (and plots associated), green stars: SNPs identified by LFMM following the infestation ranking (and plots associated); Hapflk plot and tree displays.

Supplementary Figure 4 Display of the selection signatures identified in the West-Central African goat dataset *via* LFMM following the climatic PCA/HCPC ranking. The figures have been modified from outputs obtained *via* NCBI Genome Data Viewer (https://www.ncbi.nlm.nih.gov/genome/gdv/), assembly ARS1. SNPs under selection (see their name and position in Supplementary Table 4) are symbolized by stars.

Supplementary Figure 5 Display of the selection signatures identified in the East African goat dataset. The figures have been modified from outputs obtained *via* NCBI Genome Data Viewer (https://www.ncbi.nlm.nih.gov/genome/gdv/): ARS1. SNPs under selection (see their name and position in Supplementary Table 3) are symbolized by stars; red stars: SNPs identified by PCAdapt (and plots associated); green stars: SNPs identified by LFMM following the infestation ranking (and plots associated).

Supplementary Table 1 Initial list of breeds for the constitution of the datasets.

Supplementary Table 2 Presentation of the bioclimatic variables used.

Supplementary Table 3 Details concerning the selection signatures identified in West-Central African and East African goats: name and position of the SNPs, genes identified according to PCAdapt, LFMM following the infestation ranking, Bayesca, and Hapflk/Caviar.

Supplementary Table 4 Details concerning the selection signatures identified in West-Central African goats: name and position of the SNPs, genes identified according to LFMM following the climatic PCA/HCPC ranking.

Supplementary Table 5 Summary of environmental data extracted for the distribution area of the West-Central African and East-African goat breeds.

Supplementary Text 1 East African admixture analysis, including exotic breeds and crossbred individuals.
