# Supplementary material for: Search for Selection Signatures Related to Trypanosomosis Tolerance in African Goats
Source: Front Genet. 2021 Aug 3;12:715732. doi: 10.3389/fgene.2021.715732 (PMC8369930; doi:10.3389/fgene.2021.715732)
Supplement: Supplementary Figure 1 — NeighborNet graph considering African local goat breeds, from a matrix of Reynolds’ distances. [file Data_Sheet_1.pdf]

Supplementary Figure 1

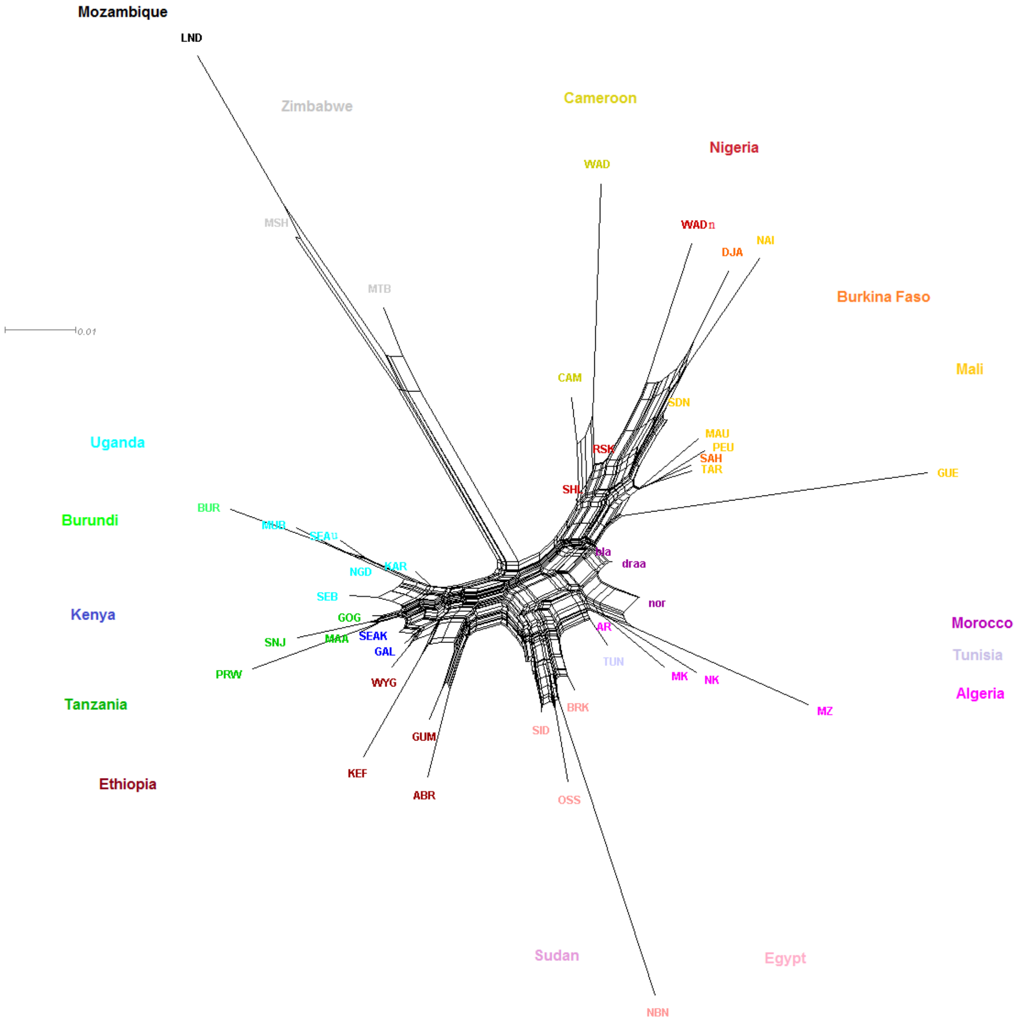

Supplementary Figure 2

a)

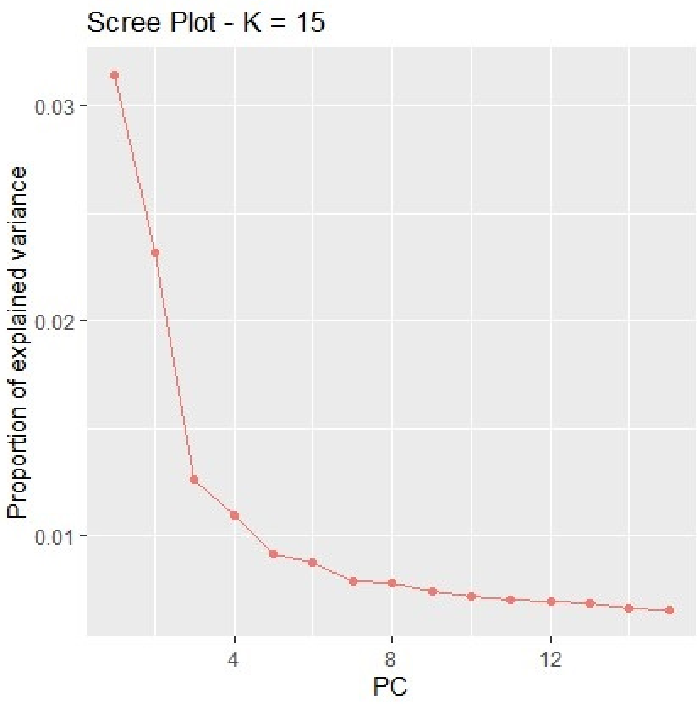

b)

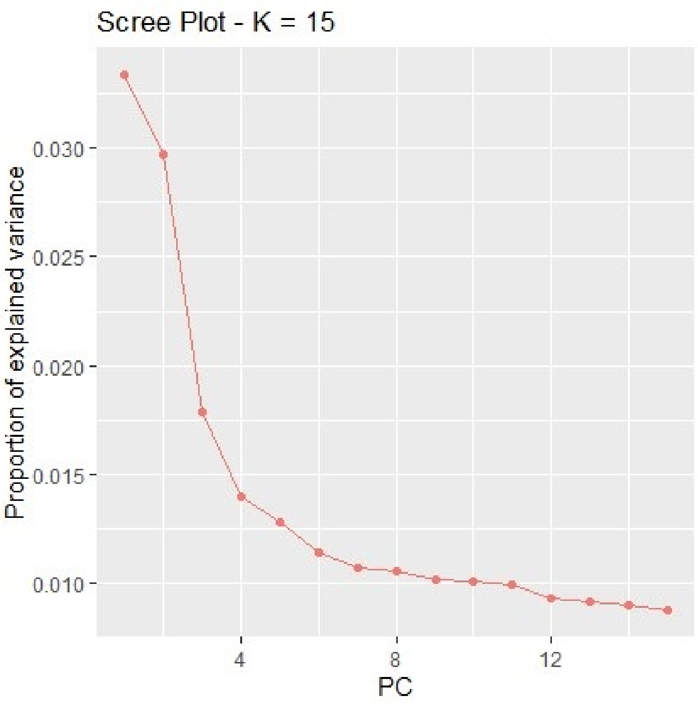

Supplementary Figure 3

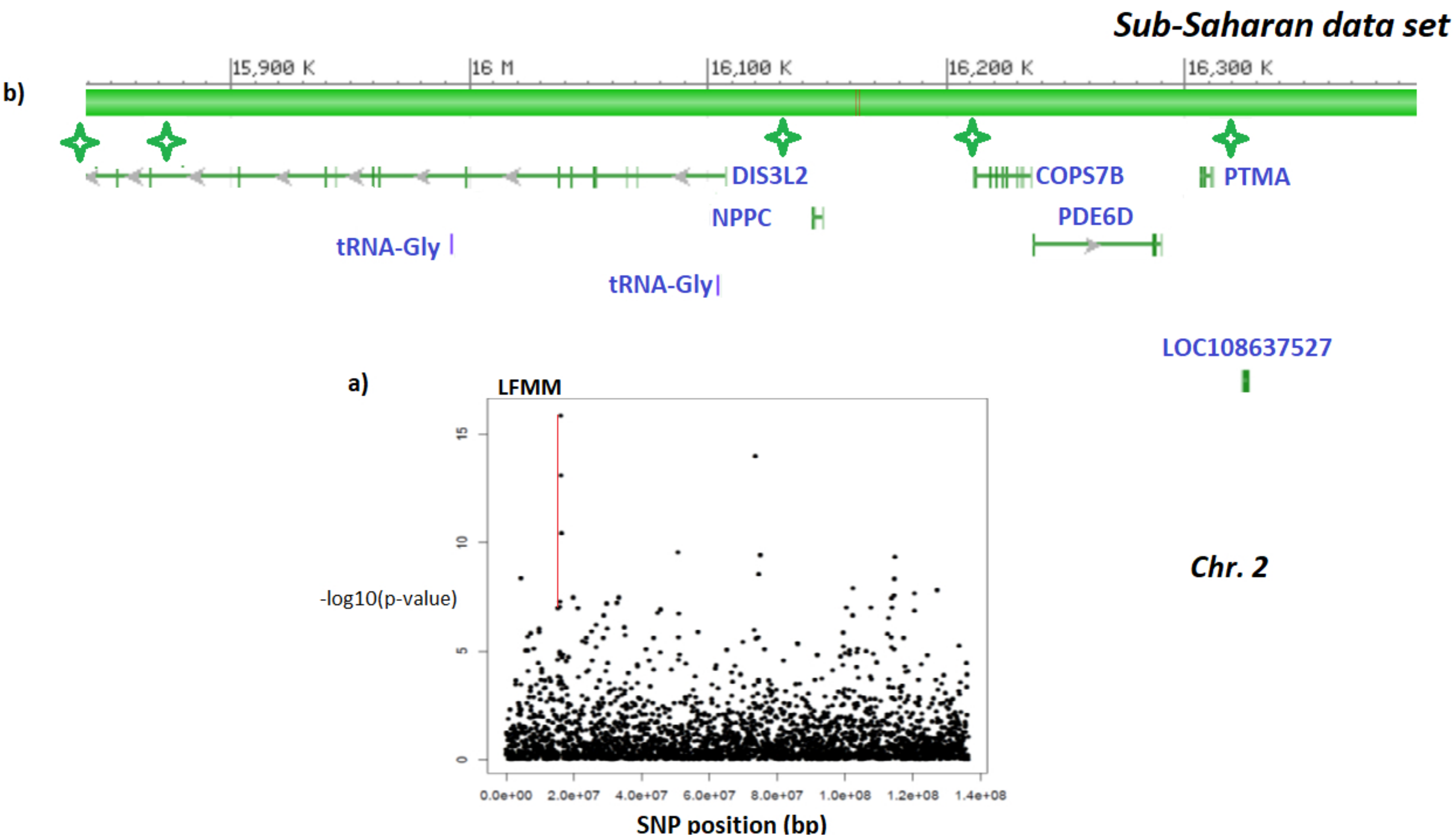

b)

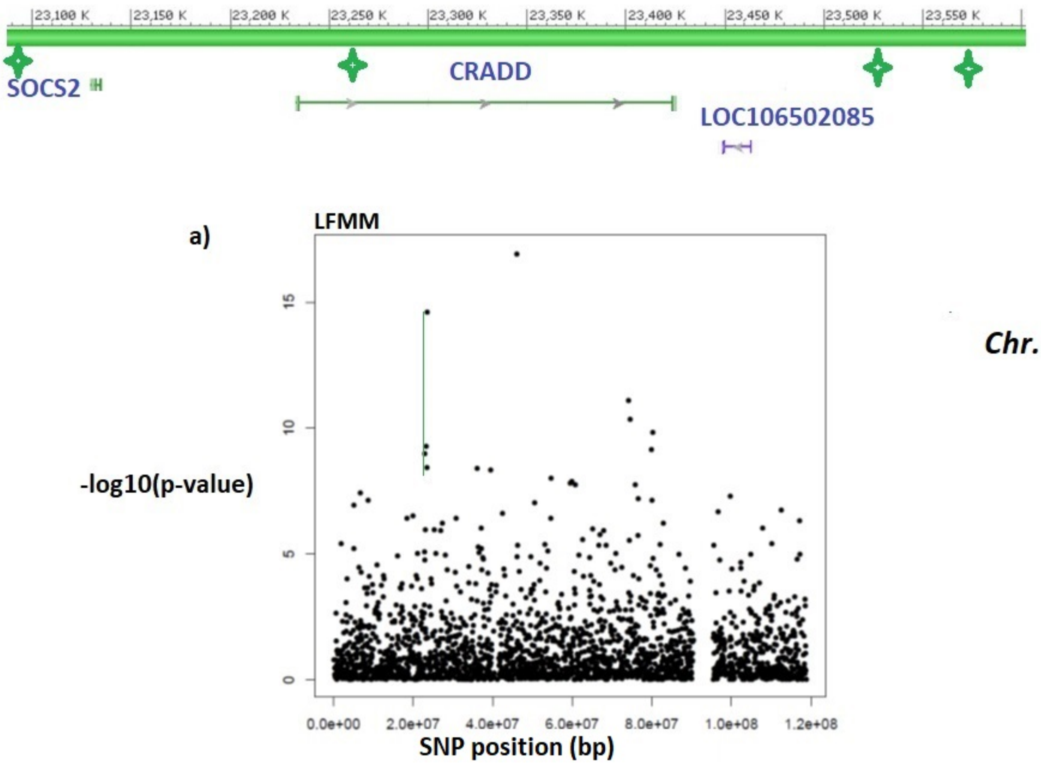
23,100 K
23,150 K
23,200 K
23,250 K
23,300 K
23,350 K
23,400 K
23,450 K
23,500 K
23,550 K
SOCS2
CRADD
LOC106502085
a)
LFMM
-log10(p-value)
0
5
10
15
0.0e+00
2.0e+07
4.0e+07
6.0e+07
8.0e+07
1.0e+08
1.2e+08
SNP position (bp)

Chr. 5

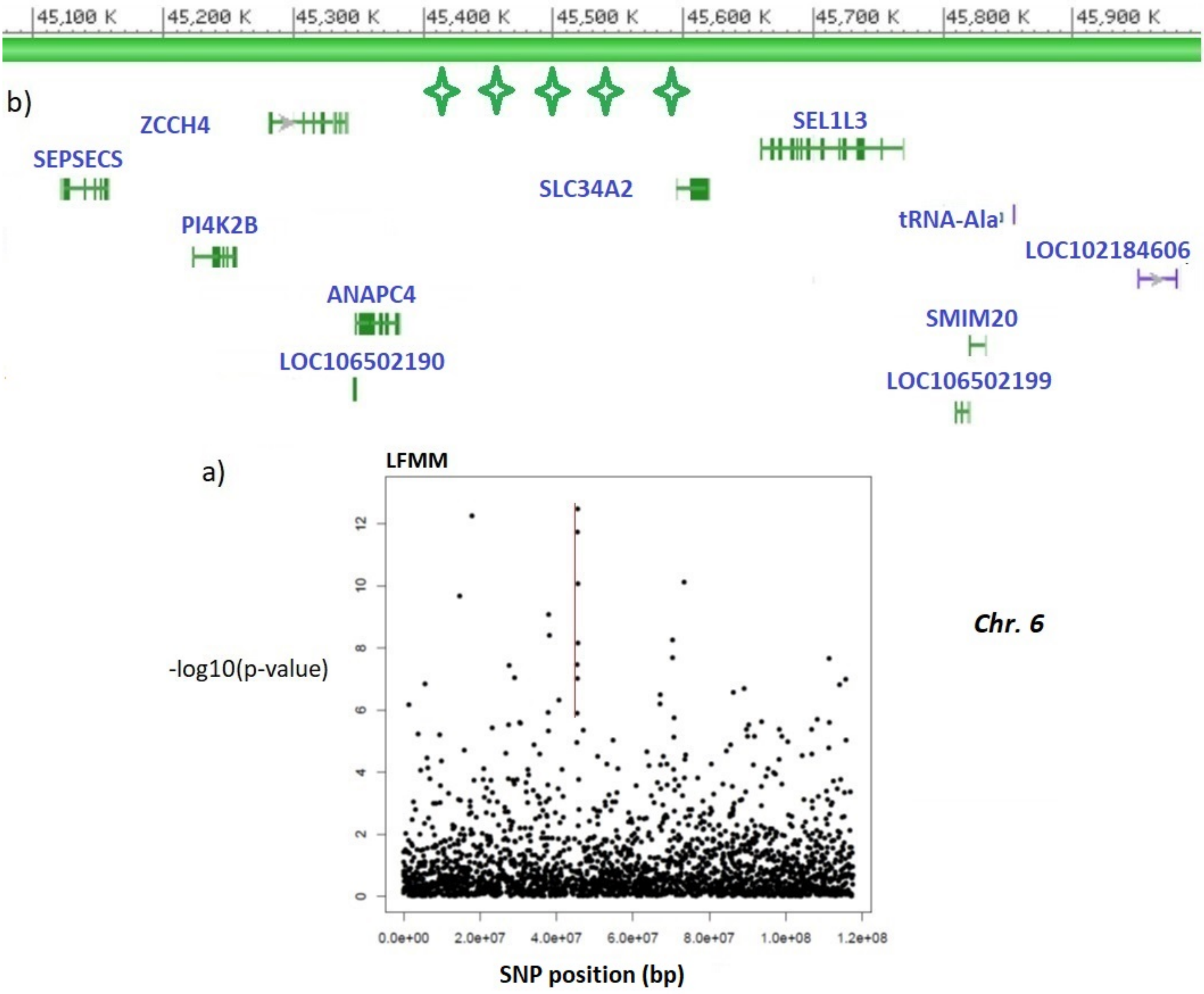
45,100 K
45,200 K
45,300 K
45,400 K
45,500 K
45,600 K
45,700 K
45,800 K
45,900 K
b)
ZCCH4
SEPSECS
PI4K2B
ANAPC4
LOC106502190
SLC34A2
SEL1L3
tRNA-Ala
LOC102184606
SMIM20
LOC106502199
a)
LFMM
-log10(p-value)
SNP position (bp)
Chr. 6

b)

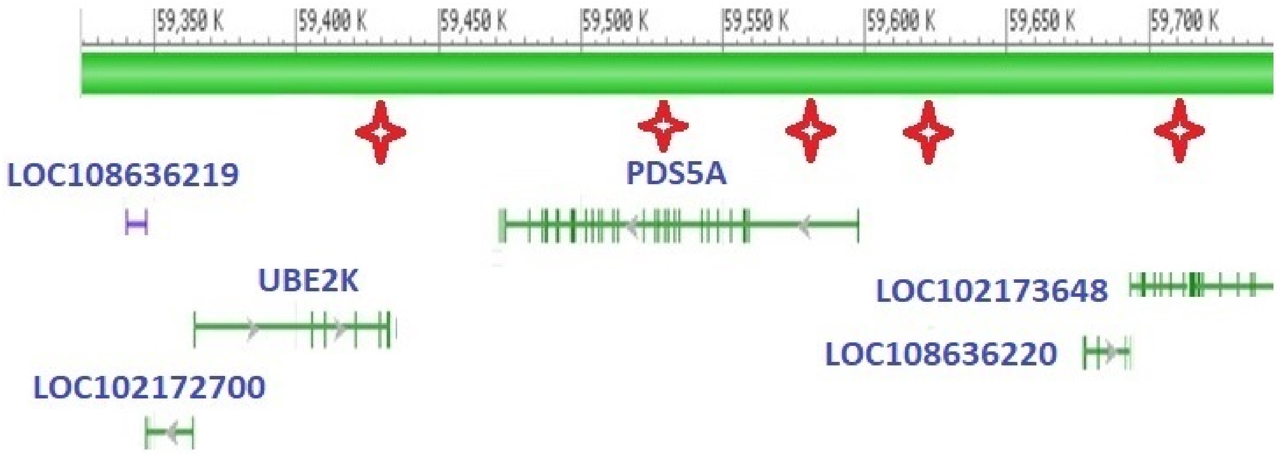
59,350 K
59,400 K
59,450 K
59,500 K
59,550 K
59,600 K
59,650 K
59,700 K
LOC108636219
PDS5A
UBE2K
LOC102173648
LOC108636220
LOC102172700

a)

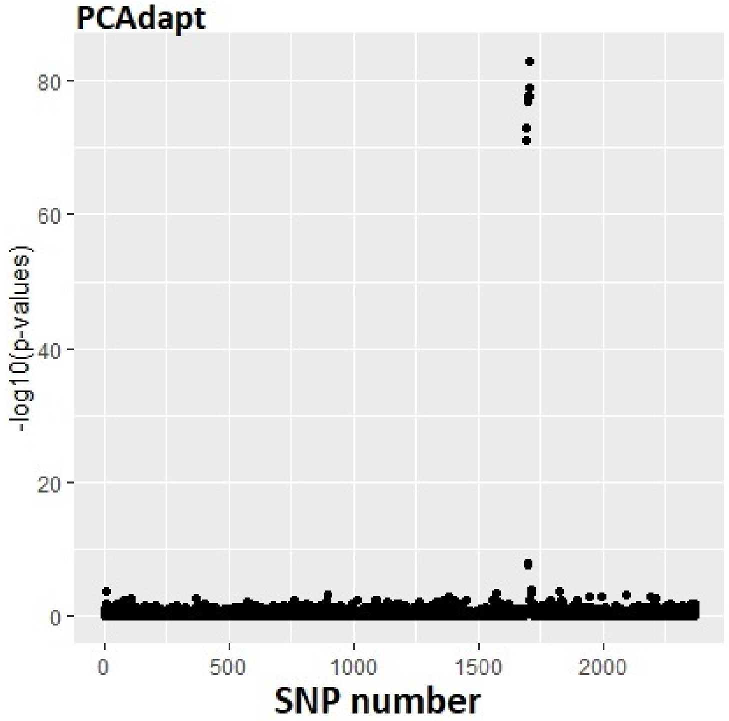
PCAdapt
-log10(p-values)
0
20
40
60
80
0
500
1000
1500
2000
SNP number

Chr. 6

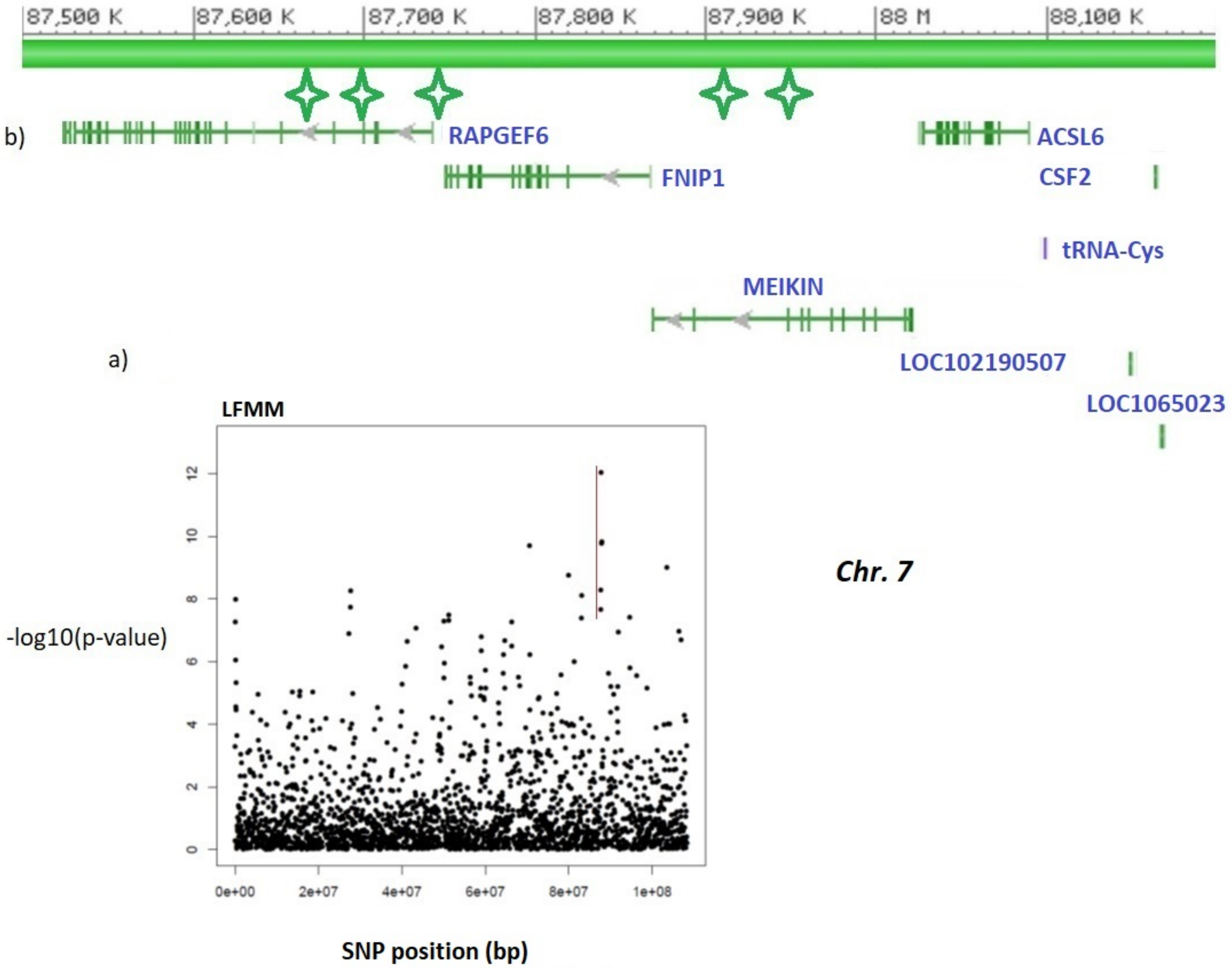
87,500 K
87,600 K
87,700 K
87,800 K
87,900 K
88 M
88,100 K
b)
RAPGEF6
ACSL6
FNIP1
CSF2
tRNA-Cys
MEIKIN
a)
LOC102190507
LOC1065023
LFMM
Chr. 7
-log10(p-value)
0e+00
2e+07
4e+07
6e+07
8e+07
1e+08
SNP position (bp)

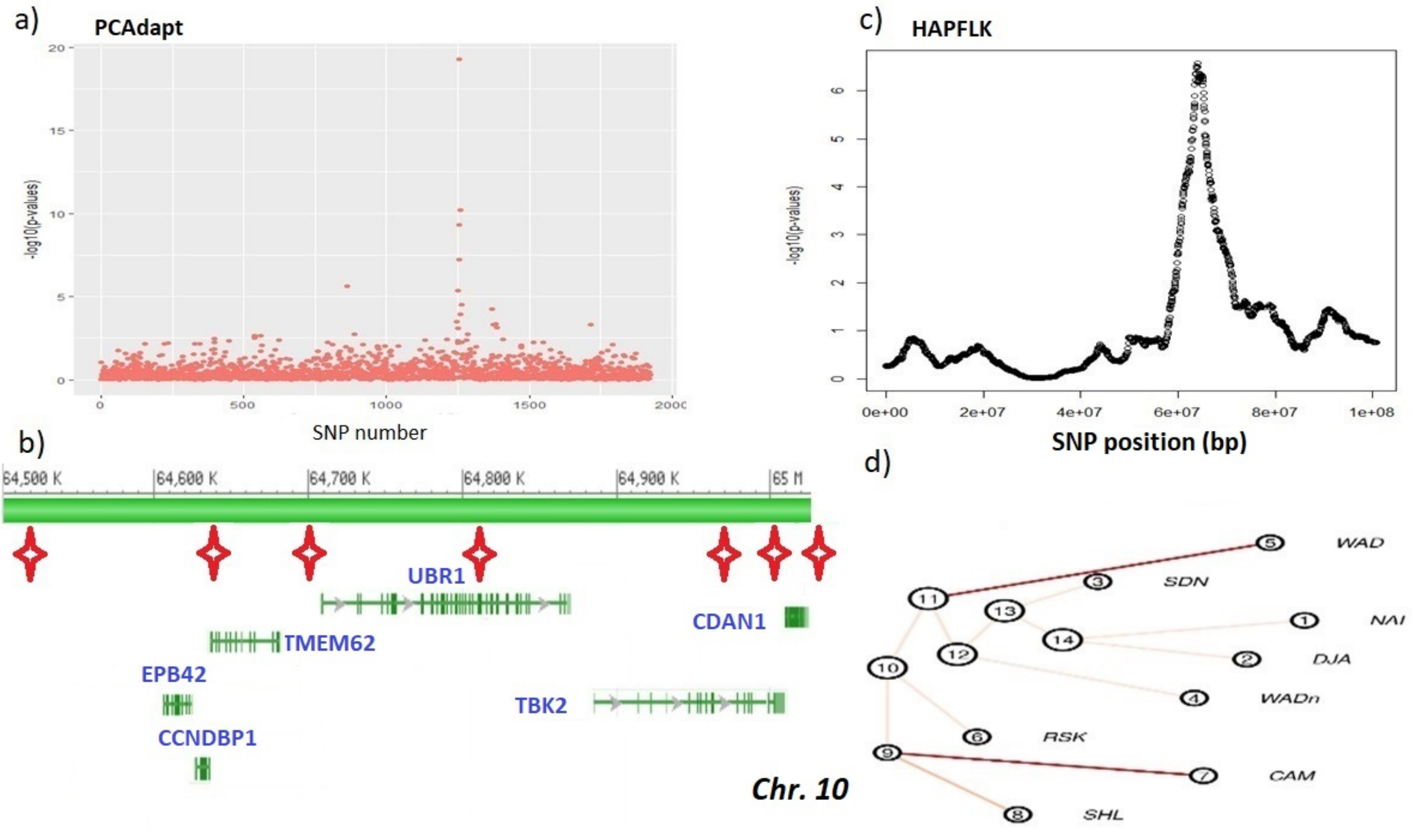

a)
PCAdapt
-log10(p-values)
SNP number
b)
64,500 K
64,600 K
64,700 K
64,800 K
64,900 K
65 M
UBR1
TMEM62
EPB42
CCNDBP1
CDAN1
TBK2
c)
HAPFLK
-log10(p-values)
SNP position (bp)
d)
WAD
SDN
NAI
DJA
WADn
RSK
CAM
SHL
Chr. 10

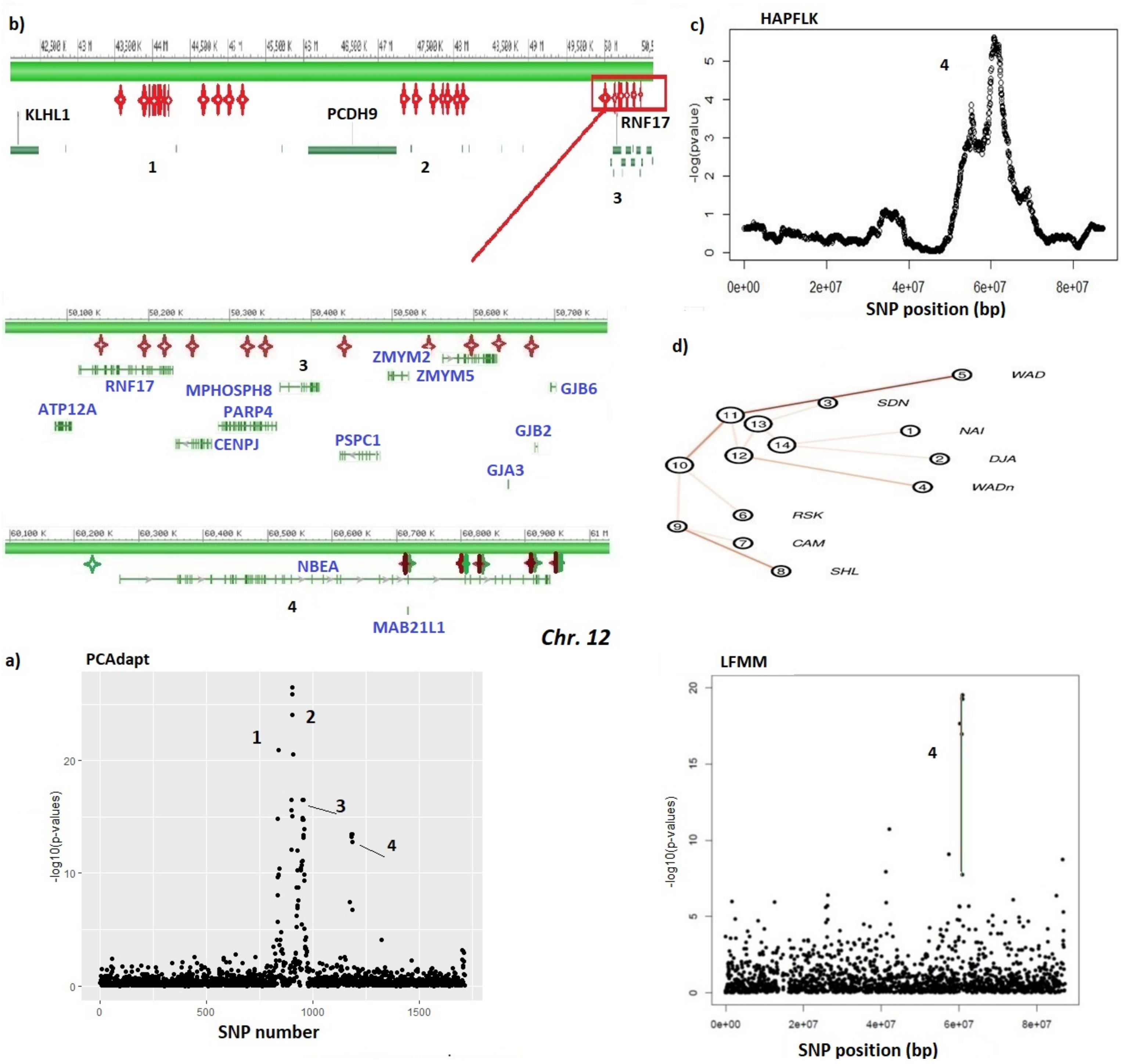
b)
KLHL1
PCDH9
RNF17
1
2
3
RNF17
MPHOSPH8
ATP12A
PARP4
CENPJ
PSPC1
ZMYM2
ZMYM5
GJB6
GJB2
GJA3
NBEA
4
MAB21L1
Chr. 12
c)
HAPFLK
-log(pvalue)
SNP position (bp)
d)
WAD
SDN
NAI
DJA
WADn
RSK
CAM
SHL
a)
PCAdapt
-log10(p-values)
SNP number
LFMM
-log10(p-values)
SNP position (bp)

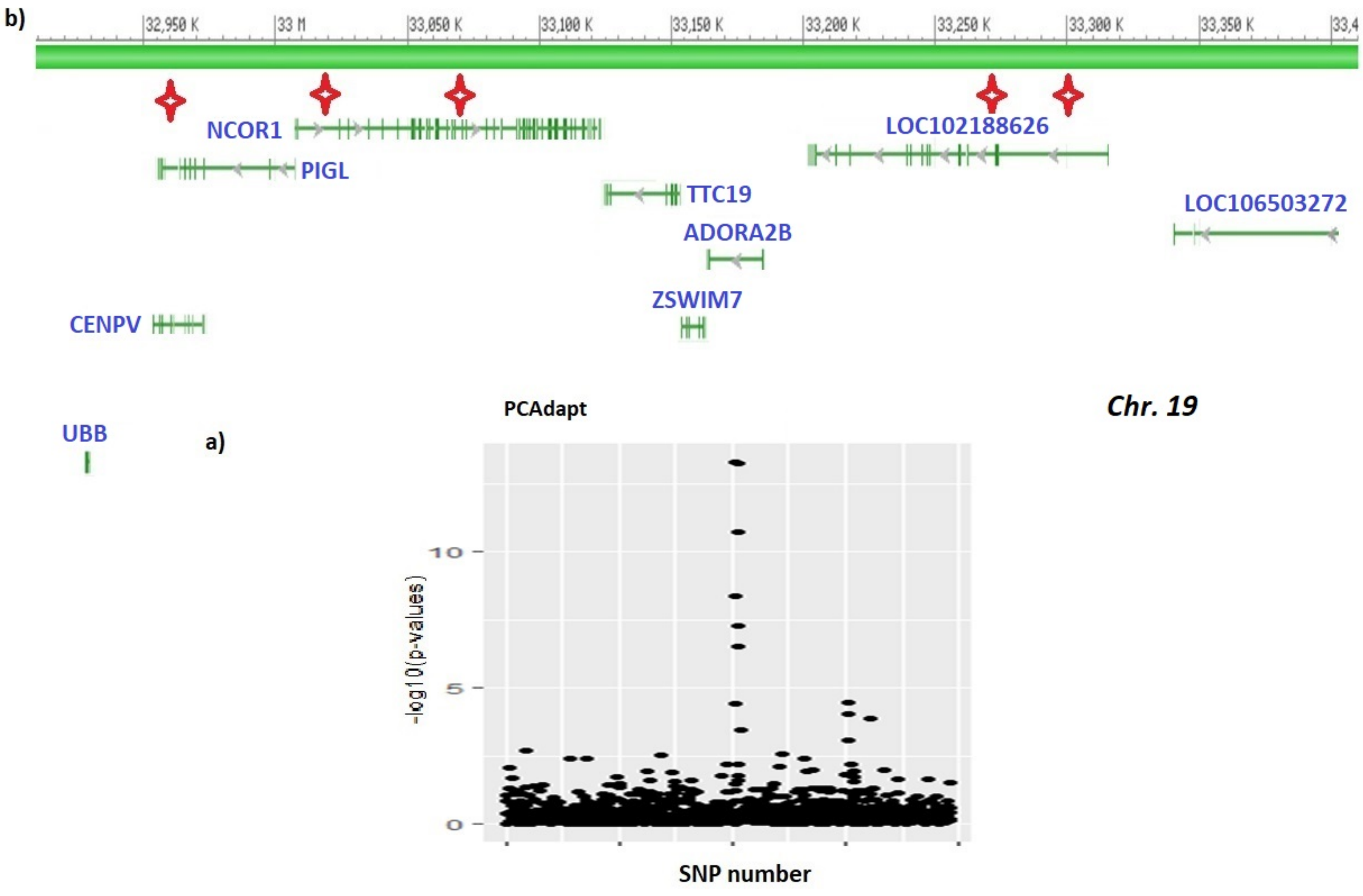
b)
32,950 K
33 M
33,050 K
33,100 K
33,150 K
33,200 K
33,250 K
33,300 K
33,350 K
NCOR1
PIGL
LOC102188626
TTC19
ADORA2B
LOC106503272
ZSWIM7
CENPV
UBB
a)
PCAdapt
Chr. 19
-log10(p-values)
10
5
0
SNP number

b)

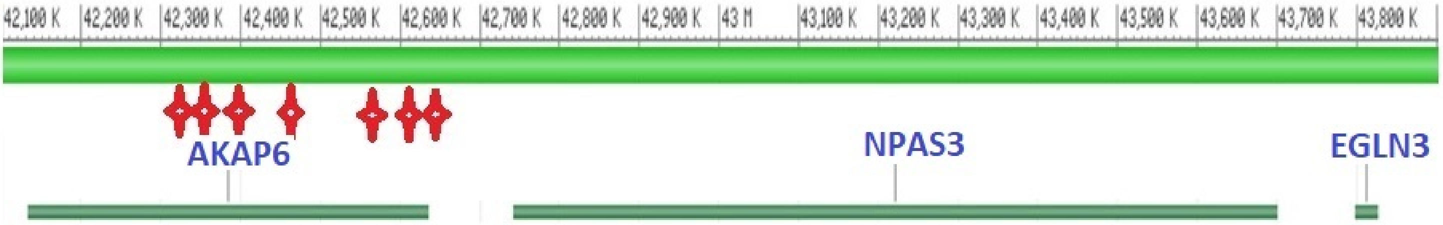
42,100 K
42,200 K
42,300 K
42,400 K
42,500 K
42,600 K
42,700 K
42,800 K
42,900 K
43 M
43,100 K
43,200 K
43,300 K
43,400 K
43,500 K
43,600 K
43,700 K
43,800 K
AKAP6
NPAS3
EGLN3

a)

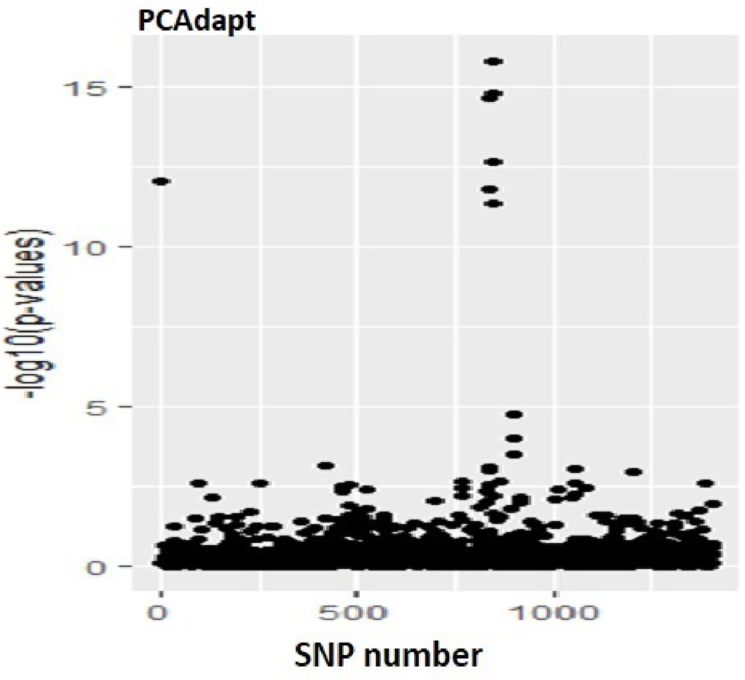
PCAdapt
-log10(p-values)
15
10
5
0
0
500
1000
SNP number

Chr. 21

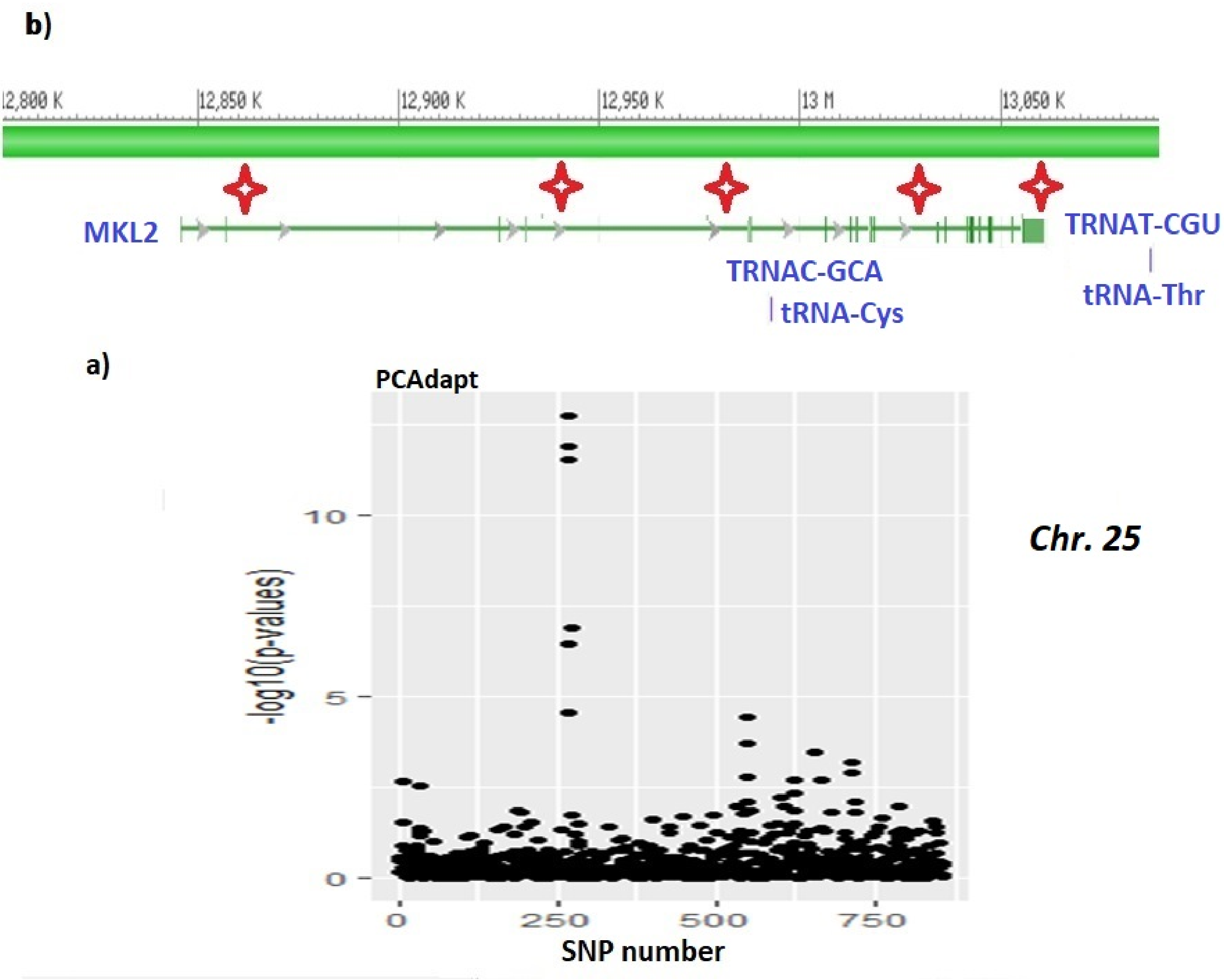
b)
12,800 K
12,850 K
12,900 K
12,950 K
13 M
13,050 K
MKL2
TRNAT-CGU
tRNA-Thr
TRNAC-GCA
tRNA-Cys
a)
PCAdapt
-log10(p-values)
10
5
0
0
250
500
750
SNP number
Chr. 25

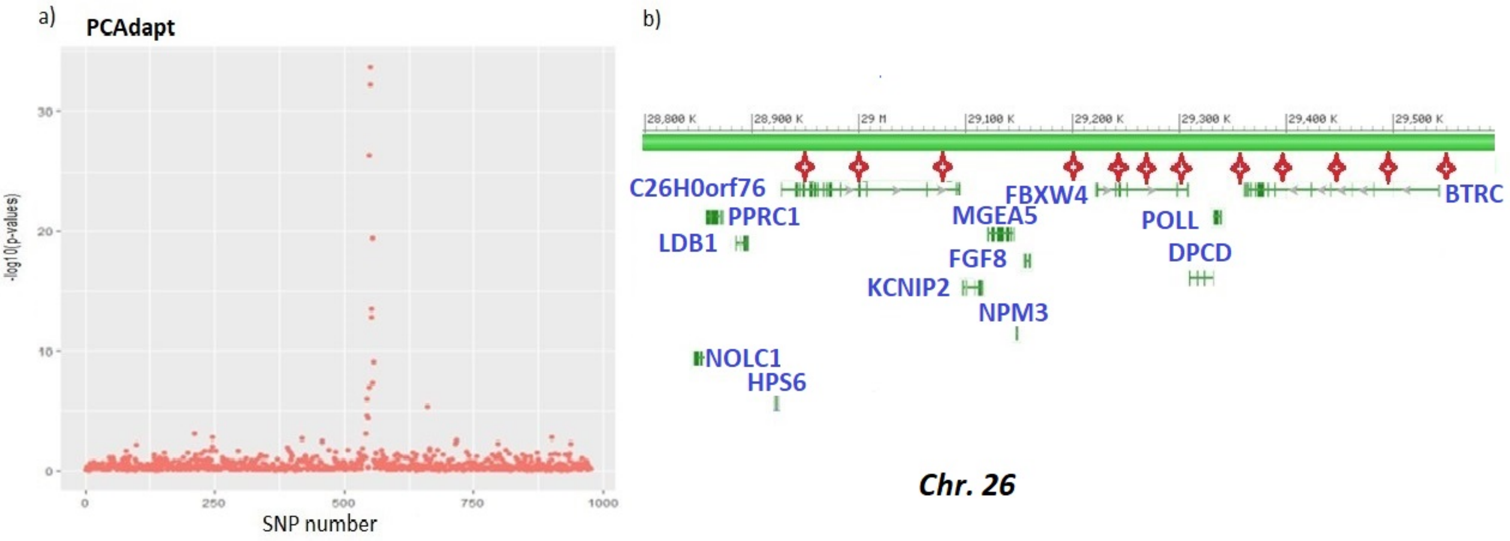
a)
PCAdapt
-log10(p-values)
30
20
10
0
0
250
500
750
1000
SNP number
b)
28,800 K
28,900 K
29 M
29,100 K
29,200 K
29,300 K
29,400 K
29,500 K
C26H0orf76
PPRC1
LDB1
MGEA5
FBXW4
POLL
DPCD
FGF8
KCNIP2
NPM3
NOLC1
HPS6
BTRC
Chr. 26

Supplementary Figure 4

Subsaharan data set

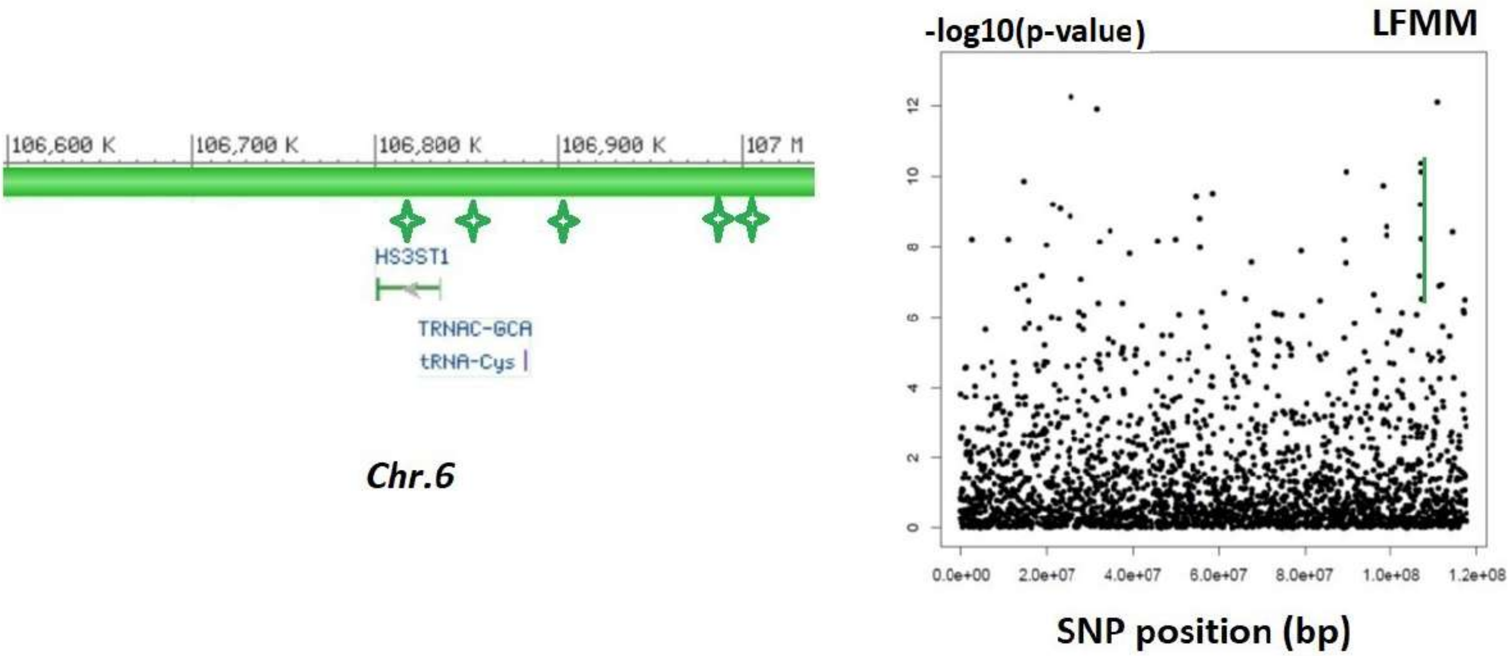

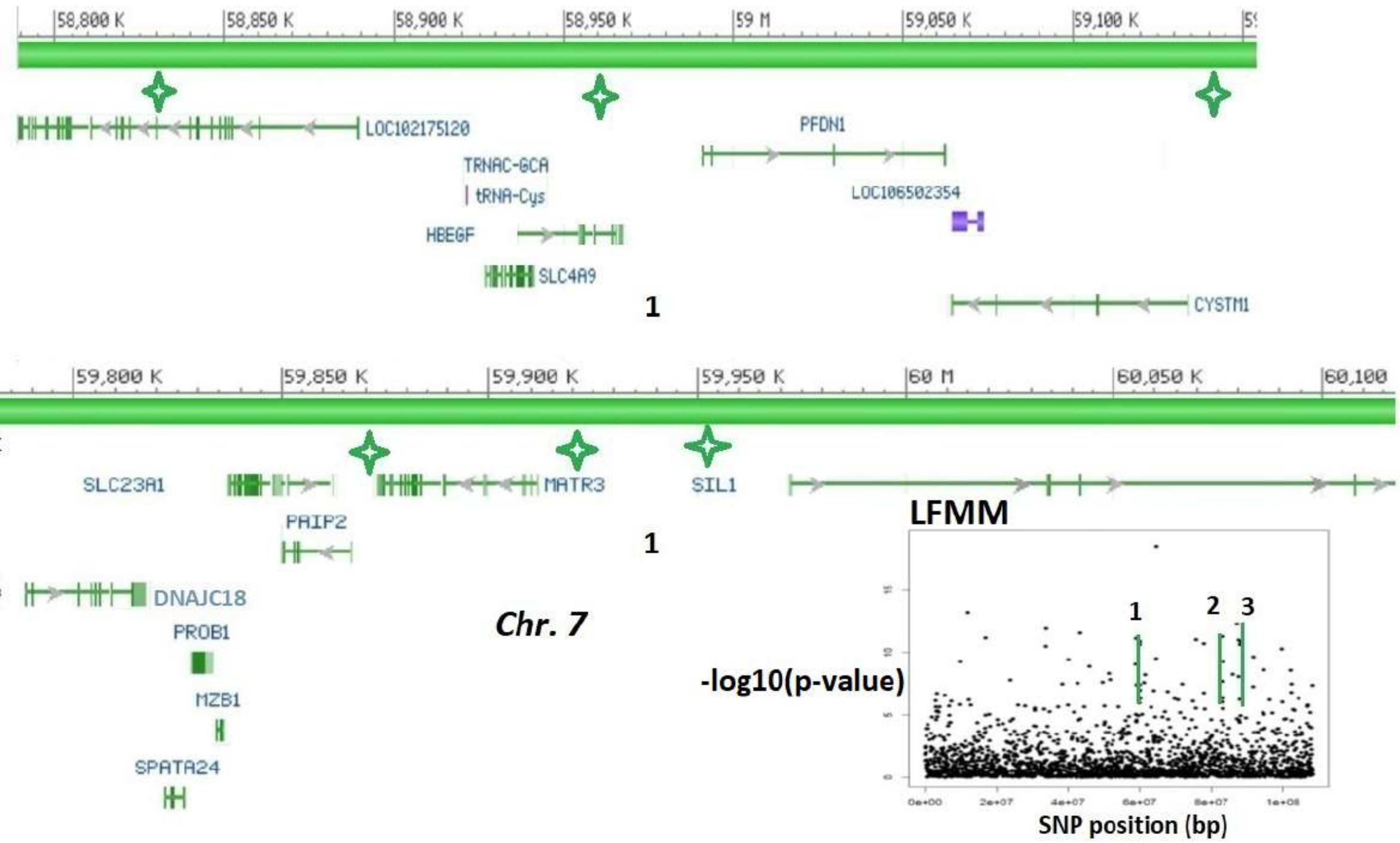
58,800 K
58,850 K
58,900 K
58,950 K
59 M
59,050 K
59,100 K
LOC102175120
PFDN1
TRNAC-GCA
tRNA-Cys
LOC106502354
HBEGF
SLC4A9
1
CYSTM1
59,800 K
59,850 K
59,900 K
59,950 K
60 M
60,050 K
60,100
SLC23A1
MATR3
SIL1
PAIP2
1
LFMM
DNAJC18
PROB1
Chr. 7
MZB1
SPATA24
1
2
3
-log10(p-value)
SNP position (bp)

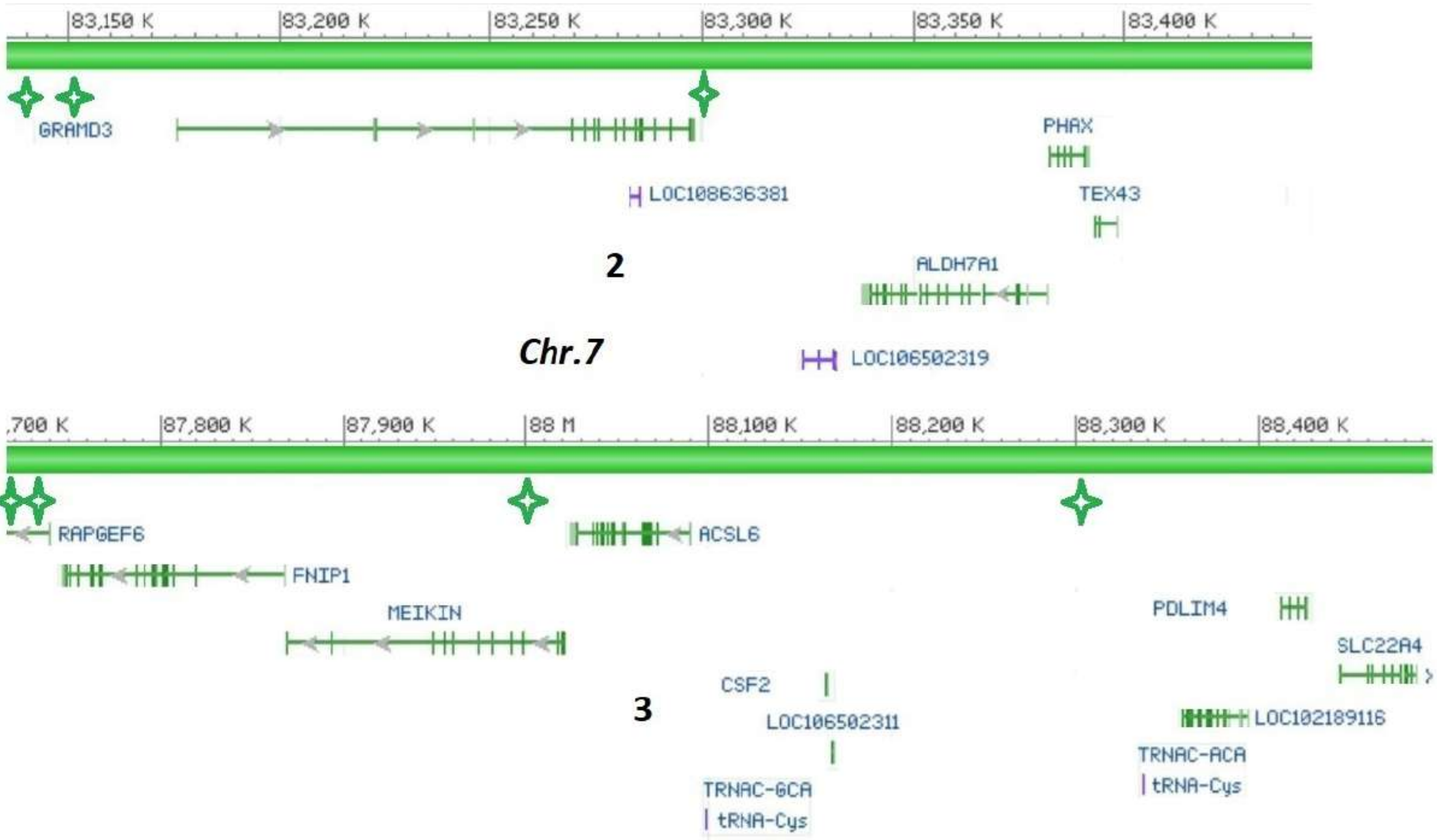

83,150 K
83,200 K
83,250 K
83,300 K
83,350 K
83,400 K
GRAMD3
PHAX
LOC108636381
TEX43
2
ALDH7A1
Chr.7
LOC106502319
,700 K
87,800 K
87,900 K
88 M
88,100 K
88,200 K
88,300 K
88,400 K
RAPGEF6
ACSL6
FNIP1
MEIKIN
PDLIM4
SLC22A4
CSF2
3
LOC106502311
LOC102189116
TRNAC-ACA
tRNA-Cys
TRNAC-GCA
tRNA-Cys

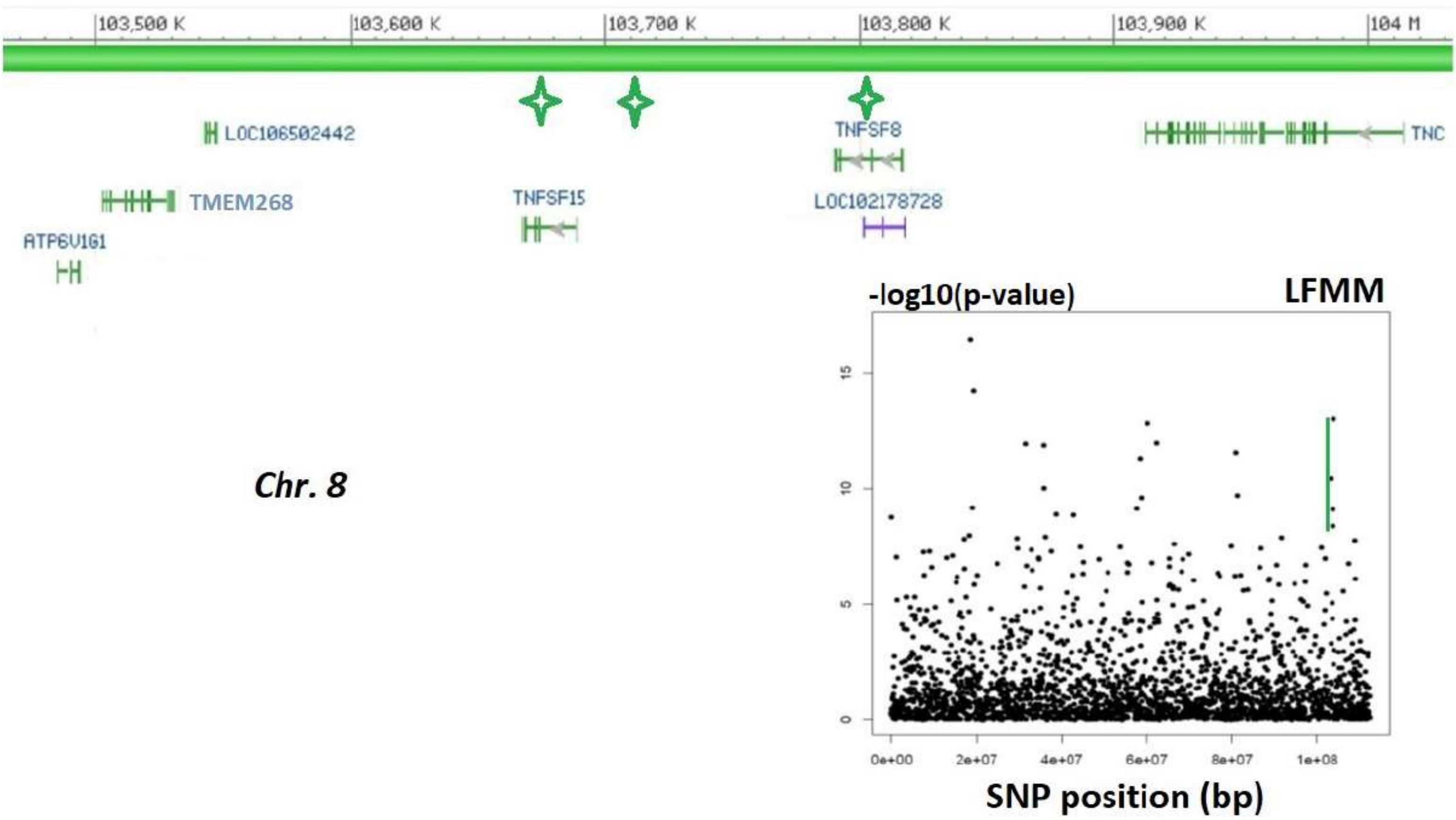

103,500 K
103,600 K
103,700 K
103,800 K
103,900 K
104 M
LOC106502442
TMEM268
ATP6V1G1
TNFSF15
TNFSF8
LOC102178728
TNC
Chr. 8
-log10(p-value)
LFMM
0
5
10
15
0e+00
2e+07
4e+07
6e+07
8e+07
1e+08
SNP position (bp)

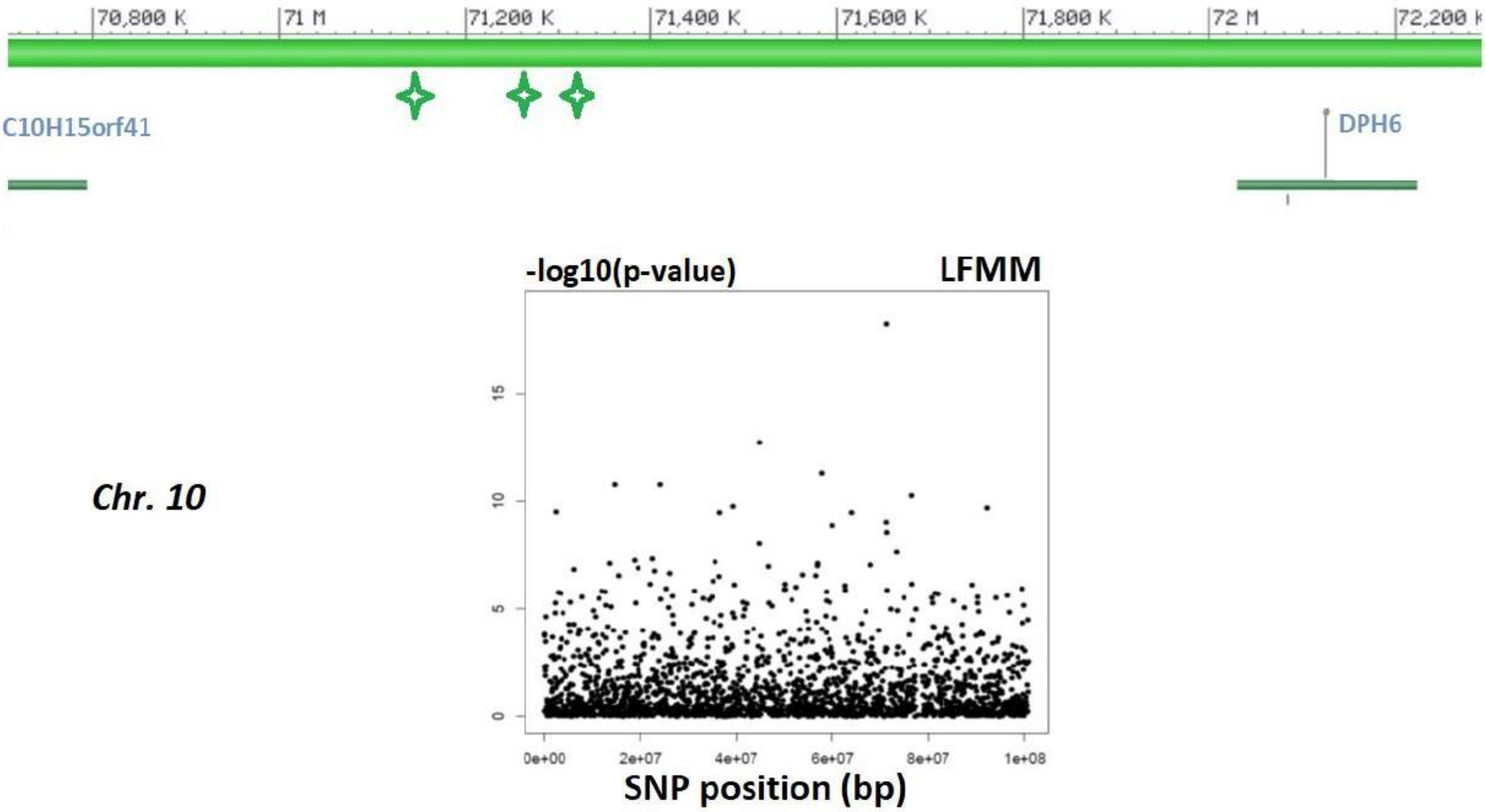
70,800 K
71 M
71,200 K
71,400 K
71,600 K
71,800 K
72 M
C10H15orf41
DPH6
-log10(p-value)
LFMM
15
10
5
0
0e+00
2e+07
4e+07
6e+07
8e+07
1e+08
SNP position (bp)

Chr. 10

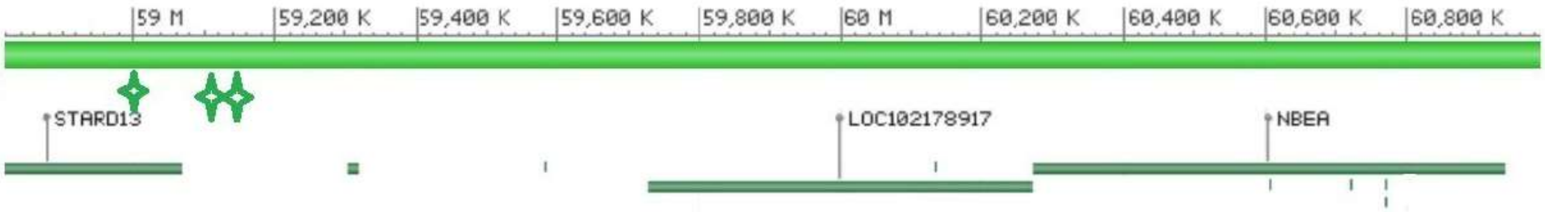
59 M
59,200 K
59,400 K
59,600 K
59,800 K
60 M
60,200 K
60,400 K
60,600 K
60,800 K
STARD13
LOC102178917
NBEA

Chr. 12

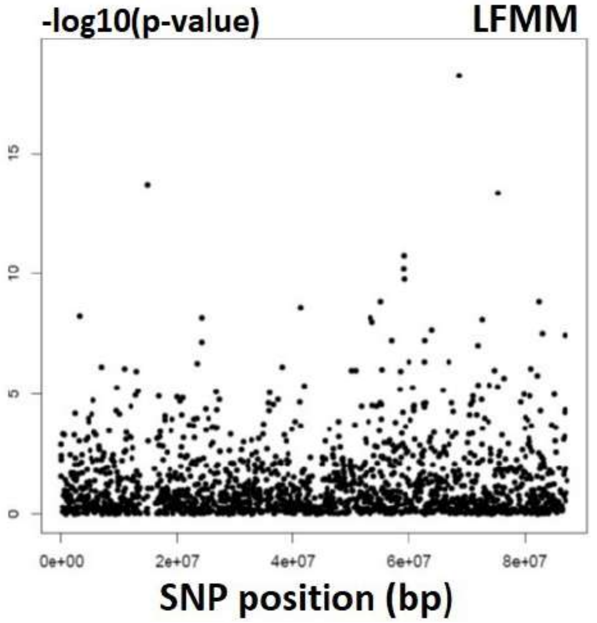
-log10(p-value)
LFMM
0
5
10
15
0e+00
2e+07
4e+07
6e+07
8e+07
SNP position (bp)

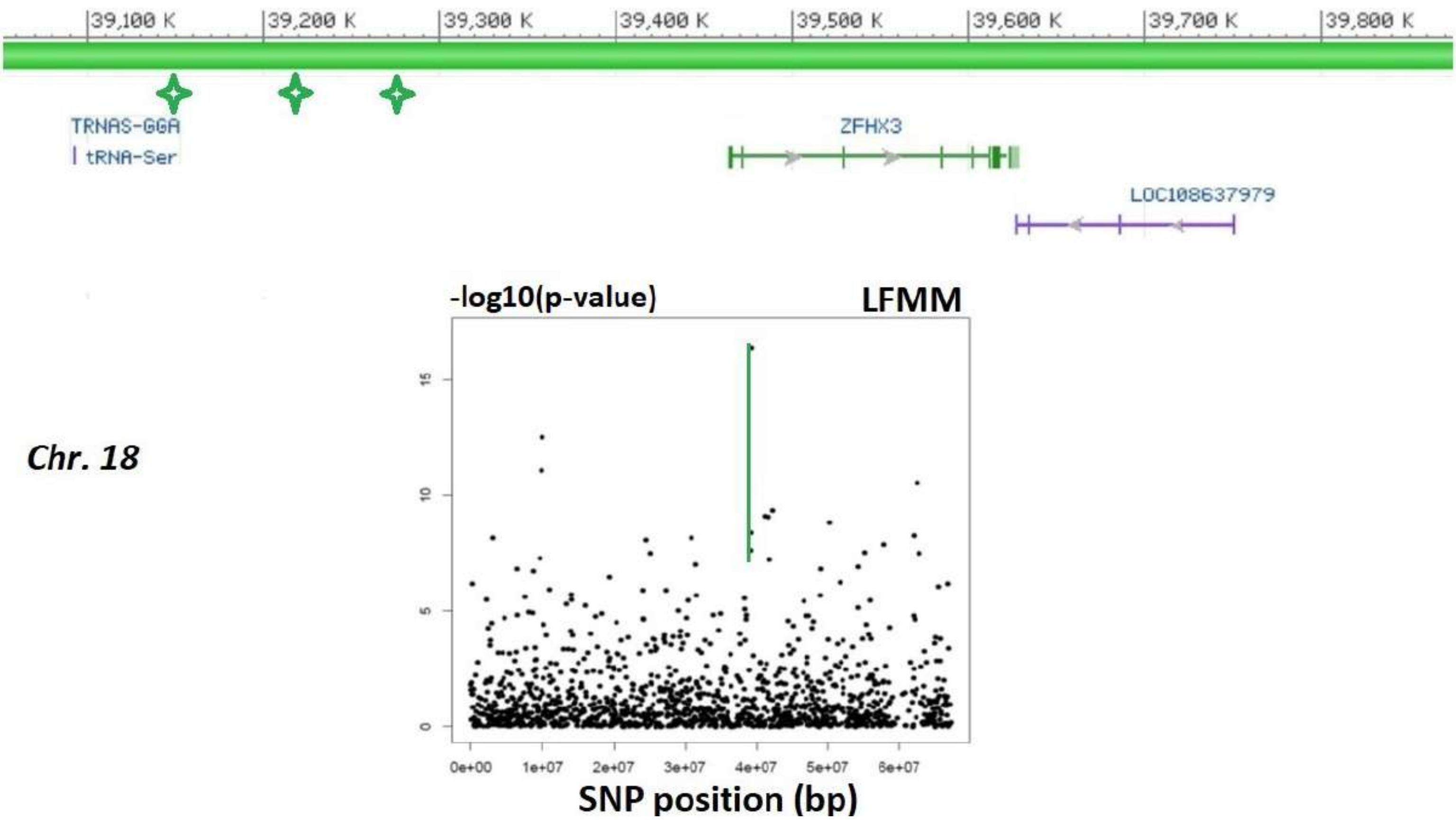
39,100 K
39,200 K
39,300 K
39,400 K
39,500 K
39,600 K
39,700 K
39,800 K
TRNAS-GGA
tRNA-Ser
ZFHX3
LOC108637979
Chr. 18
-log10(p-value)
LFMM
0
5
10
15
0e+00
1e+07
2e+07
3e+07
4e+07
5e+07
6e+07
SNP position (bp)

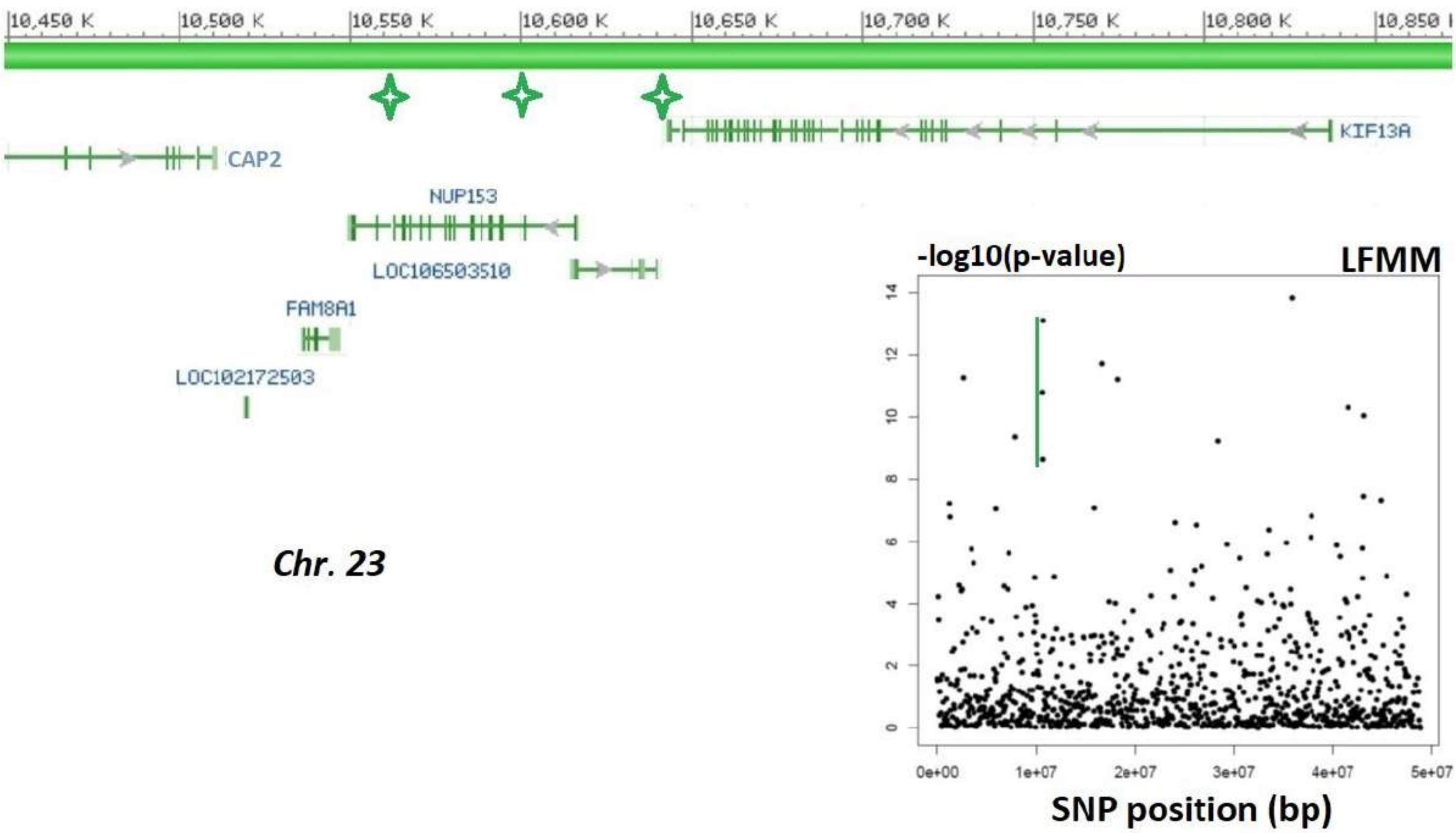

10,450 K
10,500 K
10,550 K
10,600 K
10,650 K
10,700 K
10,750 K
10,800 K
KIF13A
CAP2
NUP153
LOC106503510
FAM8A1
LOC102172503
Chr. 23
-log10(p-value)
LFMM
SNP position (bp)

Supplementary Figure 5

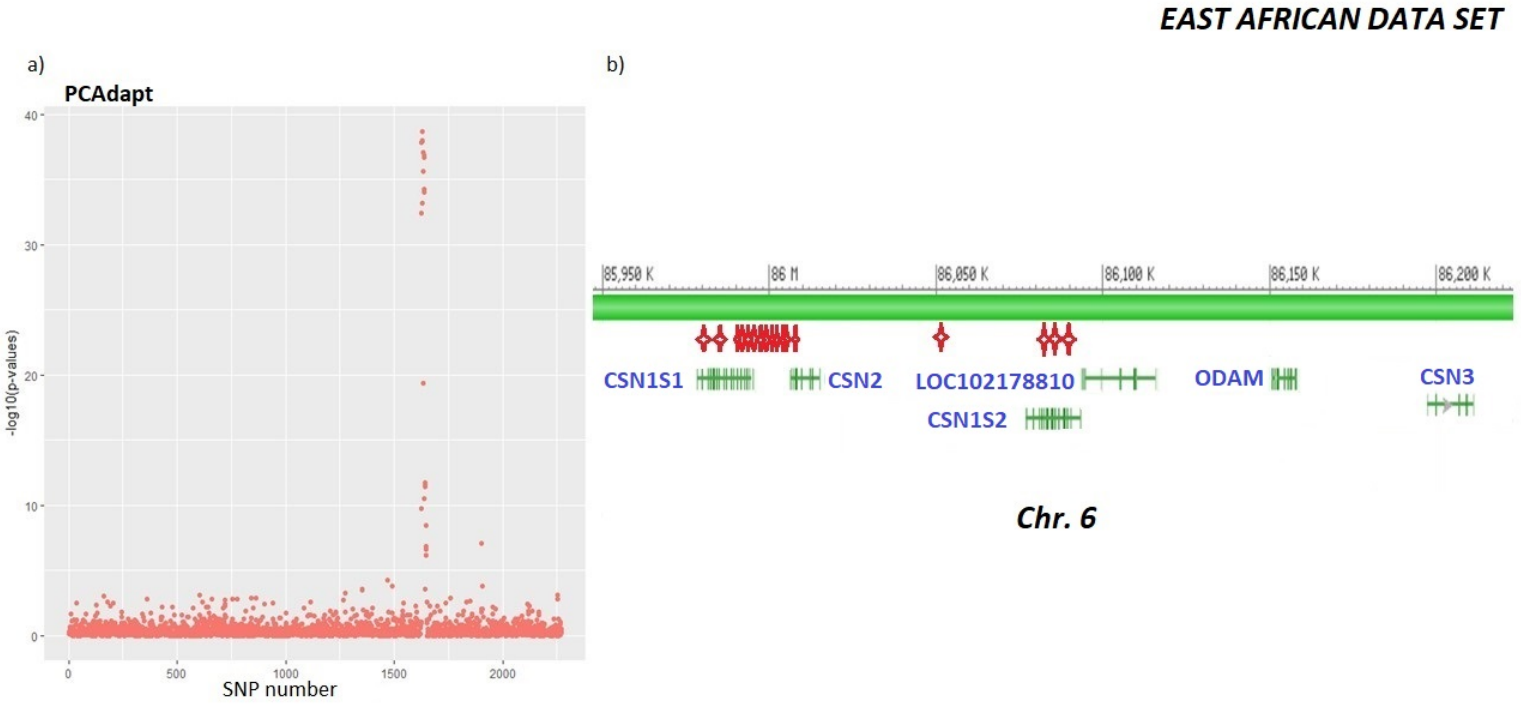

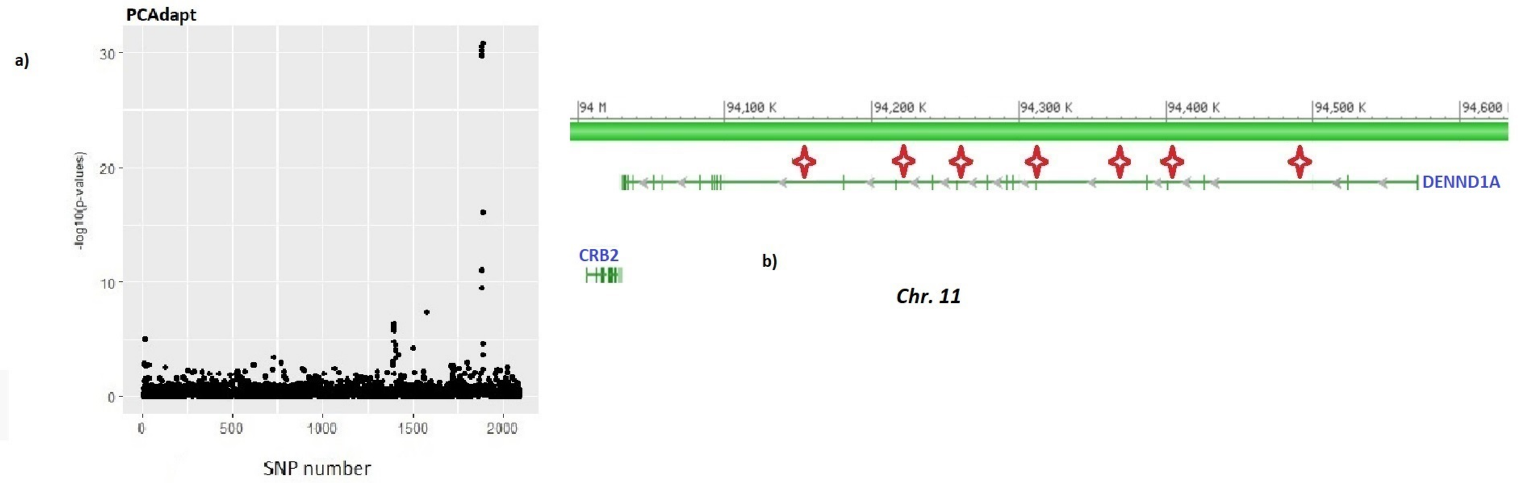
a)
PCAdapt
-log10(p-values)
SNP number
b)
DENND1A
CRB2
Chr. 11

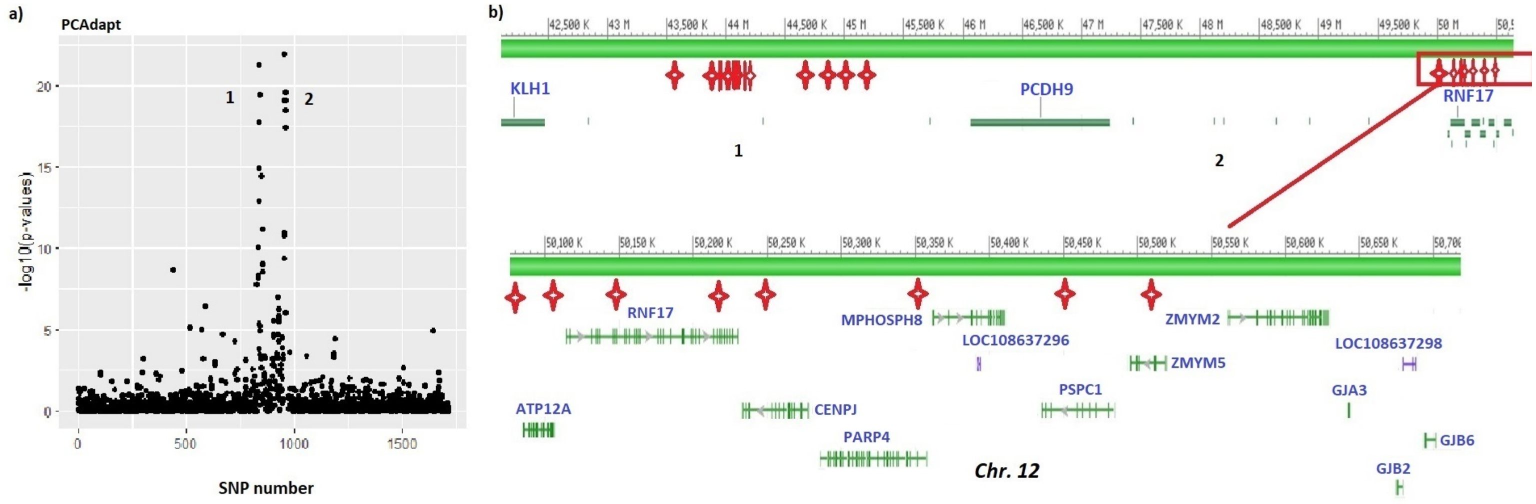
a)
PCAdapt
-log10(p-values)
SNP number
b)
KLH1
PCDH9
RNF17
MPHOSPH8
LOC108637296
ZMYM2
ZMYM5
LOC108637298
PSPC1
GJA3
ATP12A
CENPJ
PARP4
GJB6
GJB2
Chr. 12

a)

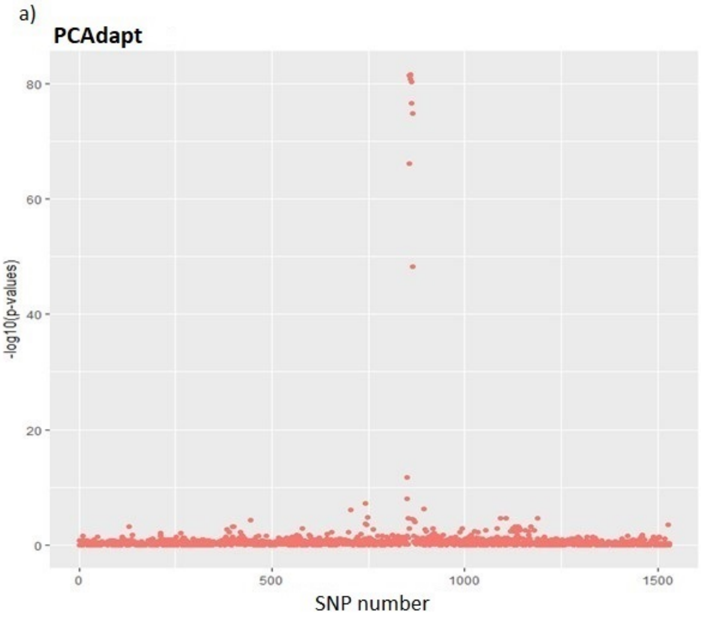

PCAdapt
80
60
40
20
0
-log10(p-values)
0
500
1000
1500
SNP number

b)

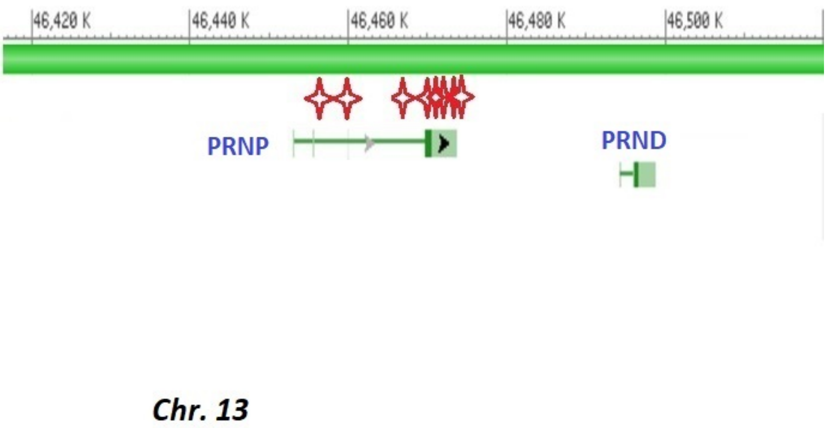

46,420 K
46,440 K
46,460 K
46,480 K
46,500 K
PRNP
PRND
Chr. 13

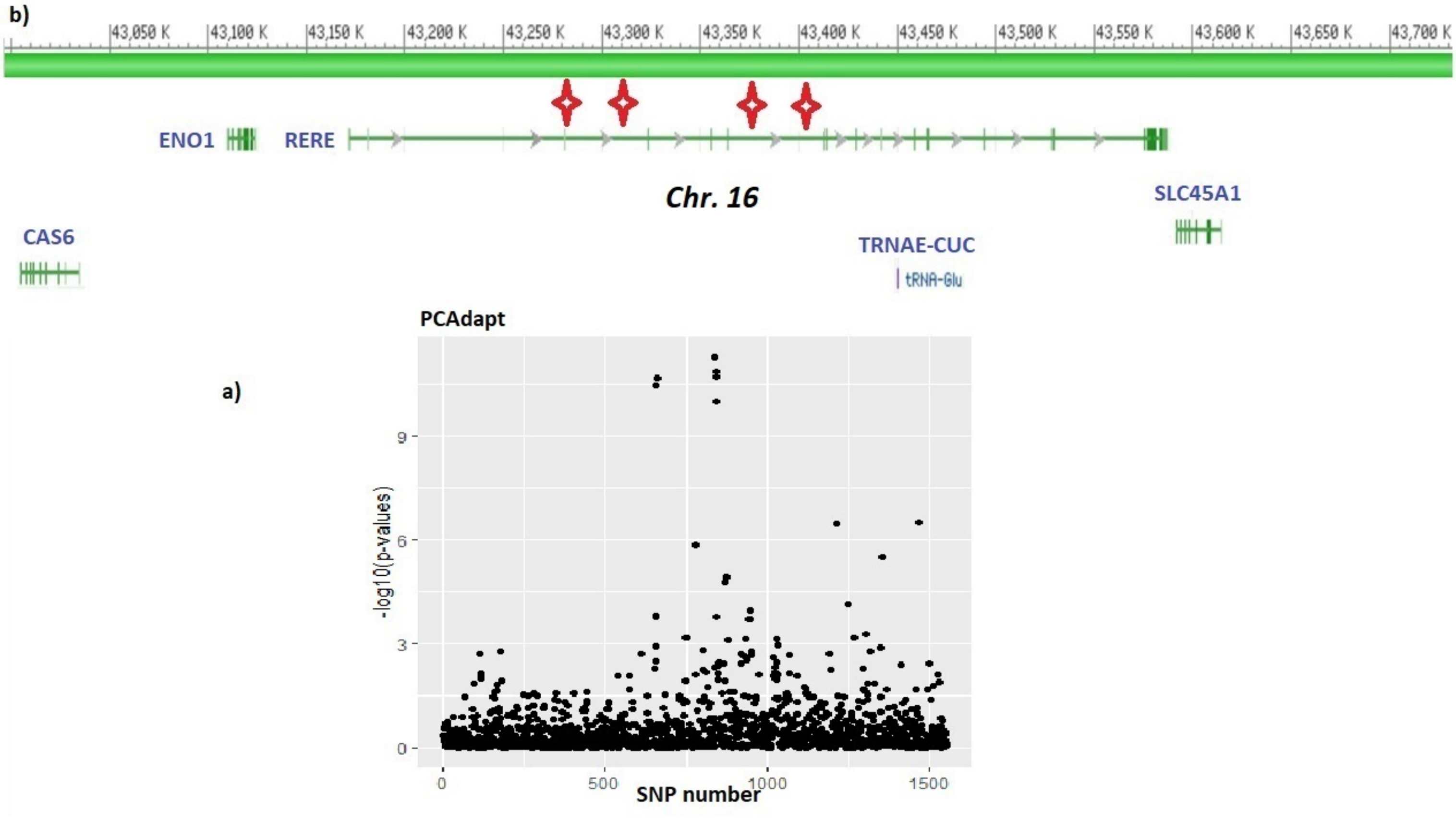

b)
43,050 K
43,100 K
43,150 K
43,200 K
43,250 K
43,300 K
43,350 K
43,400 K
43,450 K
43,500 K
43,550 K
43,600 K
43,650 K
43,700 K
ENO1
RERE
Chr. 16
SLC45A1
CAS6
TRNAE-CUC
tRNA-Glu
PCAdapt
a)
-log10(p-values)
0
3
6
9
0
500
1000
1500
SNP number

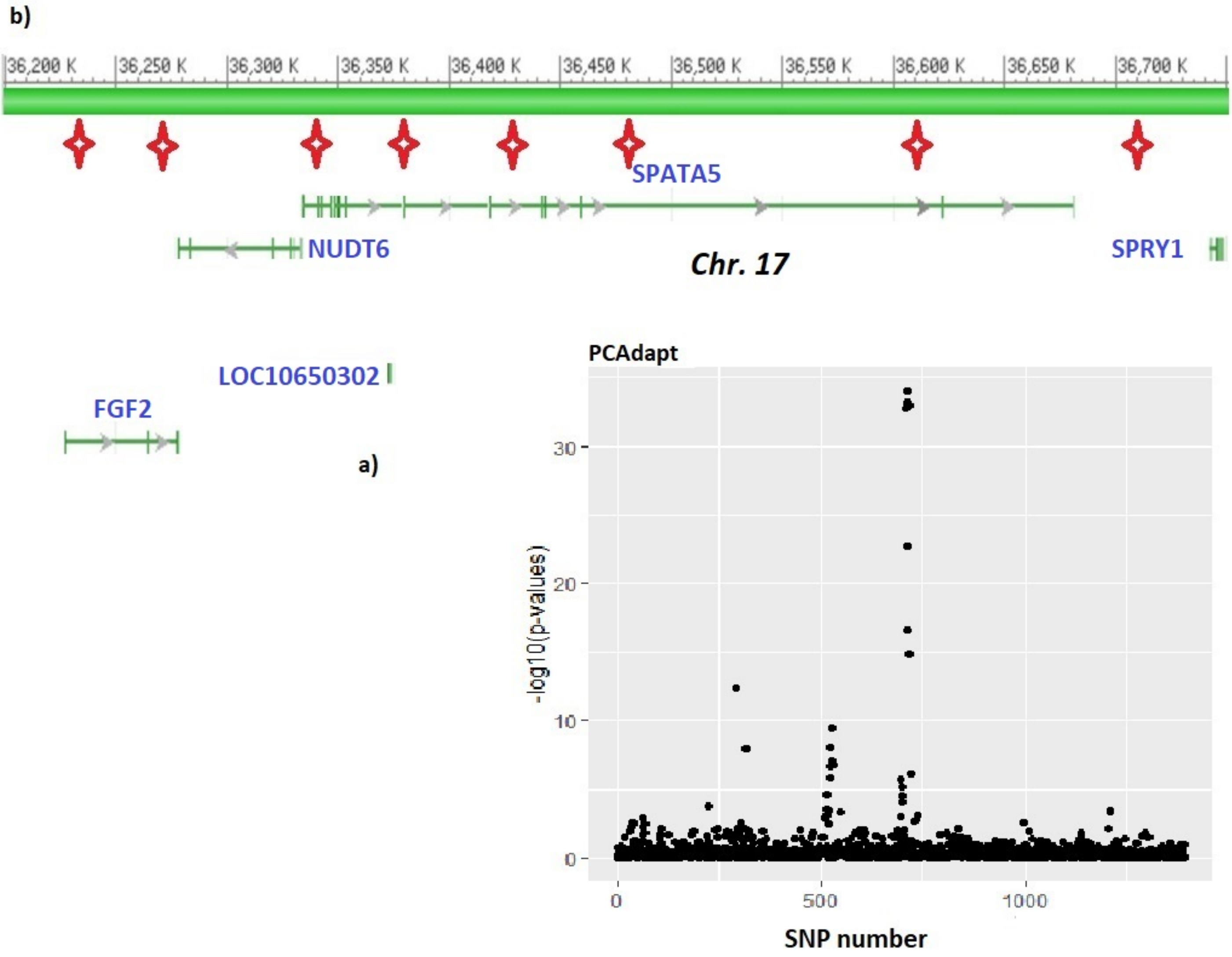
b)
36,200 K
36,250 K
36,300 K
36,350 K
36,400 K
36,450 K
36,500 K
36,550 K
36,600 K
36,650 K
36,700 K
SPATA5
NUDT6
Chr. 17
SPRY1
LOC10650302
FGF2
a)
PCAdapt
-log10(p-values)
0
10
20
30
0
500
1000
SNP number

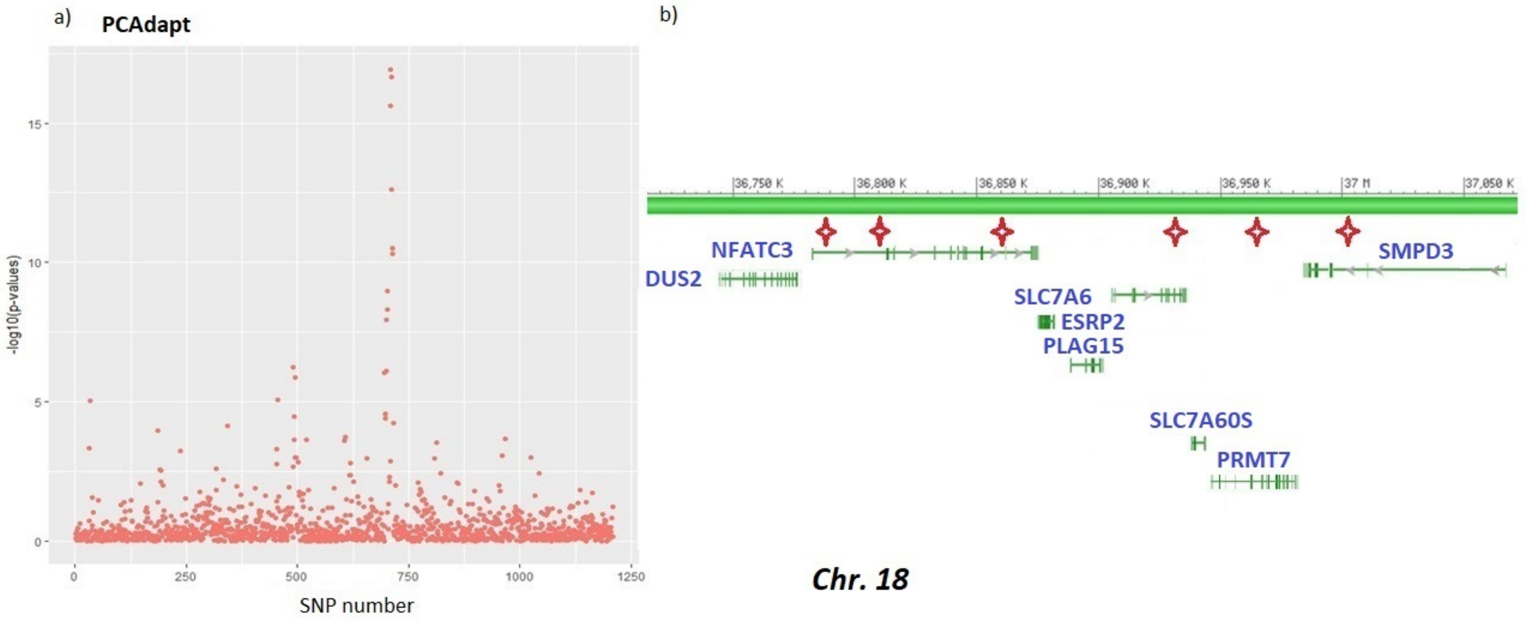

a)
PCAdapt
-log10(p-values)
SNP number
b)
NFATC3
DUS2
SLC7A6
ESRP2
PLAG15
SLC7A60S
PRMT7
SMPD3
Chr. 18

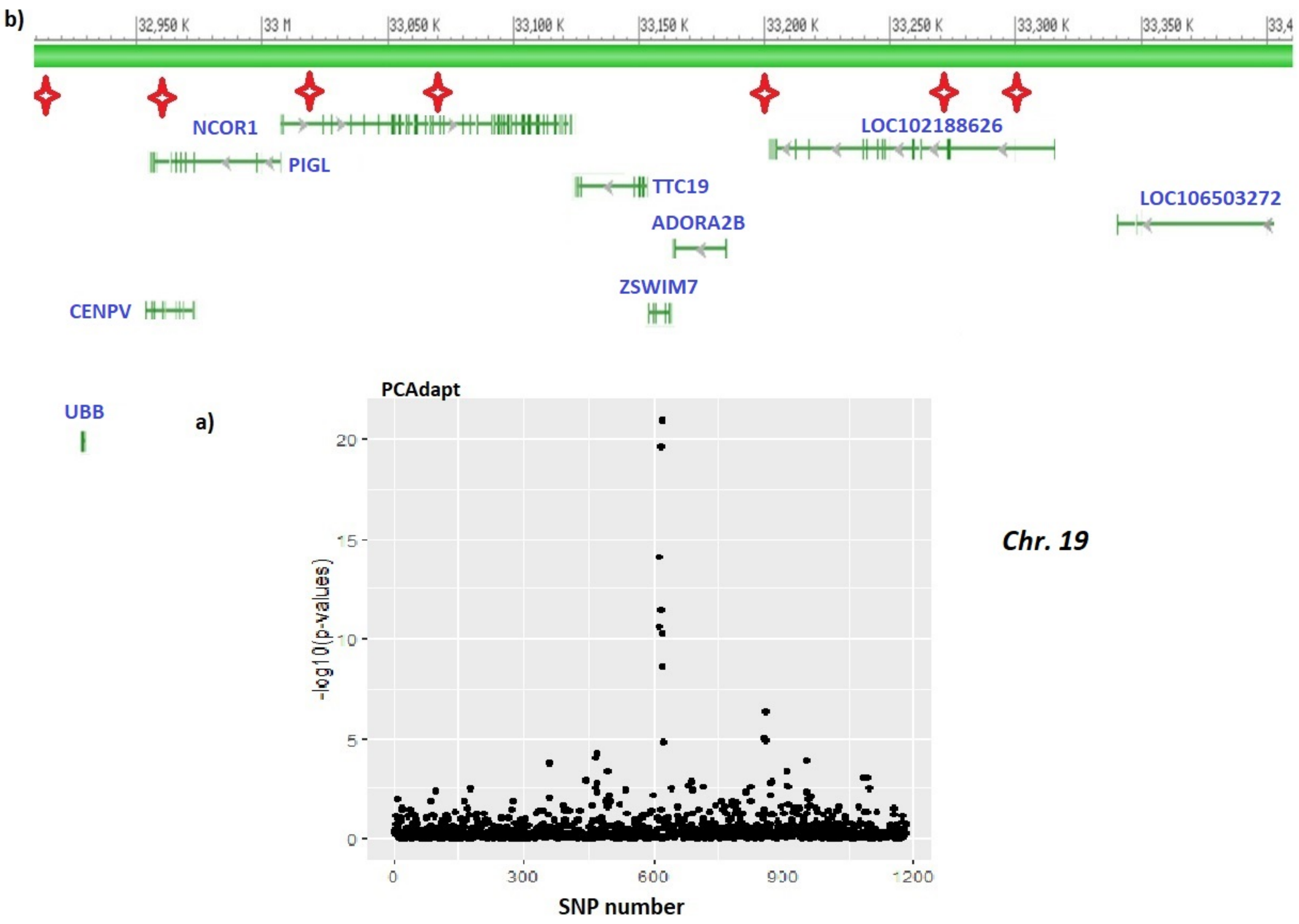

b)
32,950 K
33 M
33,050 K
33,100 K
33,150 K
33,200 K
33,250 K
33,300 K
33,350 K
NCOR1
PIGL
TTC19
ADORA2B
ZSWIM7
CENPV
UBB
LOC102188626
LOC106503272
a)
PCAdapt
-log10(p-values)
SNP number
0
300
600
900
1200
5
10
15
20
Chr. 19

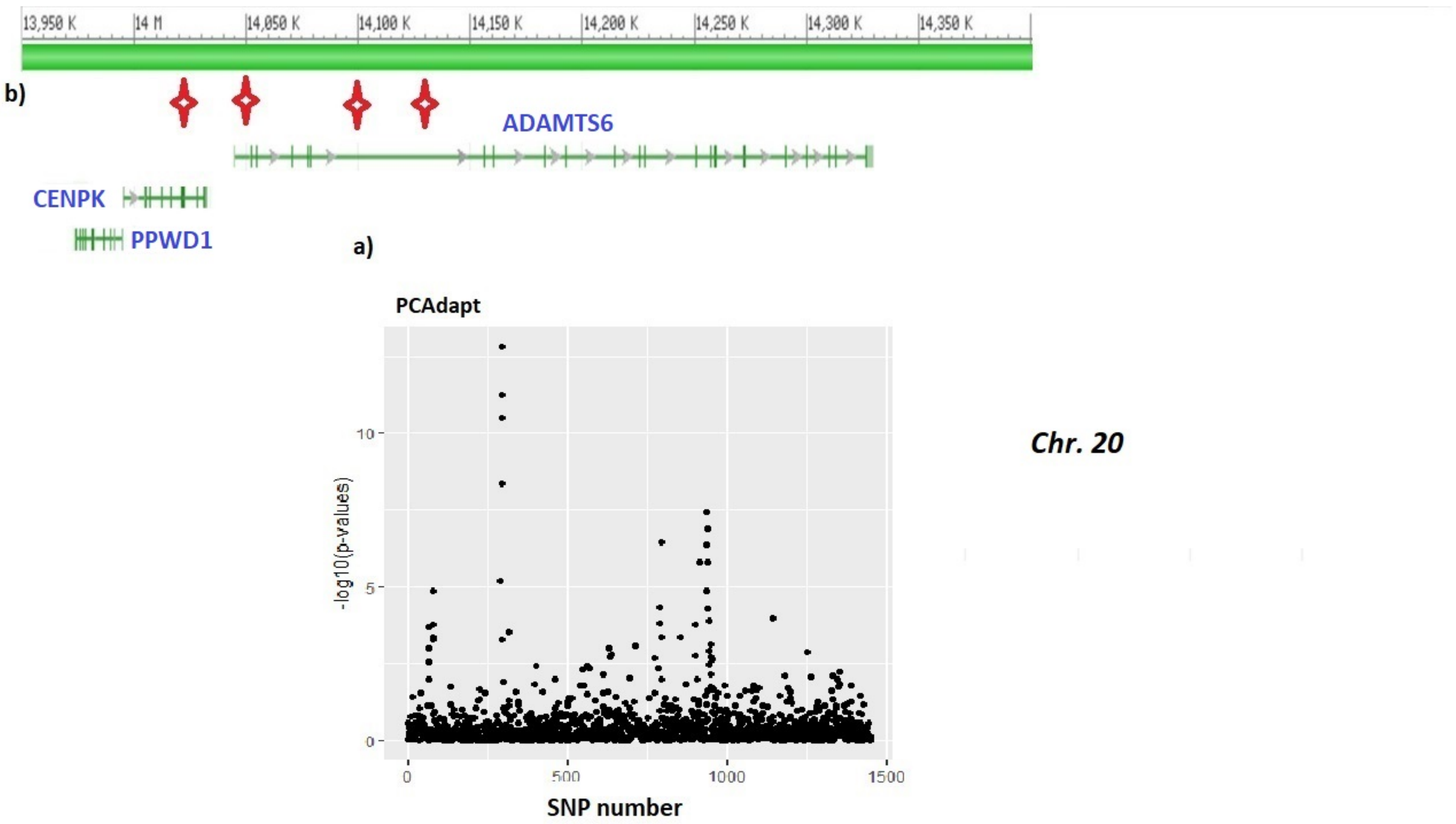
13,950 K
14 M
14,050 K
14,100 K
14,150 K
14,200 K
14,250 K
14,300 K
14,350 K
b)
ADAMTS6
CENPK
PPWD1
a)
PCAdapt
-log10(p-values)
SNP number
Chr. 20

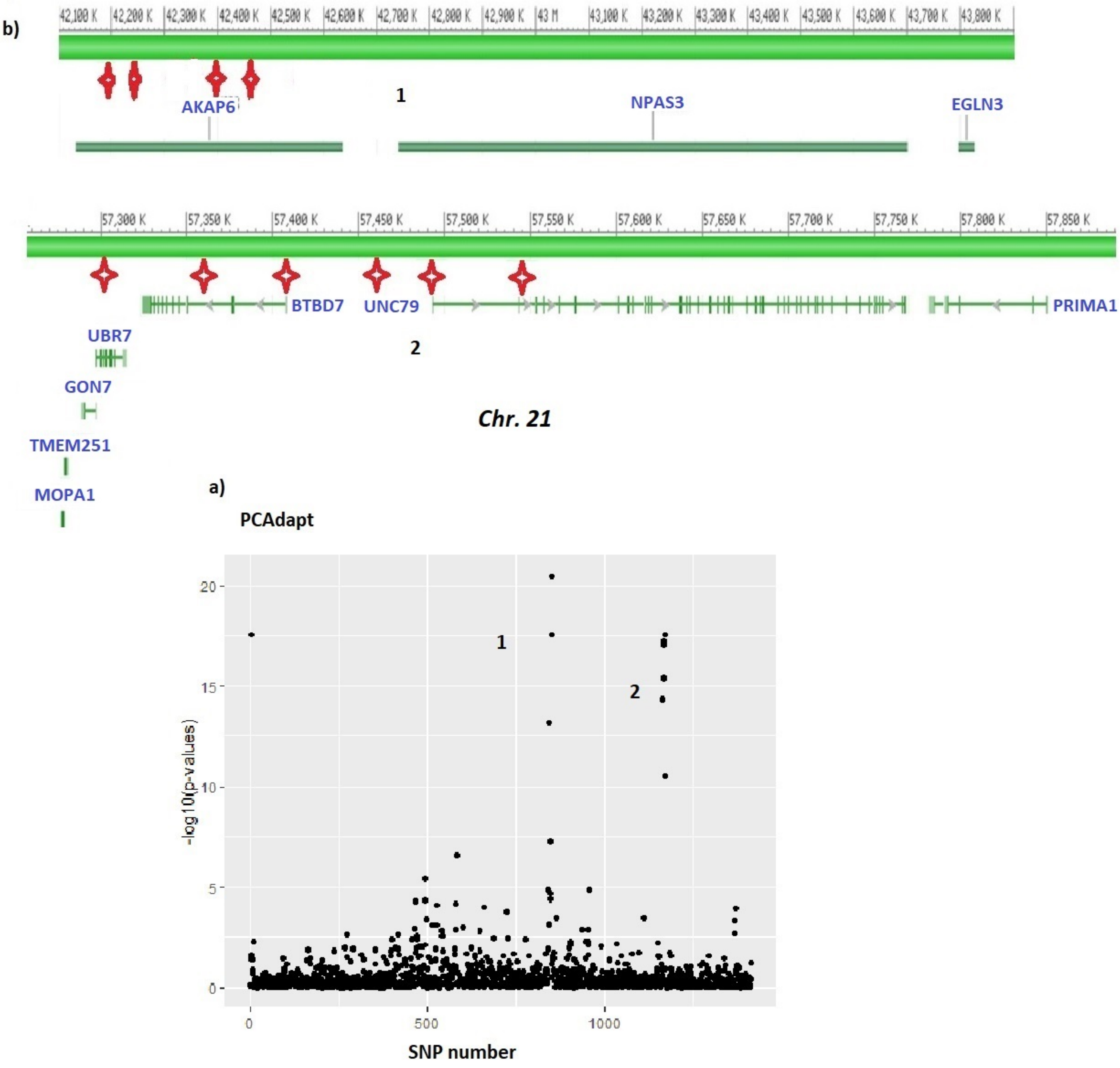
b)
42,100 K
42,200 K
42,300 K
42,400 K
42,500 K
42,600 K
42,700 K
42,800 K
42,900 K
43 M
43,100 K
43,200 K
43,300 K
43,400 K
43,500 K
43,600 K
43,700 K
43,800 K
1
AKAP6
NPAS3
EGLN3
57,300 K
57,350 K
57,400 K
57,450 K
57,500 K
57,550 K
57,600 K
57,650 K
57,700 K
57,750 K
57,800 K
57,850 K
BTBD7
UNC79
PRIMA1
UBR7
2
GON7
TMEM251
MOPA1
Chr. 21
a)
PCAdapt
-log10(p-values)
SNP number
1
2
0
5
10
15
20
0
500
1000

a)

b)

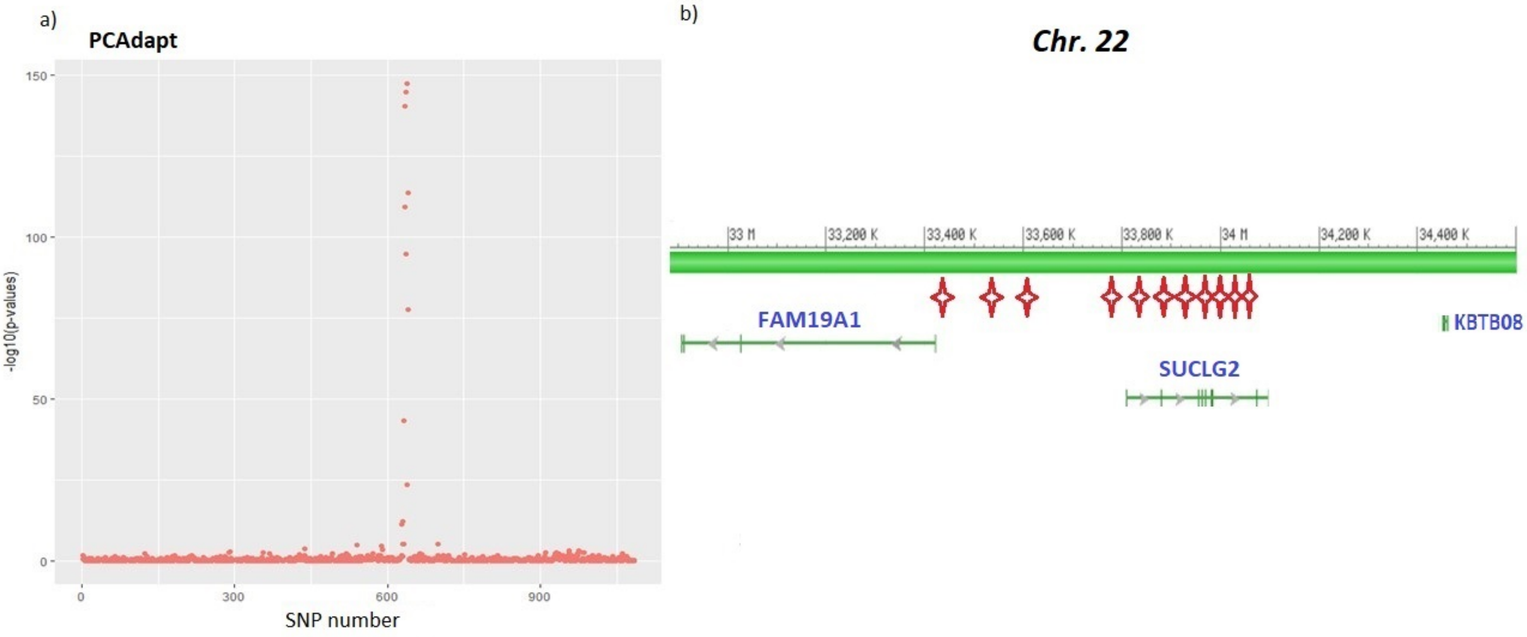
PCAdapt
-log10(p-values)
150
100
50
0
0
300
600
900
SNP number
Chr. 22
33 M
33,200 K
33,400 K
33,600 K
33,800 K
34 M
34,200 K
34,400 K
FAM19A1
KBTB08
SUCLG2

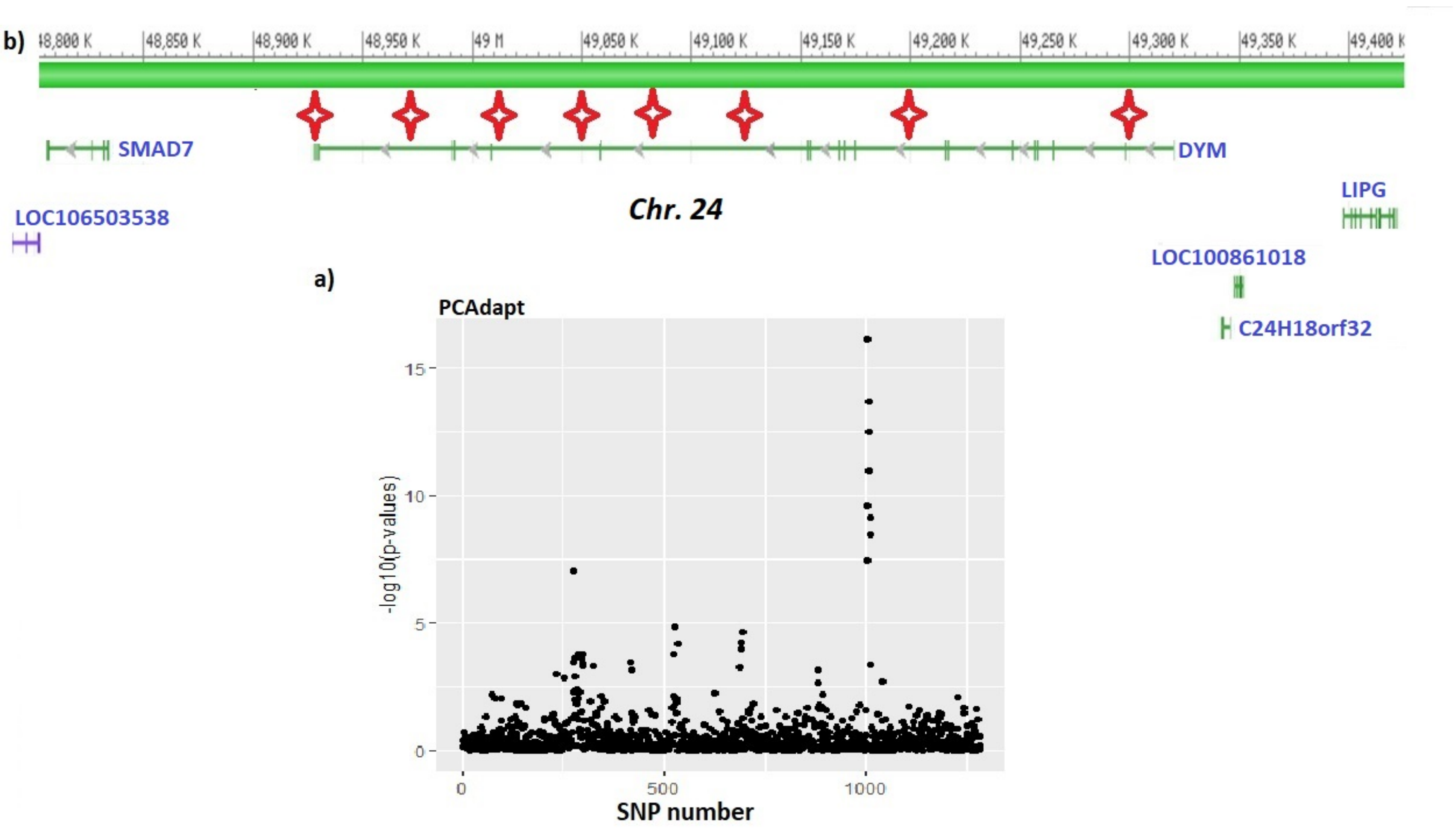

b)
48,800 K
48,850 K
48,900 K
48,950 K
49 M
49,050 K
49,100 K
49,150 K
49,200 K
49,250 K
49,300 K
49,350 K
49,400 K
SMAD7
DYM
LOC106503538
Chr. 24
LIPG
LOC100861018
C24H18orf32
a)
PCAdapt
-log10(p-values)
SNP number

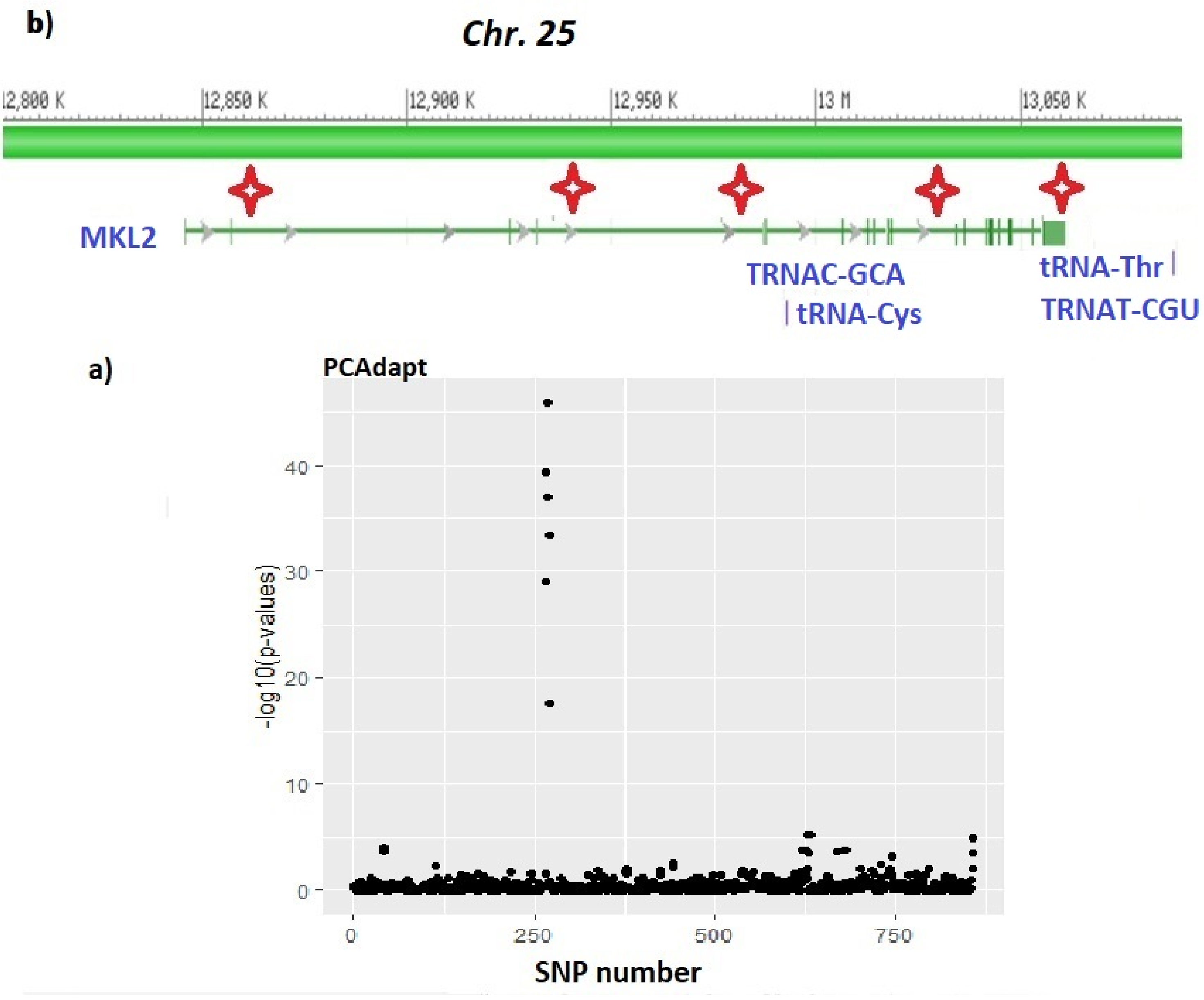

b)
Chr. 25
12,800 K
12,850 K
12,900 K
12,950 K
13 M
13,050 K
MKL2
TRNAC-GCA
tRNA-Cys
tRNA-Thr
TRNAT-CGU
a)
PCAdapt
-log10(p-values)
0
10
20
30
40
0
250
500
750
SNP number

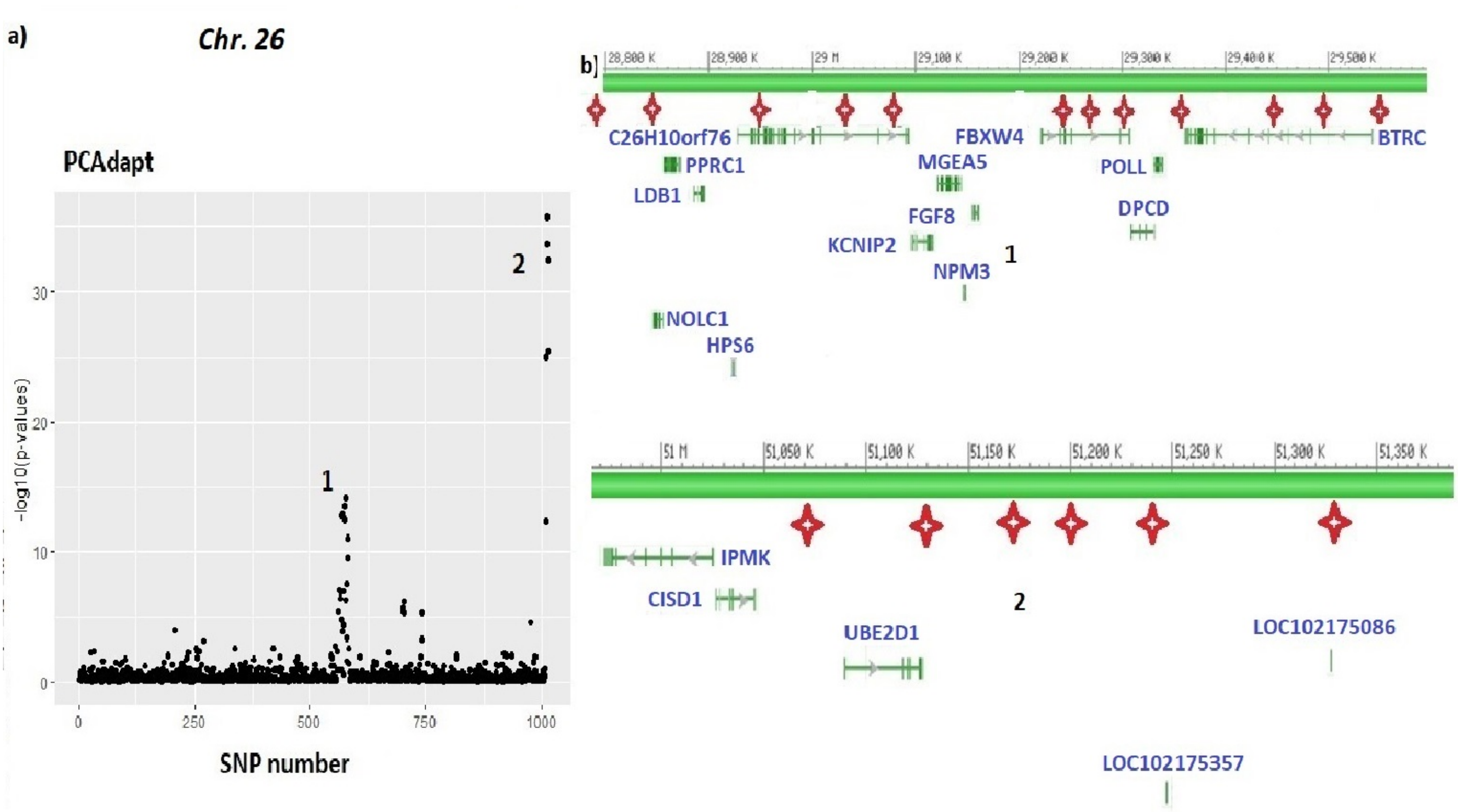
a)
Chr. 26
PCAdapt
-log10(p-values)
SNP number
1
2
b)
C26H10orf76
PPRC1
LDB1
MGEA5
FGF8
KCNIP2
NPM3
FBXW4
POLL
DPCD
BTRC
NOLC1
HPS6
IPMK
CISD1
UBE2D1
LOC102175086
LOC102175357

Supplementary Table 1

Name	Abbrev.	Origin	Sample size considered	Project_Acronym	Contributor_Institution
Barki	BRK	EGYPT	20	ISU-Egypt goat project	IASTATE-ICARDA
Nubian	NBN	EGYPT	20	INTA_project	INTA
Oasis	OSS	EGYPT	20	ISU-Egypt goat project	IASTATE-ICARDA
Saidi	SID	EGYPT	20	ISU-Egypt goat project	IASTATE-ICARDA
Tunisian	TUN	TUNISIA	20	Spanish_project1	Laboratorio de Genetica Molecular Aplicad/Universidad de Cordoba
Black	bla	MOROCCO	22	NEXTGEN	Nextgen consortium
Draa	dra	MOROCCO	14	NEXTGEN	Nextgen consortium
Nord	nor	MOROCCO	8	NEXTGEN	Nextgen consortium
Arabia	AR	ALGERIA	12	-	Unité de Génétique Moléculaire Animale (Limoges, France)
Kabyle	NK	ALGERIA	12	-	Unité de Génétique Moléculaire Animale (Limoges, France)
Makatia	MK	ALGERIA	12	-	Unité de Génétique Moléculaire Animale (Limoges, France)
M'Zabite	MZ	ALGERIA	12	-	Unité de Génétique Moléculaire Animale (Limoges, France)
Guera	GUE	MALI	20	AGIN	USDA
Maure	MAU	MALI	14	AGIN	USDA
Naine	NAI	MALI	17	AGIN	USDA
Peulh	PEU	MALI	20	AGIN	USDA
Soudanaise	SDN	MALI	20	AGIN	USDA
Targui	TAR	MALI	20	AGIN	USDA
Djallonke	DJA	BURKINA FASO	12	MICINN-FEDER AGL2011-27585	SERIDA-Deva (Spain) & INERA-CNRST (Burkina Faso)
Sahel	SAH	BURKINA FASO	15	MICINN-FEDER AGL2011-27585	SERIDA-Deva (Spain) & INERA-CNRST (Burkina Faso)
Cameroon Goat	CAM	CAMEROON	20	AGIN	USDA
West African Dwarf	WADc	CAMEROON	20	AGIN	USDA
West African Dwarf	WAD	CAMEROON	20	AGIN	USDA
Red Sokoto	RSK	NIGERIA	20	AGIN	USDA
Sahel	SHL	NIGERIA	20	AGIN	USDA
Abergelle	ABR	ETHIOPIA	20	AGIN	USDA
Gumez	GUM	ETHIOPIA	20	AGIN	USDA
Keffa	KEF	ETHIOPIA	20	AGIN	USDA
Woyito Guji	WYG	ETHIOPIA	20	AGIN	USDA
Maasai	MAA	TANZANIA	20	AGIN	USDA
Pare White	PRW	TANZANIA	20	AGIN	USDA
Sonjo	SNJ	TANZANIA	20	AGIN	USDA
Gogo	GOG	TANZANIA	13	AGIN	USDA
Galla	GAL	KENYA	20	AGIN	USDA
Small East African	SEAk	KENYA	20	AGIN	USDA

Burundi goat	BUR	BURUNDI	20	FAO-PTP	FAO-PTP
Karamonja	KAR	UGANDA	20	AGIN	USDA
Mubende	MUB	UGANDA	20	AGIN	USDA
Nganda	NGD	UGANDA	11	AGIN	USDA
Small East African	SEA	UGANDA	15	AGIN	USDA
Sebei	SEB	UGANDA	20	AGIN	USDA
Landin	LND	MOZAMBIQUE	20	AGIN	USDA
Mashona	MSH	ZIMBABWE	20	AGIN	USDA
Matebele	MTB	ZIMBABWE	20	AGIN	USDA

Supplementary Table 2

**Bioclim variable**	**Definition**
BIO_1	Annual Mean Temperature[2]
BIO_5	Max Temperature[2] of Warmest Month
BIO_6	Min Temperature[2] of Coldest Month
BIO_{12}	Annual Precipitation[1]
BIO_{13}	Precipitation[1] of Wettest Month
BIO_{14}	Precipitation[1] of Driest Month
BIO_{15}	Precipitation[1] Seasonality (Coefficient of Variation)

[1] Precipitation is expressed in millimeters.

[2] Temperature is expressed in °C×10.

Supplementary Table 3

	selection signature area identified by PCAdapt	SNP identified by PCAdapt in the selection signature area: name (position)<br><br>Gene(s) identified	selection signature area identified by LFMM	SNP identified by LFMM in the selection signature area: name (position)<br><br>Gene(s) identified	selection signature area identified by Bayscan	SNP identified by Bayescan in the selection signature area: name (position)<br><br>Gene(s) identified	selection signature area identified by Hapflk	SNP identified by Hapflk: name (position)<br><br>Gene(s) identified; in red SNP identified by Caviar
**West/ Central African goats**								
Chr. 2			15786051-16322293	snp3621-scaffold1113-144193 (15786051)<br>snp3623-scaffold1113-211185 (15853495)<br>snp3629-scaffold1113-489404* (16132803)<br>snp3631-scaffold1113-569475* (16211206)<br>snp3634-scaffold1113-679544* (16322293)<br><br>DIS3L2 DIS3 like 3'-5' exoribonuclease 2<br>Gene ID: 102179613<br>NPPC natriuretic peptide C<br>Gene ID: 100861580<br>PDE6D phosphodiesterase 6D<br>Gene ID: 102179145<br>COPS7B COP9 signalosome subunit 7B Gene ID: 102188989<br>PTMA prothymosin alpha<br>Gene ID: 102175998<br>LOC108637527 uncharacterized LOC108637527 Gene ID: 108637527				
Chr. 5			22987063-23578233	snp38434-scaffold486-2770180 (22987063)*<br>snp38428-scaffold486-2496390 (23261080)*<br>snp38422-scaffold486-2234526 (23524159)*				

				snp38421-scaffold486-2181656 (23578233)*<br><br>SOCS2 suppressor of cytokine signaling 2 Gene ID: 102178979<br>CRADD CASP2 and RIPK1 domain containing adaptor with death domain<br>Gene ID: 102179736				
Chr. 6	59436318-59715618	snp27122-scaffold289-2971402 (59436318)*<br>snp27124-scaffold289-3064928 (59530632)*<br>snp27125-scaffold289-3119585 (59585594)<br>snp27126-scaffold289-3163226 (59629950)*<br>snp27127-scaffold289-3248302 (59715618)*<br><br>UBE2K ubiquitin conjugating enzyme E2 K Gene ID: 100860797	45415286-45625340	snp12879-scaffold1498-8679 (45415286)<br>snp12880-scaffold1498-47642 (45452714)*<br>snp12881-scaffold1498-89673 (45494742)<br>snp12882-scaffold1498-142431 (45547830)*<br>snp12883-scaffold1498-190653 (45596401)*<br>snp12884-scaffold1498-220976 (45625340)*<br><br>SLC34A2 solute carrier family 34 member 2<br>Gene ID: 100861296				

		PDS5A PDS5 cohesin associated factor A Gene ID: 102173171<br>LOC102173648 NEDD4-binding protein 2 Gene ID: 102173648						
Chr. 7			87674531-87956716	snp29299-scaffold316-678611 (87674531)<br>snp29298-scaffold316-647237 (87705987)*<br>snp29297-scaffold316-606485 (87746830)*<br>snp29294-scaffold316-447400 (87907470)*<br>snp29293-scaffold316-398273 (87956716)*<br><br>RAPGEF6 Rap guanine nucleotide exchange factor 6 [Capra hircus (goat)]<br>Gene ID: 102191417<br>FNIP1 folliculin interacting protein 1 Gene ID: 102182584<br>MEIKIN meiotic kinetochore factor Gene ID: 102182299				

Chr. 10	64438812-65229754	snp33752-scaffold397-1116383 (64438812)<br>snp33754-scaffold397-1191279 (64514259)<br>snp33757-scaffold397-1318426 (64640998)<br>snp33758-scaffold397-1371358 (64695254)*<br>snp33760-scaffold397-1485897 (64811188)*<br>snp33764-scaffold397-1657210 (64982985)*<br>snp33766-scaffold397-1739113 (65072121)<br>snp33768-scaffold397-1829574 (65163149)<br>snp33769-scaffold397-1895992 (65229754)<br><br>mRNA-UBR1 ubiquitin protein ligase E3 component n-recognin 1/Gene ID: 102169592<br>EPB42 erythrocyte membrane protein band 4.2<br>Gene ID: 102170150<br>TMEM62 transmembrane protein 62 Gene ID: 102176612<br>TTBK2 tau tubulin kinase 2/Gene ID: 102169294<br>CDAN1 codanin 1<br>Gene ID: 102176331			64438812-65229754	snp33752-scaffold397-1116383 (64438812)<br>snp33757-scaffold397-1318426 (64640998)<br>snp33758-scaffold397-1371358 (64695254)<br>snp33760-scaffold397-1485897 (64811188)<br>snp33764-scaffold397-1657210 (64982985)<br>snp33768-scaffold397-1829574 (65163149)<br>snp33769-scaffold397-1895992 (65229754)<br><br>mRNA-UBR1 ubiquitin protein ligase E3 component n-recognin 1/Gene ID: 102169592	63610639-64982985	snp33733-scaffold397-278423 (63610639)<br>snp33735-scaffold397-347360 (63680312)<br>snp33736-scaffold397-380352 (63714566)<br>snp33737-scaffold397-415738 (63750827)<br>snp33738-scaffold397-480829 (63818132)<br>snp33739-scaffold397-574930 (63912104)<br>snp33740-scaffold397-616889 (63954235)<br>snp33741-scaffold397-666823 (64000839)<br>snp33753-scaffold397-1161763 (64484827)<br>snp33754-scaffold397-1191279 (64514259)<br>snp33755-scaffold397-1224407 (64547391)<br>snp33757-scaffold397-1318426 (64640998)<br>snp33758-scaffold397-1371358 (64695254)<br>snp33760-scaffold397-1485897 (64811188)<br>snp33764-scaffold397-1657210 (64982985)<br><br>mRNA-UBR1 ubiquitin protein ligase E3 component n-recognin 1/Gene ID: 102169592
Chr. 12	44050377-44471339	snp53665-scaffold817-739382 (44050377)*						

		snp53664-scaffold817-708766 (44081824)*<br>snp53663-scaffold817-677742 (44113302)*<br>snp53662-scaffold817-625200 (44165707)*<br>snp53661-scaffold817-570565 (44221034)*<br>snp53659-scaffold817-464982 (44327480)*<br>snp53656-scaffold817-324137 (44471339)*<br><br>PCDH9 protocadherin 9 Gene ID: 102175035<br>KLHL1 kelch like family member 1 Gene ID: 102176619						
	47433756-47769497	snp11178-scaffold140-2375316 (47433756)*<br>snp11177-scaffold140-2325487 (47483765)*<br>snp11176-scaffold140-2283744 (47525157)*<br>snp11175-scaffold140-2243536 (47566623)*<br>snp11174-scaffold140-2167549 (47644055)*<br>snp11173-scaffold140-2130863 (47681353)*<br>snp11172-scaffold140-2092549 (47719708)*<br>snp11171-scaffold140-2043461 (47769497)*<br><br>PCDH9 protocadherin 9 Gene ID: 102175035<br>KLHL1 kelch like family member 1 Gene ID: 102176619						

	50152165-50676638	snp30394-scaffold335-315825 (50152165)*<br>snp30395-scaffold335-354367 (50190714)*<br>snp30396-scaffold335-385244 (50221592)*<br>snp30397-scaffold335-418126 (50253401)*<br>snp30399-scaffold335-501928 (50327948)*<br>snp30400-scaffold335-530813 (50356882)*<br>snp30402-scaffold335-625380 (50451600)*<br>snp30404-scaffold335-730262 (50553986)*<br>snp30405-scaffold335-772607 (50596847)*<br>snp30406-scaffold335-807385 (50631622)<br>snp30407-scaffold335-852300 (50676638)<br><br>RNF17 ring finger protein 17/ Gene ID: 102172365<br>CENPJ centromere protein J/ Gene ID: 102173755<br>PARP4 poly(ADP-ribose) polymerase family member 4/ Gene ID: 102173202<br>PSPC1 paraspeckle component 1/ Gene ID: 102171798<br>MPHOSPH8 M-phase phosphoprotein 8/ Gene ID: 102172075<br>ZMYM5 zinc finger MYM-type containing 5/ Gene ID: 102172456<br>ZMYM2 zinc finger MYM-type containing 2/ Gene ID: 102171989<br>GJB2 gap junction protein beta 2 Gene ID: 102171720			50553986-50755554	snp30404-scaffold335-730262 (50553986)<br>snp30405-scaffold335-772607 (50596847)<br>snp30409-scaffold335-931312 (50755554)<br><br>ZMYM5 zinc finger MYM-type containing 5/ Gene ID: 102172456<br>ZMYM2 zinc finger MYM-type containing 2/ Gene ID: 102171989			

	60710546-60950668	snp50175-scaffold717-4447356 (60710546)*<br>snp50172-scaffold717-4328533 (60829727)*<br>snp50171-scaffold717-4286071 (60872237)<br>snp50170-scaffold717-4247907 (60910543)*<br>snp50169-scaffold717-4207960 (60950668)*<br><br>NBEA neurobeachin/Gene ID: 102186605	60220203-60950668	snp50186-scaffold717-4934533 (60220203)*<br>snp50175-scaffold717-4447356 (60710546)*<br>snp50172-scaffold717-4328533 (60829727)*<br>snp50171-scaffold717-4286071 (60872237)<br>snp50170-scaffold717-4247907 (60910543)*<br>snp50169-scaffold717-4207960 (60950668)*<br><br>NBEA neurobeachin/Gene ID: 102186605	60710546-60950668	snp50175-scaffold717-4447356 (60710546)<br>snp50172-scaffold717-4328533 (60829727)<br>snp50170-scaffold717-4247907 (60910543)<br>snp50169-scaffold717-4207960 (60950668)<br><br>NBEA neurobeachin/Gene ID: 102186605	60637258-60950668	snp50177-scaffold717-4519853 (60637258)<br>snp50175-scaffold717-4447356 (60710546)<br>snp50174-scaffold717-4415291 (60742599)<br>snp50172-scaffold717-4328533 (60829727)<br>snp50171-scaffold717-4286071 (60872237)<br>snp50170-scaffold717-4247907 (60910543)<br>snp50169-scaffold717-4207960 (60950668)<br><br>NBEA neurobeachin/Gene ID: 102186605
Chr. 19	32969117-33307741	snp28431-scaffold303-2935885 (32969117)*<br>snp28432-scaffold303-2995232 (33028814)*<br>snp28433-scaffold303-3044995 (33078622)*<br>snp28436-scaffold303-3170123 (33202721)*<br>snp28437-scaffold303-3243981 (33274858)<br>snp28438-scaffold303-3276620 (33307741)<br><br>NCOR1 nuclear receptor corepressor 1 Gene ID: 102188080<br>PIGL phosphatidylinositol glycan anchor biosynthesis class L Gene ID: 102178568<br>ADORA2B adenosine A2b receptor Gene ID: 102179134<br>LOC102188626 cytospin-B Gene ID: 102188626						

Chr.21	42315959-42638483	snp38786-scaffold492-948315 (42315959)* snp38785-scaffold492-916281 (42348021)* snp38784-scaffold492-867472 (42397252)* snp38782-scaffold492-798404 (42466574)* snp38779-scaffold492-700111 (42564910)* snp38778-scaffold492-666628 (42598485)* snp38777-scaffold492-626818 (42638483)*<br><br>AKAP6 A-kinase anchoring protein 6 Gene ID: 102179241						
Chr. 25	12862316-13062061	snp8762-scaffold1314-669806 (12862316) snp8761-scaffold1314-639651 (12891663)* snp8760-scaffold1314-588233 (12943285)* snp8759-scaffold1314-543139 (12988932)* snp8758-scaffold1314-501381 (13031029)* snp8757-scaffold1314-470287 (13062061)<br><br>MKL2/MRTFB myocardin related transcription factor B Gene ID: 102176092						
Chr. 26	28957011-29548698	snp41104-scaffold532-649324 (28957011) snp41105-scaffold532-692721 (29001092) snp41107-scaffold532-776372 (29084628) snp41109-scaffold532-897192 (29205431)			None		None	

		snp41110-scaffold532-933054 (29241421)*<br>snp41111-scaffold532-965874 (29274605)*<br>snp41112-scaffold532-1001039 (29309963)*<br>snp41113-scaffold532-1051457 (29360578)*<br>snp41114-scaffold532-1082302 (29391742)*<br>snp41116-scaffold532-1145437 (29455378)*<br>snp41117-scaffold532-1188003 (29498263)*<br>snp41118-scaffold532-1238182 (29548698)<br><br>FBXW4 F-box and WD repeat domain containing 4/Gene ID: 102178974<br>BTRC beta-transducin repeat containing E3 ubiquitin protein ligase/Gene ID: 102184871<br>ARMH3 armadillo like helical domain containing 3 Gene ID: 102187828						
**East African goats**								
Chr. 6	85978469-86198470	snp59416-scaffold980-293987 (85978469)*<br>snp59417-scaffold980-295173 (85979655)*<br>snp59426-scaffold980-307288 (85991683)*<br>snp60000-CSN1S1-ex17 (85993405)*<br>snp59427-scaffold980-309132 (85994156)*<br>snp59428-scaffold980-310410 (85995436)*<br>snp59429-scaffold980-310523 (85995549)*						

		snp59430-scaffold980-311053 (85996079)*<br>snp59431-scaffold980-311510 (85996534)*<br>snp59432-scaffold980-311547 (85996571)*<br>snp59434-scaffold980-322977 (86007956)*<br>snp59438-scaffold980-323773 (86008752)*<br>snp59439-scaffold980-324311 (86009290)*<br>snp59440-scaffold980-324828 (86009807)*<br>6snp59441-scaffold980-325353 (86010332)*<br>snp59442-scaffold980-364892 (86050088)*<br>snp59450-scaffold980-400667 (86085897)*<br>snp59451-scaffold980-402598 (86087828)*<br>snp59454-scaffold980-403305 (86088536)*<br>snp59455-scaffold980-407817 (86093124)<br>snp59458-scaffold980-434555 (86118732)<br>snp59459-scaffold980-469488 (86155374)<br>snp59460-scaffold980-512583 (86198470)<br><br>**CSN1S1 casein alpha s1/Gene ID: 100750242**<br>**CSN2 casein beta/Gene ID: 100860784**<br>**CSN1S2 as2-casein/Gene ID: 100861229**						
Chr. 11	94156441-94498221	snp33904-scaffold4-769205 (94156441)*<br>snp33905-scaffold4-839979 (94227241)*						

		snp33906-scaffold4-879629 (94267046)*<br>snp33907-scaffold4-931911 (94319962)*<br>snp33908-scaffold4-982201 (94370706)*<br>snp33909-scaffold4-1013632 (94402272)*<br>snp33911-scaffold4-11094040 (94498221)*<br><br>DENND1A DENN domain containing 1A ID: 102185372						
Chr. 12	43632105-45137234	snp53672-scaffold817-1154415 (43632105)<br>snp53669-scaffold817-920869 (43869668)*<br>snp53668-scaffold817-874431 (43916255)*<br>snp53666-scaffold817-776660 (44012257)*<br>snp53665-scaffold817-739382 (44050377)*<br>snp53663-scaffold817-677742 (44113302)*<br>snp53661-scaffold817-570565 (44221034)*<br>snp53660-scaffold817-516359 (44275453)*<br>snp53657-scaffold817-364334 (44429883)*<br>snp53650-scaffold817-85068 (44712814)*<br>snp38237-scaffold481-24997 (44951580)*<br>snp38238-scaffold481-65343 (44992162)*<br>snp38241-scaffold481-212607 (45137234)*						

		PCDH9 protocadherin 9 Gene ID: 102175035<br>KLHL1 kelch like family member 1 Gene ID: 102176619						
	50047466-50515766	snp30392-scaffold335-200469 (50047466)*<br>snp30393-scaffold335-255434 (50102642)*<br>snp30394-scaffold335-315825 (50152165)*<br>snp30396-scaffold335-385244 (50221592)*<br>snp30397-scaffold335-418126 (50253401)*<br>snp30400-scaffold335-530813 (50356882)*<br>snp30402-scaffold335-625380 (50451600)*<br>snp30403-scaffold335-692736 (50515766)*<br><br>ATP12A ATPase H+/K+ transporting non-gastric alpha2 subunit ID: 102172639<br>RNF17 ring finger protein Gene ID: 102172365<br>CENPJ centromere protein J Gene ID: 102173755<br>MPHOSPH8 M-phase phosphoprotein 8 Gene ID: 102172075<br>PARP4 poly(ADP-ribose) polymerase family member 4 Gene ID: 102173202<br>PSPC1 paraspeckle component 1 Gene ID: 102171798<br>ZMYM5 zinc finger MYM-type containing 5 Gene ID: 102172456						
Chr. 13	46458560-46473488	snp56302-scaffold881-1647723 (46458560)*						

		snp56304-scaffold881-1648328 (46459167)*<br>snp56308-scaffold881-1657525 (46468393)*<br>snp56310-scaffold881-1658893 (46469761)*<br>snp56312-scaffold881-1659181 (46470049)*<br>snp56317-scaffold881-1659485 (46470353)*<br>snp56318-scaffold881-1660022 (46470890)*<br>snp56319-scaffold881-1662266 (46473134)*<br>snp56320-scaffold881-1662295 (46473163)*<br>snp56322-scaffold881-1662620 (46473488)*<br><br>PRNP prion protein/Gene ID: 102169975						
Chr. 16	43284994-43405682	snp44063-scaffold597-301714 (43284994)*<br>snp44064-scaffold597-331119 (43314528)*<br>snp44065-scaffold597-389116 (43371509)*<br>snp44066-scaffold597-423262 (43405682)*<br><br>RERE arginine-glutamic acid dipeptide repeats<br>Gene ID: 102178646						
Chr. 17	36231019-36715246	snp43563-scaffold581-1414591 (36231019)<br>snp43562-scaffold581-1378450 (36267202)*<br>snp43561-scaffold581-1298381 (36347413)*<br>snp43560-scaffold581-1262535 (36383399)*<br>snp43559-scaffold581-1216278 (36429504)*						

		snp43558-scaffold581-1163312 (36482606)*<br>snp43554-scaffold581-978133 (36668503)*<br>snp43553-scaffold581-931227 (36715246)<br><br>SPATA5 spermatogenesis associated 5 Gene ID: 102180415						
Chr. 18	36786664-37006519	snp7212-scaffold1266-2397446 (36786664)*<br>snp7211-scaffold1266-2368797 (36815394)*<br>snp7210-scaffold1266-2322163 (36862059)*<br>snp7209-scaffold1266-2252567 (36931726)*<br>snp7208-scaffold1266-2217995 (36966108)*<br>snp7207-scaffold1266-2177748 (37006519)*<br><br>NFATC3 nuclear factor of activated T cells 3/Gene ID: 102180958<br>DUS2 dihydrouridine synthase 2/Gene ID: 102190541<br>SLC7A6 solute carrier family 7 member 6/Gene ID: 102168568<br>SLC7A6OS solute carrier family 7 member 6 opposite strand/Gene ID: 102168851<br>ESRP2 epithelial splicing regulatory protein 2/Gene ID: 102181230<br>PLA2G15 phospholipase A2 group XV/Gene ID: 102168288<br>SMPD3 sphingomyelin phosphodiesterase 3/Gene ID: 102169323<br>PRMT7 protein arginine methyltransferase 7/Gene ID: 102169616						

Chr 19.	32913594-33307741	snp28430-scaffold303-2880476 (32913594)*<br>snp28431-scaffold303-2935885 (32969117)*<br>snp28432-scaffold303-2995232 (33028814)*<br>snp28433-scaffold303-3044995 (33078622)*<br>snp28436-scaffold303-3170123 (33202721)*<br>snp28437-scaffold303-3243981 (33274858)*<br>snp28438-scaffold303-3276620 (33307741)*<br><br>NCOR1 nuclear receptor corepressor 1 Gene ID: 102188080<br>PIGL phosphatidylinositol glycan anchor biosynthesis class L Gene ID: 102178568<br>ADORA2B adenosine A2b receptor Gene ID: 102179134<br>LOC102188626 cytospin-B Gene ID: 102188626						
Chr. 20	14021075-14132568	snp43214-scaffold575-1067749 (14021075)*<br>snp43215-scaffold575-1103515 (14056838)*<br>snp43216-scaffold575-1149259 (14103234)*<br>snp43217-scaffold575-1178652 (14132568)*<br><br>ADAMTS6 ADAM metallopeptidase with thrombospondin type 1 motif 6 Gene ID: 102172391						
Chr. 21	42193046-42466574	snp38788-scaffold492-1070403 (42193046)*<br>snp38787-scaffold492-1005477 (42257962)						

		snp38784-scaffold492-867472 (42397252)*<br>snp38782-scaffold492-798404 (42466574)*						
	57303973-57544623	snp12081-scaffold1446-830756 (57303973)*<br>snp12080-scaffold1446-769570 (57366338)*<br>snp12079-scaffold1446-720896 (57415158)*<br>snp12078-scaffold1446-676380 (57459731)*<br>snp12077-scaffold1446-640296 (57496640)*<br>snp12076-scaffold1446-592612 (57544623)*<br><br>UBR7 ubiquitin protein ligase E3 component n-recognin 7 Gene ID: 106503378<br>BTBD7 BTB domain containing 7 Gene ID: 102169525<br>UNC79 unc-79 homolog, NALCN channel complex subunit Gene ID: 102169806						
Chr. 22	33429708-34062919	snp13807-scaffold154-687631 (33429708)*<br>snp13805-scaffold154-601155 (33517389)*<br>snp13803-scaffold154-526016 (33594470)*<br>snp13799-scaffold154-347555 (33769647)*<br>snp13798-scaffold154-300085 (33818637)*						

		snp13797-scaffold154-243475 (33876201)*<br>snp13796-scaffold154-213942 (33905179)*<br>snp13795-scaffold154-182886 (33936550)*<br>snp13794-scaffold154-139936 (33979489)*<br>snp13793-scaffold154-98284 (34020913)*<br>snp13792-scaffold154-56173 (34062919)*<br><br>TAFA1 TAFA chemokine like family member 1/Gene ID: 102177386<br><br>SUCLG2 succinate-CoA ligase GDP-forming beta subunit/Gene ID: 102190648						
Chr. 24	48936361-49308788	snp7678-scaffold1277-988620 (48936361)*<br>snp7677-scaffold1277-950356 (48974579)<br>snp7676-scaffold1277-912193 (49013533)*<br>snp7675-scaffold1277-875356 (49050458)*<br>snp7674-scaffold1277-840997 (49084985)*<br>snp7673-scaffold1277-804880 (49121131)*<br>snp7671-scaffold1277-724990 (49201088)*<br>snp7668-scaffold1277-617631 (49308788)*						

		DYM dymeclin Gene ID: 102190752						
Chr. 25	12862316-13062061	snp8762-scaffold1314-669806 (12862316)*<br>snp8761-scaffold1314-639651 (12891663)*<br>snp8760-scaffold1314-588233 (12943285)*<br>snp8759-scaffold1314-543139 (12988932)*<br>snp8758-scaffold1314-501381 (13031029)*<br>snp8757-scaffold1314-470287 (13062061)*<br><br>MKL2/MRTFB myocardin related transcription factor B Gene ID: 102176092						
Chr. 26	28717972-29548698	snp41099-scaffold532-409992 (28717972)<br>snp41100-scaffold532-448160 (28756401)<br>snp41102-scaffold532-545458 (28852960)*<br>snp41106-scaffold532-739950 (29048271)*<br>snp41107-scaffold532-776372 (29084628)<br>snp41110-scaffold532-933054 (29241421)*<br>snp41111-scaffold532-965874 (29274605)*<br>snp41112-scaffold532-1001039 (29309963)*<br>snp41113-scaffold532-1051457 (29360578)<br>snp41116-scaffold532-1145437 (29455378)<br>snp41117-scaffold532-1188003 (29498263)*<br>snp41118-scaffold532-1238182 (29548698)*						

		FBXW4 F-box and WD repeat domain containing 4/Gene ID: 102178974<br>BTRC beta-transducin repeat containing E3 ubiquitin protein ligase/Gene ID: 102184871<br>ARMH3 armadillo like helical domain containing 3 Gene ID: 102187828						
	51077585-51356112	snp10491-scaffold1374-333237 (51077585)*<br>snp10490-scaffold1374-278178 (51133089)*<br>snp10489-scaffold1374-236378 (51175722)*<br>snp10488-scaffold1374-205628 (51206533)*<br>snp10487-scaffold1374-162907 (51249587)*<br>snp10485-scaffold1374-59735 (51356112)*<br><br>UBE2D1 ubiquitin conjugating enzyme E2 D1<br>Gene ID: 100861089<br>LOC102175357 olfactory receptor 5W2-like<br>Gene ID: 102175357<br>LOC102175086 olfactory receptor 5D18-like<br>Gene ID: 102175086						

SNPs showing p-value<10^{-7} are indicated for each signature; *SNPs showing p-value<1.10^{-9}; in red SNPs identified by CAVIAR.

Supplementary Table 4

Chr	selection signature area identified by LFMM (climatic ranking on West-Central African goats)	SNP identified by LFMM in the selection signature area (*p-value<1e^{-9}): name (position)	Gene(s) identified in the selection signature
6	106818254-107080787	snp48577-scaffold690-1453770 106818254 *<br>snp48575-scaffold690-1363044 106909981 *<br>snp48574-scaffold690-1287975 106985701 *<br>snp48573-scaffold690-1253961 107019990 *<br>snp48571-scaffold690-1194013 107080787	**HS3ST1** heparan sulfate-glucosamine 3-sulfotransferase 1<br>Gene ID: 102169088
7	5883646-59140695	snp10047-scaffold1356-2830358 5883646*<br>snp10051-scaffold1356-2958531 5896538*<br>snp10055-scaffold1356-3132486 59140695	**LOC102175120** ankyrin repeat and KH domain-containing protein 1<br>Gene ID: 102175120<br>SLC4A9 solute carrier family 4 member 9<br>Gene ID: 102174654<br>**HBEGF** heparin binding EGF like growth factor<br>Gene ID: 102185462<br>PFDN1 prefoldin subunit 1 Gene ID: 102174203<br>CYSTM1 cysteine rich transmembrane module containing 1 Gene ID: 102173558
	59873465-60074382	snp30685-scaffold339-5400884 59873465*<br>snp30684-scaffold339-5350025 59924105*<br>snp30683-scaffold339-5315925 59958911*<br>snp30682-scaffold339-5258982 60015475<br>snp30680-scaffold339-5200334 60074382	**PAIP2** poly(A) binding protein interacting protein 2<br>Gene ID: 102169282<br>**MATR3** matrin 3 Gene ID: 102191234<br>SIL1[1] SIL1 nucleotide exchange factor Gene ID: 102190218
	83012031-83270010	snp36532-scaffold44-1147631 83012031<br>snp36531-scaffold44-1111699 83048104*<br>snp36530-scaffold44-1082592 8307730*<br>snp36528-scaffold44-1015836 83146013<br>snp36525-scaffold44-891864 83270010*	**GRAMD2B** GRAM domain containing 2B Gene ID: 102187856
	87674531-88320044	snp29299-scaffold316-678611 87674531*<br>snp29298-scaffold316-647237 87705987*<br>snp29293-scaffold316-398273 87956716<br>snp29291-scaffold316-323388 88033981*<br>snp29285-scaffold316-38052 88320044*	**RAPGEF6** Rap guanine nucleotide exchange factor 6 Gene ID: 102191417<br>FNIP1 folliculin interacting protein 1 Gene ID: 102182584<br>**MEIKIN** meiotic kinetochore factor Gene ID: 102182299
8	103672484-103808914	snp31879-scaffold356-3615024 103672484*<br>snp31880-scaffold356-3659036 103716215*<br>snp31881-scaffold356-3751390 103808914*	**TNFSF15** TNF superfamily member 15 Gene ID: 102178448<br>**TNFSF8** TNF superfamily member 8 Gene ID: 102178908
10	7117132-71294424	snp58393-scaffold947-4585767 7117132*<br>snp31008-scaffold343-21938 7122545*<br>snp31010-scaffold343-90869 71294424*	CDIN1 CDAN1 interacting nuclease 1 Gene ID: 102171792<br>DPH6[1] diphthamine biosynthesis 6 Gene ID: 102168266
12	59028158-59123625	snp50216-scaffold717-6126750 59028158*<br>snp50214-scaffold717-6063985 59091376*<br>snp50213-scaffold717-6032295 59123625*	**STARD13** StAR related lipid transfer domain containing 13 Gene ID: 102180134<br>(close to NBEA[1] neurobeachin Gene ID: 102186605)
18	39151654-39274043	snp7158-scaffold1266-41169 39151654*<br>snp9581-scaffold1344-19492 39212678*<br>snp9583-scaffold1344-80810 39274043*	ZFHX3 zinc finger homeobox 3 Gene ID: 102184837

23	1056349-10641101	snp51380-scaffold751-1991327 1056349*<br>snp51381-scaffold751-2033441 1060558*<br>snp51382-scaffold751-2068946 10641101*	**NUP153** nucleoporin 153 Gene ID: 102171943<br>**LOC106503510** uncharacterized LOC106503510 Gene ID: 106503510<br>KIF13A kinesin family member 13A Gene ID: 102171675

In bold, genes most likely under selection; [1]: signatures also identified by Serranito et al. (2021); SNPs showing p-value<10^{-7} are indicated for each signature; *SNPs showing p-value<1.10^{-9}

Supplementary Table 5

Breeds West-Central Africa	country	latitude	ann.mean.temp	max.temp	min.temp	annual.prec	prec.wet	prec.dry	prec.seas	Altitude
DJA	Burkina Faso	11,51	276,66	380,06	168,95	909,43	241,34	0,85	106,78	318,08
CAM	Cameroon	9,80	268,12	379,74	157,77	959,50	236,54	0,00	110,85	392,17
WAD	Cameroon	4,75	248,88	312,43	198,25	2704,70	443,95	26,19	64,35	379,69
WADn	Nigeria	7,23	266,88	346,54	196,10	1531,44	268,16	7,89	73,44	164,37
RSK	Nigeria	10,38	260,53	368,88	147,45	1027,13	263,36	0,12	109,65	441,67
SHL	Nigeria	12,55	267,54	392,79	127,61	538,94	194,91	0,01	142,28	347,19
NAI	Mali	12,45	273,30	383,54	149,31	972,33	278,90	0,23	116,58	326,60
SDN	Mali	15,37	284,04	406,38	144,71	378,10	134,48	0,00	143,31	287,47

Breeds East Africa	country	latitude	ann.mean.temp	max.temp	min.temp	annual.prec	prec.wet	prec.dry	prec.seas	Altitude
BUR	Burundi	-3,60	188,86	261,64	112,46	1279,74	202,59	3,70	64,09	1633,90
GAL/SEAK	Kenya	2,98	266,45	353,70	191,09	337,06	84,28	4,28	81,65	590,59
GOG	Tanzania	-5,14	209,80	295,77	115,43	655,74	129,39	1,77	89,69	1254,94
MAA	Tanzania	-4,34	214,13	300,88	129,99	663,21	147,93	5,93	80,39	1071,33
SNJ	Tanzania	-3,03	201,55	275,04	125,59	750,51	145,16	3,30	79,02	1520,78
KAR	Uganda	2,66	231,66	318,24	161,81	832,99	125,92	13,50	55,10	1190,58
MUB	Uganda	0,12	214,92	284,35	149,45	1003,20	143,35	33,71	41,84	1224,03
NGD	Uganda	0,64	219,16	292,09	154,14	1272,43	176,78	51,38	34,79	1139,19
SEAU	Uganda	2,20	234,56	328,19	163,68	1283,60	189,33	19,74	49,49	1062,41
SEB	Uganda	1,58	231,46	316,55	161,46	1164,00	165,59	26,38	47,90	1079,73

Altitude in meters, temp.: temperature in °C×10, prec.: precipitation in millimeters, min.: minimal, max.: maximal, seas.: seasonality; ann. mean: annual mean.

East African admixture analysis, including exotic breeds and crossbred individuals

We complete the Admixture analysis presented in the manuscript (Figure 5) by performing another one that includes not only the local breeds of the East African dataset (Table 2) but also some of the exotic breeds found in East Africa and crossbred goats, available in the goat Adaptmap database (Bertollini et al., 2018). In detail, we have added: the Boers breed, that was developed in South Africa in the early 1900s (BOER: 8 individuals sampled in Uganda and 4 in Tanzania); the Saanen breed, which originated in Switzerland (SAA: 2 individuals sampled in Kenya and 19 sampled in Tanzania); and the Toggenburg breed, another Swiss breed (TOG: 22 individuals sampled in Tanzania). Moreover, the Adaptmap dataset included SEA individuals crossbred with either an exotic breed (Alpine, Saanen, or Toggenburg) or the Kenyan Galla, which were added to the analysis. These crossbred individuals were sampled in Kenya (SEAKx, n=30).

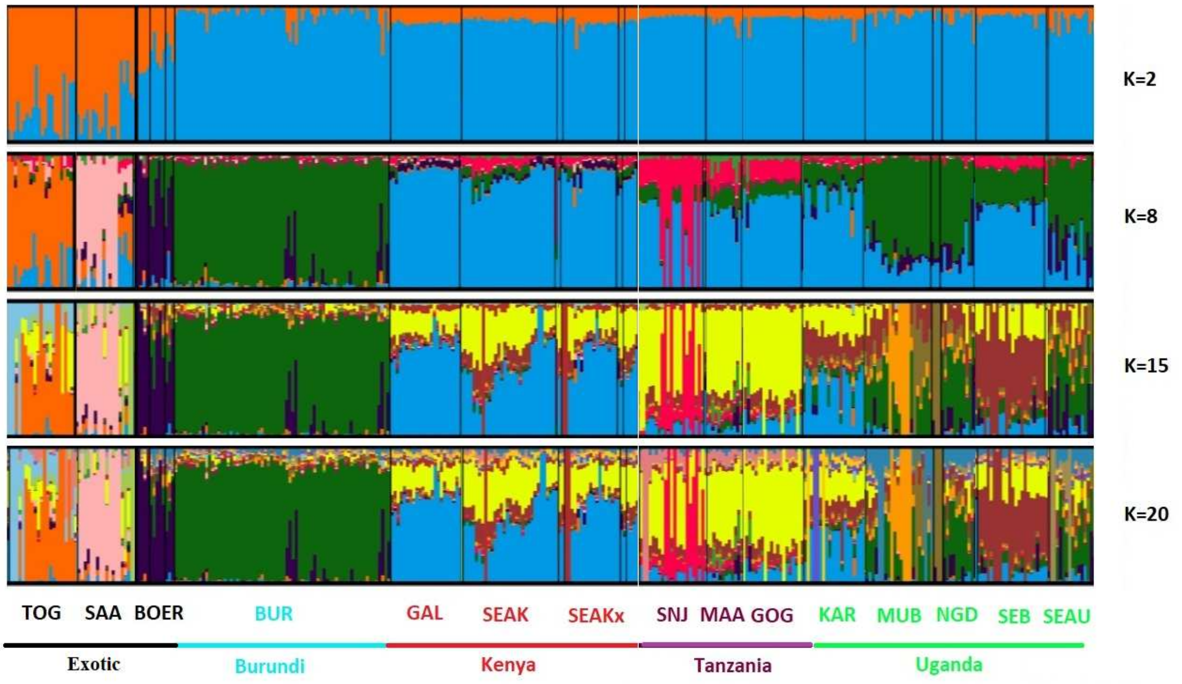

Figure 1. Bayesian clustering performed with ADMIXTURE software on the East African goat dataset, exotic breeds and crossbred individuals. K = number of clusters

The admixture analysis (Figure 1, above) shows that the exotic breeds as well as the BUR from Burundi clearly stand out, while the breeds from Kenya, Uganda and Tanzania share high levels of admixture. In particular, the Kenyan breeds SEA and GAL appear absolutely undifferentiated from the crossbred individuals, SEAKx. We can also note evidence of introgression of the Boer breed in Burundi and Uganda.

The indigenous breeds of East Africa have emerged from pastoral systems, i.e. systems organized around tribes often nomadic, characterized by their own herd management and a system of control of reproductive stock linked to the social organization (Digard et al., 1996; Ben Hounet et al., 2016). The singularity of the breeding systems coupled with very diverse environments, and complex migratory histories have allowed the emergence of a great diversity of breeds, with defined phenotypes and genetic makeup (Wilson, 2004).

If we examine the case of the Kenyan breeds: The Galla breed is raised, by descendants of Borana, a subethnic section of the Oromo people, as some migrated from the southern Ethiopian highlands in the 1500s to northern Kenya (Baxter, 1954). Its origin is probably related to the descent of Arabian goats in Somalia introduced directly from Arabia. The Kenyan SEA is raised by the Maasai people, a Nilo-Saharan ethnic group, who live along the Great Rift Valley, on semi-arid and arid lands, since 17th century CE (Bernsten, 1976). It turns out that these two old native breeds, showing different origins, histories and geographic historical cradles, now appear to be genetically undifferentiated from each other, and undifferentiated from crossbred individuals. This is particularly regrettable, as these breeds have been identified as having some tolerance to the trypanosome (Gall, 1991). This genetic homogenization affects the three countries, Kenya, Tanzania and Uganda and thus concerns a total area of 1,766,491 km².

References

Baxter, P T W. (1954). Social Organization of The Boran of North Kenya. Oxford, UK: Lincoln College.

Ben Hounet, Y., Brisebarre, A.-M., & Guinand, S. (2016). Le patrimoine culturel du pastoralisme : perspective globale, identité étatique et savoirs locaux au prisme des races locales au Maroc: -EN- The cultural heritage of pastoralism - local knowledge, state identity and the global perspective: the example of local breeds in Morocco -FR- -ES- El patrimonio cultural del pastoreo: perspectiva global, identidad estatal y saberes locales vistos desde la óptica de las razas locales en Marruecos. Revue Scientifique et Technique de l'OIE, 35(2), 357–370. doi: 10.20506/rst.35.2.2523

Bernsten, J.L. (1976). The Maasai and Their Neighbors: Variables of Interaction. African Economic History, 2, 1-11.

Bertolini, F., Servin, B., Talenti, A., Rochat, E., Kim, E. S., Oget, C., … the AdaptMap consortium. (2018). Signatures of selection and environmental adaptation across the goat genome post-domestication. Genetics Selection Evolution, 50(1), 57. doi: 10.1186/s12711-018-0421-y

Digard, J.-P., Landais, E., Lhoste, P. (1993). La crise des sociétés pastorales. Un regard pluridisciplinaire. Revue Elev. Méd. Vét. Pays trop., 46(4), 683-692.

Gall C.F. (1991). Breed differences in adaptation of goats. In: Genetic Resources of Pig, Sheep and Goat (Ed. by K. Maijala), pp. 413–29. Elsevier, Amsterdam, The Netherlands.

Wilson, R. T. (2004). Small Ruminant Production and the Small Ruminant Genetic Resource in Tropical Africa. Rome: Food & Agriculture Organization of the United Nations.
